# Higher-Order Correlations Between Thermodynamic Fluctuations in Compressible Aerodynamic Turbulence

**DOI:** 10.3390/e27111103

**Published:** 2025-10-25

**Authors:** Georges A. Gerolymos, Isabelle Vallet

**Affiliations:** Faculty of Science and Engineering, Sorbonne Université, 4 Place Jussieu, 75005 Paris, France

**Keywords:** turbulent boundary layers, compressible boundary layer, turbulence theory

## Abstract

This paper studies the exact and approximate relations between fluctuations in thermodynamic variables (pressure, density and temperature) that are imposed by the dilute-gas (Z=1) equation-of-state (EoS), which is a satisfactory approximation of air thermodynamics for a wide range of pressures and temperatures. It focuses on triple- and higher-order correlations, extending previous studies that concentrated on second-order moments, with emphasis on the mathematical relations, which are generally valid independently of the particular flow configuration. Exact equations are developed both involving only single-variable moments and relating the correlations between variables. These contain nonlinear terms generated by the density-temperature fluctuation product in the fluctuating EoS. The importance of the nonlinear terms in the 6 exact equations between the 10 third-order moments is assessed using DNS (direct numerical simulation) data for compressible turbulent plane channel (TPC) flows and analyzed using general statistical inequalities involving third-order and fourth-order moments. The corresponding linearized system between third-order moments is studied to determine approximate relations and 4-tuples of linearly independent moments. These mathematical tools are then used to analyze TPC flow DNS data on the triple correlations between the thermodynamic variables.

## 1. Introduction

The analysis of turbulence-induced fluctuations in thermodynamic variables is essential for advancing our understanding of the complex interactions occuring in compressible (high-speed) aerodynamic flows. Direct numerical simulation (DNS) can provide detailed information on the variances of and correlations between thermodynamic fluctuations [1,2,3,4]. Such data can be found in several studies [1,2,3,4,5,6,7,8,9,10,11]. Systematic tabulation of the profiles of all correlations up to third-order and of all single-variable central moments up to sixth-order for compressible turbulent plane channel (TPC) flows was recently released in [12]. The availability of detailed DNS data for the correlations between thermodynamic fluctuations renews the interest in developing the exact theoretical relations needed to analyze these results.

Generally, DNS calculations [1,2,3,4,5,6,7,8,9,10,11] adopt the dilute-gas equation-of-state (EoS) ([13], pp. 1–38)

(1) $$\begin{array}{c} Z\left(\rho ,T\right):=\frac{p}{\rho R_{g}T}=1 \end{array}$$

where *p* is the pressure, ρ is the density, *T* is the temperature, $R_{g}$ is the gas constant and Z is the compressibility factor. EoS (1) implies relations between mean values $\left\{\bar{p},\bar{\rho },\bar{T}\right\}$ (6a) and instantaneous fluctuations $\left\{p^{\prime },\rho^{\prime },T^{\prime }\right\}$ (7), where $\left(\cdot \right)=\bar{\left(\cdot \right)}+{\left(\cdot \right)}^{\prime }=\tilde{\left(\cdot \right)}+{\left(\cdot \right)}^{\prime \prime }$ denote Reynolds averages $\bar{\left(\cdot \right)}$, Reynolds fluctuations ${\left(\cdot \right)}^{\prime }$, Favre averages $\tilde{\left(\cdot \right)}$ and Favre fluctuations ${\left(\cdot \right)}^{\prime \prime }$. The present work assumes that the dilute-gas EoS (1) is a valid approximation for the flows studied. Equally important, this EoS is compatible with the DNS calculations providing the data. The analytical relations between the variances of and correlations between $\left\{p^{\prime },\rho^{\prime },T^{\prime }\right\}$ that are developed in the paper are not affected by the eventual temperature dependence of the specific heat capacity $c_{p}$, recalling ([13], pp. 1–38) that under the assumption of local thermodynamic equilibrium, $Z\;=\;1\;\Rightarrow \;c_{p}\;=\;{cp}_{\mathrm{EoS}}\left(T\right)$. On the other hand, analytical relations for entropy fluctuations $s^{\prime }$ can be obtained [14] as infinite power series of $\left\{p^{\prime },\rho^{\prime },T^{\prime }\right\}$ and include the temperature derivatives of $c_{p}\left(T\right)$ at the mean temperature $\bar{T}$. Notice, however, that these derivatives influence only the higher-order nonlinear terms [4,14].

Identities involving the correlations between the thermodynamic variables can be obtained starting directly from the fluctuating EoS (7). This approach was followed in [4,14] for second-order (2-order) correlations between $\left\{p^{\prime },\rho^{\prime },T^{\prime },s^{\prime }\right\}$. In compressible aerodynamic flows, because of the relations implied by the fluctuating EoS (7), the relative fluctuation intensities $\left\{p_{\mathrm{rms}}^{\prime }/\bar{p},\rho_{\mathrm{rms}}^{\prime }/\bar{\rho },T_{\mathrm{rms}}^{\prime }/\bar{T}\right\}$ are of the same order-of-magnitude [2,4], independently of the Mach number of the flow. The nondimensional level of entropy fluctuations $s_{\mathrm{rms}}^{\prime }/R_{g}$ is also of the same order-of-magnitude [4,14] as the coefficients of variation $\left\{p_{\mathrm{rms}}^{\prime }/\bar{p},\rho_{\mathrm{rms}}^{\prime }/\bar{\rho },T_{\mathrm{rms}}^{\prime }/\bar{T}\right\}$ for the basic thermodynamic variables. Exact relations between 2-order correlations of $\left\{p^{\prime },\rho^{\prime },T^{\prime }\right\}$ are readily obtained ([4], (2.5), p. 452) from (7), and, as usual in the statistical analysis of turbulence [15,16], also contain higher, third-order (3-order) correlations, which are generated by the product $\rho^{\prime }\;T^{\prime }$ appearing in the fluctuating EoS (7). They are therefore nonlinear in fluctuation amplitudes. The term *weakly compressible turbulence* will be used to characterize flow conditions for which the linearization of such expressions, by dropping terms of higher-than-leading-order in the relative fluctuation amplitudes $\left\{p_{\mathrm{rms}}^{\prime }/\bar{p},\rho_{\mathrm{rms}}^{\prime }/\bar{\rho },T_{\mathrm{rms}}^{\prime }/\bar{T},s_{\mathrm{rms}}^{\prime }/R_{g}\right\}$, provides an accurate approximation of the exact relations. For compressible turbulent plane channel (TPC) flows at centerline streamwise Mach number ${\bar{M}}_{{\mathrm{CL}}_{x}}\leq 1.5$, the *weakly compressible turbulence* linearized approximation for 2CCs (correlation coefficients involving 2-order moments) is very accurate [4] and remains reasonably accurate up to ${\bar{M}}_{{\mathrm{CL}}_{x}}\leq 2$. At a higher ${\bar{M}}_{{\mathrm{CL}}_{x}}$, some approximations remain robust (very accurate), whereas others are more fragile in the near-wall region (buffer region $5\;⪅\;y^{★}\;⪅\;40$ where $y^{★}$ is the HCB-scaled nondimensional distance from the wall [17]) where the peaks of $\left\{\rho_{\mathrm{rms}}^{\prime }/\bar{\rho },T_{\mathrm{rms}}^{\prime }/\bar{T}\right\}$ are located [2,4,11,14]. It was shown in [4] that using higher-order approximations by including the leading term of the linearization error restores the accuracy up to the highest available in the DNS database [12] ${\bar{M}}_{{\mathrm{CL}}_{x}}\;≊\;2.5$. It is not obvious to simply define the limit of validity of the linearized approximation, as this depends on the specific correlation studied and also depends on wall-distance, suggesting that a local criterion is necessary. Regarding entropy, the leading terms of the infinite power series expansions of $s^{\prime }/R_{g}$ [14] were used in [4] to obtain relations for $\left\{\bar{s^{\prime 2}},\bar{s^{\prime }p^{\prime }},\bar{s^{\prime }\rho^{\prime }},\bar{s^{\prime }T^{\prime }}\right\}$, whose accuracy with an increasing ${\bar{M}}_{{\mathrm{CL}}_{x}}$ follows the same behavior as that of the $\left\{p^{\prime },\rho^{\prime },T^{\prime }\right\}$ relations. These analyses led to mapping the thermodynamic turbulence structure onto a plane [4]. Notice that approximations valid in specific flows and flow regions can substantially extend these limits [4,18] but are not generally valid, especially very near the wall.

Although second-order moments quantify the average intensity of the fluctuations and provide an initial understanding of the level of correlation between variables [1,4,18], higher-order moments are necessary to provide information on the skewness and flatness (kurtosis) of the probability density function (pdf) and on rare (extreme) events [19]. The exact relations between second-order correlation coefficients (2CCs) [4] involves third-order correlation coefficients (3CCs) in the nonlinear terms that control the fluctuation amplitude effects on the thermodynamic turbulence structure. Furthermore, the difference between Favre-averaged $\tilde{{\left(\cdot \right)}^{\prime \prime 2}}$ and Reynolds-averaged $\bar{{\left(\cdot \right)}^{\prime 2}}$ amplitudes, for any flow quantity (·), is controlled by the 3-order correlation coefficient $c_{\rho^{\prime }{\left(\cdot \right)}^{\prime }{\left(\cdot \right)}^{\prime }}$, and fourth-order statistics provide bounds on 3CCs [20,21,22].

The purpose of the paper is to provide mathematical tools that will help to better understand how the dilute-gas EoS (1) couples the moments and correlations of thermodynamic fluctuations, both for use in the analysis of experimental and DNS data and for the development of models of the thermodynamic turbulence structure. Of course, thermodynamic fluctuations are the result of the flow, and their interaction with the velocity field can be described in a statistical sense using specific transport equations [2], but the study of these interactions is outside of the scope of this paper. The focus is on the exact mathematical relations between the moments and correlations of thermodynamic fluctuations that are implied by the dilute-gas EoS (1) and on their linearized approximations, with emphasis on triple correlations. Analysis of DNS data for specific flows serves to illustrate these relations and to quantify the increase in fluctuation-amplitude-dependent nonlinear effects with an increasing Mach number.

In Section 2, we explain why Reynolds decomposition is preferred in the study of thermodynamic correlations (Section 2.2) and discuss possible approaches to the study of the constraints implied by the EoS (1) on the thermodynamic turbulence structure. In Section 3, we briefly provide a summary of DNS data for compressible TPC, which are used to illustrate the mathematical relations developed in the paper. In Section 4, we develop exact relations between arbitrary *n*-order correlation coefficients (*n*CCs; Section 4.1) and $p^{\prime }$-moments (Section 4.2) and study the linearization error (Section 4.4) and the system of linearized equations between 3CCs (Section 4.5). In Section 5, we develop identities and inequalities involving 3CCs and 4CCs between arbitrary random variables which are applied in Section 6, in conjunction with the equations of Section 4, to $\left(p^{\prime },\rho^{\prime },T^{\prime }\right)$-correlations under the EoS (1). Comprehensive DNS data for the 3CCs of thermodynamic fluctuations for compressible TPC flows are presented in Appendix B. A summary of the findings of this paper is provided in Section 7.

## 2. Fluctuating Equation-of-State

We introduce nondimensional coefficients of variation and correlation coefficients (Section 2.1). Favre fluctuations are not practical for the analysis of higher-order correlations between thermodynamic variables (Section 2.2). Therefore, we use Reynolds fluctuations to express the fluctuating EoS (Section 2.3). Conceptually, the bivariate joint (ρ,T)-pdf contains, under the EoS (1), all necessary information regarding thermodynamic fluctuations (Section 2.4).

### 2.1. Definitions

In line with previous work [4,14], we will describe the thermodynamic turbulence structure using coefficients of variation CVs ([23], (48.1), p. 906) and correlation coefficients (2-order 2CCs or higher *n*-order *n*CCs):

(2a) $$\begin{array}{cccccccc} {C\;V}_{\;{\left(\cdot \right)}^{\prime }} & := & & \frac{{\left(\cdot \right)}_{\mathrm{rms}}^{\prime }}{\bar{\left(\cdot \right)}} & \;;\; & {\left(\cdot \right)}_{\mathrm{rms}}^{\prime } & := & \sqrt{\bar{{\left(\cdot \right)}^{\prime 2}}} \end{array}$$

(2b) $$\begin{array}{cccccccc} c_{{\left(\cdot \right)}^{\prime }{\left[\cdot \right]}^{\prime }} & := & & \frac{\bar{{\left(\cdot \right)}^{\prime }{\left[\cdot \right]}^{\prime }}}{{\left(\cdot \right)}_{\mathrm{rms}}^{\prime }{\left[\cdot \right]}_{\mathrm{rms}}^{\prime }}\;\in \left[-1,1\right] & \;;\; & c_{{\left(\cdot \right)}^{\prime }\cdots {\left[\cdot \right]}^{\prime }} & := & \frac{\bar{{\left(\cdot \right)}^{\prime }\cdots {\left[\cdot \right]}^{\prime }}}{{\left(\cdot \right)}_{\mathrm{rms}}^{\prime }\cdots {\left[\cdot \right]}_{\mathrm{rms}}^{\prime }} \end{array}$$

where rms denotes the root-mean-square (2b), and 2CCs are bounded (2b) by the covariance (Schwarz) inequality ([24], (4.11), p. 71), $|c_{{\left(\cdot \right)}^{\prime }{\left[\cdot \right]}^{\prime }}|\;\leq \;1$. Using the terminology of Frenkiel and Klebanoff [19], skewness S(·)′:=S(·)′3, flatness (kurtosis) F(·)′:=F(·)′4, superskewness S(·)′5 and superflatness F(·)′6 are identified to CCs of the corresponding order:

(2c) S(·)′:=S(·)′3:=(·)′3¯(·)rms′3:=c(·)′(·)′(·)′;F(·)′:=F(·)′4:=(·)′4¯(·)rms′4:=c(·)′(·)′(·)′(·)′

(2d) S(·)′5:=(·)′5¯(·)rms′5:=c(·)′(·)′(·)′(·)′(·)′;F(·)′6:=(·)′6¯(·)rms′6:=c(·)′(·)′(·)′(·)′(·)′(·)′

Frenkiel and Klebanoff [19] also considered hyperskewness S(·)′7 and hyperflatness F(·)′8, but these are not used in this paper.

The turbulence intensity of the thermodynamic fluctuations is quantified by the coefficients of variation $\left\{{C\;V}_{\;p^{\prime }},{C\;V}_{\;\rho^{\prime }},{C\;V}_{\;T^{\prime }}\right\}$. Often, equations contain products of powers of the CVs, and we will define as *n*-order terms (noTs)

(3) $$\begin{array}{c} n\mathrm{oTs}∼O\left({C\;V}_{\;p^{\prime }}^{i}\;{C\;V}_{\;\rho^{\prime }}^{j}\;{C\;V}_{\;T^{\prime }}^{k}\right)\;;\;i,j,k\in Z_{\geq 0}:i+j+k=n\geq 1 \end{array}$$

### 2.2. Favre- and Reynolds-Averaging

Entropy *s*, enthalpy *h* or velocity components $u_{i}$ are Favre-decomposed in transport equations for the mean flow or moments [2]. For any Favre-decomposed flow quantity (·),

(4a) $$\begin{array}{cccc} & \tilde{\left(\cdot \right)} & := & \frac{\bar{\rho (\cdot )}}{\bar{\rho }}=\bar{\left(\cdot \right)}+\frac{\bar{\rho^{\prime }{\left(\cdot \right)}^{\prime }}}{\bar{\rho }}\overset{\left(2\right)}{=}\bar{\left(\cdot \right)}\left(1+c_{\rho^{\prime }{\left(\cdot \right)}^{\prime }}\;{C\;V}_{\;\rho^{\prime }}\;{C\;V}_{\;{\left(\cdot \right)}^{\prime }}\right) \end{array}$$

(4b) $$\begin{array}{cccc} & {\left(\cdot \right)}^{\prime \prime } & := & \left(\cdot \right)-\tilde{\left(\cdot \right)}={\left(\cdot \right)}^{\prime }+\left(\bar{\left(\cdot \right)}-\tilde{\left(\cdot \right)}\right)={\left(\cdot \right)}^{\prime }+\bar{{\left(\cdot \right)}^{\prime \prime }} \end{array}$$

(4c) $$\begin{array}{cccc} & \frac{\bar{{\left(\cdot \right)}^{\prime \prime }}}{\bar{\left(\cdot \right)}} & = & -\frac{\bar{\rho^{\prime }{\left(\cdot \right)}^{\prime }}}{\bar{\rho }\;\bar{\left(\cdot \right)}}=-\frac{\bar{\rho^{\prime }{\left(\cdot \right)}^{\prime }}}{\sqrt{\bar{\rho^{\prime 2}}}\sqrt{\bar{{\left(\cdot \right)}^{\prime 2}}}}\frac{\sqrt{\bar{\rho^{\prime 2}}}}{\bar{\rho }}\;\frac{\sqrt{\bar{{\left(\cdot \right)}^{\prime 2}}}}{\bar{\left(\cdot \right)}}\overset{\left(2\right)}{=}-c_{\rho^{\prime }{\left(\cdot \right)}^{\prime }}\;{C\;V}_{\;\rho^{\prime }}\;{C\;V}_{\;{\left(\cdot \right)}^{\prime }} \end{array}$$

(4d) $$\begin{array}{cccc} & \bar{{\left(\cdot \right)}^{\prime }{\left[\cdot \right]}^{\prime \prime }} & = & \bar{{\left(\cdot \right)}^{\prime \prime }{\left[\cdot \right]}^{\prime }}=\bar{{\left(\cdot \right)}^{\prime }{\left[\cdot \right]}^{\prime }} \end{array}$$

including the important identity (4d) for mixed 2-moments [25,26]. Notice that although using Favre decomposition for the temperature $T\;=\;\tilde{T}\;+\;T^{\prime \prime }$ greatly simplifies the expression of the fluctuating EoS, replacing (7) with (A1b), it does not lead to a simplification of the relations between the moments of thermodynamic fluctuations, as explained using the counterexample in Appendix A. Furthermore, the single-variable statistics and pdfs that correspond to Reynolds fluctuations offer considerable insight into the thermodynamic turbulence structure [1,4,14]. Therefore, Reynolds fluctuations $\left\{p^{\prime },\rho^{\prime },T^{\prime }\right\}$ are invariably considered in this paper.

Notice the following useful relation (A4c) obtained during these calculations

(5) $$\begin{array}{c} \tilde{{\left(\cdot \right)}^{\prime \prime 2}}\overset{\left(A4c)\mathrm{and}\;(2a\right)}{=}\bar{{\left(\cdot \right)}^{\prime 2}}\;\left(1+c_{\rho^{\prime }{\left(\cdot \right)}^{\prime }{\left(\cdot \right)}^{\prime }}\;{C\;V}_{\;\rho^{\prime }}-c_{\rho^{\prime }{\left(\cdot \right)}^{\prime }}^{2}\;{C\;V}_{\;\rho^{\prime }}^{2}\right) \end{array}$$

showing that for any flow variable (·), the departure of the ratio of Favre-to-Reynolds fluctuation amplitudes $\tilde{{\left(\cdot \right)}^{\prime \prime 2}}/\bar{{\left(\cdot \right)}^{\prime 2}}$ involves the 3CC $c_{\rho^{\prime }{\left(\cdot \right)}^{\prime }{\left(\cdot \right)}^{\prime }}$.

### 2.3. Dilute-Gas Equation-of-State

Straightforward manipulation of the EoS (1), using the basic properties of Favre averaging (4), yields

(6a) $$\begin{array}{cc} Z\left(\rho ,T\right)=1\overset{\left(1\right)}{\Rightarrow } & \bar{p}\overset{\left(4a\right)}{=}\bar{\rho }R_{g}\tilde{T}\overset{\left(4a\right)}{=}\bar{\rho }R_{g}\bar{T}+R_{g}\;\bar{\rho^{\prime }T^{\prime }} \end{array}$$

Using the general relations

(6b) $$\begin{array}{cc} & \tilde{T}\overset{\left(4b\right)}{=}\bar{T}-\bar{T^{\prime \prime }}\overset{\left(4c\right)}{=}\bar{T}\left(1+\frac{\bar{\rho^{\prime }T^{\prime }}}{\bar{\rho }\bar{T}}\right)\overset{\left(2\right)}{=}\bar{T}\left(1+c_{\rho^{\prime }T^{\prime }}\;{C\;V}_{\;\rho^{\prime }}\;{C\;V}_{\;T^{\prime }}\right) \end{array}$$

(6c) $$\begin{array}{cc} & \bar{T^{\prime \prime }}\overset{\left(4c\right)}{=}-\frac{\bar{\rho^{\prime }T^{\prime }}}{\bar{\rho }}\overset{\left(2\right)}{=}-c_{\rho^{\prime }T^{\prime }}\;{C\;V}_{\;\rho^{\prime }}\;{C\;V}_{\;T^{\prime }}\;\bar{T} \end{array}$$

and substracting (6a) from the instantaneous EoS yields the fluctuating EoS

(7) $$\begin{array}{c} \left(1\right)\mathrm{and}\;\left(6\right)\Rightarrow \frac{\tilde{T}}{\bar{T}}\frac{p^{\prime }}{\bar{p}}=\frac{\rho^{\prime }}{\bar{\rho }}+\frac{T^{\prime }}{\bar{T}}+\frac{\rho^{\prime }T^{\prime }-\bar{\rho^{\prime }T^{\prime }}}{\bar{\rho }\bar{T}} \end{array}$$

Because of the nonlinearity contained in the product ρT in the dilute-gas EoS (1), the fluctuating EoS written in terms of the fluctuations divided by the mean values $\left\{p^{\prime }/\bar{p},\rho^{\prime }/\bar{\rho },T^{\prime }/\bar{T}\right\}$ contains not only the nonlinear term $\rho^{\prime }T^{\prime }$ but also the ratio $\tilde{T}/\bar{T}$ (6b). Both these nonlinear terms are related to the correlation coefficient $c_{\rho^{\prime }T^{\prime }}$, which, weighted by the product of the coefficients of variation ${C\;V}_{\;\rho^{\prime }}\;{C\;V}_{\;T^{\prime }}$, quantifies the relative difference between Favre-averaged $\tilde{T}$ and Reynolds-averaged $\bar{T}$ (6b), induced by the nonlinear (strong) compressible turbulence effects.

Using CVs (2a) and CCs (2b), the fluctuating dilute-gas EoS (7) can be rewritten as

(8) $$\begin{array}{c} \left(7\right)\overset{\left(2a),(2b),(4a\right)}{\Leftrightarrow }\left(1+c_{\rho^{\prime }T^{\prime }}\;{C\;V}_{\;\rho^{\prime }}\;{C\;V}_{\;T^{\prime }}\right)\frac{p^{\prime }}{\bar{p}}=\frac{\rho^{\prime }}{\bar{\rho }}+\frac{T^{\prime }}{\bar{T}}+\frac{\rho^{\prime }}{\bar{\rho }}\frac{T^{\prime }}{\bar{T}}-c_{\rho^{\prime }T^{\prime }}\;{C\;V}_{\;\rho^{\prime }}\;{C\;V}_{\;T^{\prime }} \end{array}$$

Notice that the term $\tilde{T}/\bar{T}\;\overset{\left(4a\right)}{=}\;\left(1+c_{\rho^{\prime }T^{\prime }}\;{C\;V}_{\;\rho^{\prime }}\;{C\;V}_{\;T^{\prime }}\right)$ appears on the LHS of the fluctuating EoS (7) and (8) and will therefore appear in all exact relations between the moments and correlation coefficients. To simplify the notation, we will define the auxiliary weighted coefficient

(9) $$\begin{array}{c} C\;||V_{p^{\prime }}:=\left(1+c_{\rho^{\prime }T^{\prime }}\;{C\;V}_{\;\rho^{\prime }}\;{C\;V}_{\;T^{\prime }}\right)\;{C\;V}_{\;p^{\prime }}\overset{\left(6b\right)}{=}\frac{\tilde{T}}{\bar{T}}\;{C\;V}_{\;p^{\prime }}>0\;\Rightarrow \;C\;||V_{p^{\prime }}∼{C\;V}_{\;p^{\prime }}\left(1+O({C\;V}_{\;\rho^{\prime }}\;{C\;V}_{\;T^{\prime }})\right) \end{array}$$

which includes the compressible ratio $\tilde{T}/\bar{T}$ (4a) and whose difference from ${C\;V}_{\;p^{\prime }}$ is of the second order in fluctuation amplitudes (9).

The fluctuating EoS (7) is the generating equation for the relations developed in this paper. Additionally, we report in Appendix C the identities obtained from an infinite series expansion of the instantaneous EoS (1), which also illustrate the increasing importance of high-order moments with an increasing fluctuation intensity.

### 2.4. A Conceptual Joint (ρ,T)-pdf Approach

Although we will not use an approach based on joint probability density functions (pdfs) in this paper, it seems worthwhile to discuss, conceptually, why the knowledge of joint statistics of only two thermodynamic variables, e.g., density and temperature (ρ,T), suffices to calculate exactly the statistics and correlations of all of the other thermodynamic variables. A bivariate EoS [27] describes the thermodynamic behavior of (dry) air, for a wide range of temperatures (60K≤T≤2000K) and pressures (p≤2000MPa), quite accurately. Because of the equation-of-state (EoS), there are only two independent thermodynamic variables, e.g., density and temperature (ρ,T). Therefore, the knowledge, at a point $\vec{x}$ in the flow, of the joint probability density function (pdf) $f_{\rho T}$ of the instantaneous density ρ and temperature *T* suffices to determine any correlation coefficient between thermodynamic variables at this point. Regarding, e.g., pressure *p*, the knowledge of an EoS relation $T\;=\;T_{\mathrm{EoS}}\left(\rho ,p\right)$ suffices, formally, to calculate ([28], (10), p. 26) the joint pdf $f_{\rho p}\left(\rho ,p\right)$ or the single-variable pdf $f_{p}\left(p\right)$ from the joint pdf $f_{\rho T}\left(\rho ,T\right)$. Adopting the usual thermodynamic formalism ([29], pp. 97–102) for the transformation-of-variables Jacobian, the joint (ρ,p)-pdf reads as

(10a) $$\begin{array}{cc} f_{\rho p}\left(\rho ,p\right)= & |\frac{\partial (\rho ,T)}{\partial (\rho ,p)}|\;f_{\rho T}\left(\rho ,T_{\mathrm{EoS}}\left(\rho ,p\right)\right)=\left|{\left(\frac{\partial T}{\partial p}\right)}_{\rho }\right|\;f_{\rho T}\left(\rho ,T_{\mathrm{EoS}}\left(\rho ,p\right)\right) \end{array}$$

where the partial derivative is of course calculated at state $\left(p,\rho ,T\right)\;=\;(p,\rho ,T_{\mathrm{EoS}}\left(\rho ,p\right))$, and the single-variable pdf $f_{p}\left(p\right)$ is readily obtained through integration

(10b) $$\begin{array}{cc} f_{p}\left(p\right)= & \int_{0}^{+\infty }f_{\rho p}(\rho ,p)\;d\rho \end{array}$$

with the positivity of density ρ setting the lower bound of the integral. For the dilute-gas (Z=1) EoS (1), the Jacobian in (10a) simplifies to

(11) $$\begin{array}{c} \left(1\right)\overset{\left(10\right)}{\Rightarrow }f_{\rho p}\left(\rho ,p\right)=\frac{1}{\rho R_{g}}\;f_{\rho T}\left(\rho ,\frac{p}{\rho R_{g}}\right) \end{array}$$

However, relations (11) would require very careful numerical implementation, (a) sampling $f_{\rho T}\left(\rho ,T\right)$ on a sufficiently large domain of $\left(\rho^{\prime },T^{\prime }\right)$-events ensuring that extreme $p^{\prime }$-events are included and (b) the use of a 2-D reconstruction procedure [30] to transform the bin-sampled pdf into a continuous function (this is necessary in order to create a homogeneous set of *p*-bins from a homogeneous grid of 2-D (ρ,T)-bins). However, this general relation does not provide insight into the basic $p^{\prime }$-moments ($\bar{p^{\prime 2}}$, $\bar{p^{\prime 3}}$, $\bar{p^{\prime 4}}$, ⋯) or coefficients of correlation with the other thermodynamic variables ($c_{p^{\prime }\rho^{\prime }}$, $c_{p^{\prime }T^{\prime }}$, $c_{s^{\prime }p^{\prime }}$, ⋯). Furthermore, the available joint-pdfs in the compressible turbulent flow database [12] which could be used to illustrate the relations developed in this paper were not sampled on a sufficiently large domain of $\left(\rho^{\prime },T^{\prime }\right)$-events for the above procedure (11) to be applicable with sufficient accuracy. A similar analysis applies for alternative choices of the independent variables, e.g., (p,T) or (p,ρ), although the dilute-gas EoS (1) clearly advocates in favor of (ρ,T) as independent variables, and probably so does the general bivariate thermodynamic behavior of air near the dew/bubble boundary [27] as well.

Because of these difficulties, we examine correlations between the thermodynamic variables starting directly from the fluctuating EoS (7).

## 3. Thermodynamic Turbulence Structure in Compressible Plane Channel Flow

**Figure 1:**
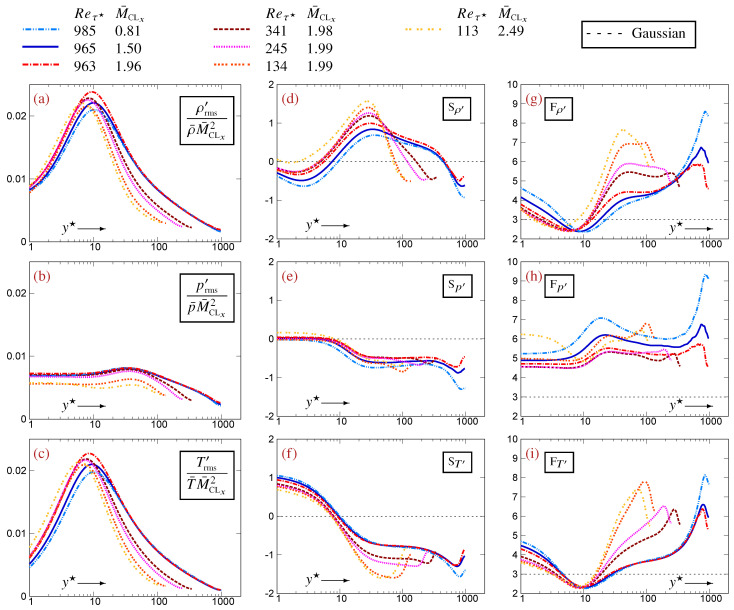
Profiles of coefficients of variation $\left\{{C\;V}_{\;\rho^{\prime }}\;\left(a\right),{C\;V}_{\;p^{\prime }}\;\left(b\right),{C\;V}_{\;T^{\prime }}\;\left(c\right)\right\}$ (scaled by ${\bar{M}}_{{\mathrm{CL}}_{x}}^{2}$ [2]), of skewness $\left\{S_{\rho^{\prime }}\;\left(d\right),S_{p^{\prime }}\;\left(e\right),S_{T^{\prime }}\;\left(f\right)\right\}$ and of kurtosis $\left\{F_{\rho^{\prime }}\;\left(g\right),F_{p^{\prime }}\;\left(h\right),F_{T^{\prime }}\;\left(i\right)\right\}$ for thermodynamic fluctuations, plotted against the HCB-scaled nondimensional wall-distance $y^{★}$ (logscale), using DNS data [12] for compressible turbulent plane channel flows in the range ${Re}_{\tau^{★}}\in \left[113,985\right]$ and ${\bar{M}}_{{\mathrm{CL}}_{x}}\in \left[0.81,2.49\right]$.

**Figure 2:**
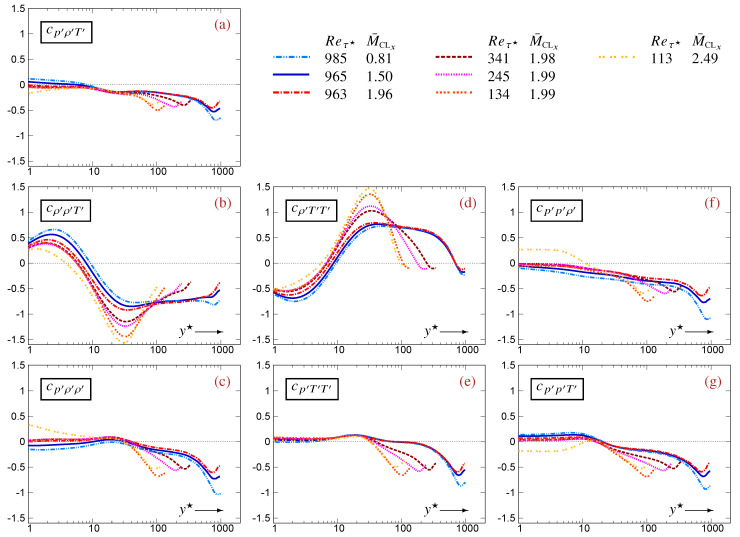
Profiles of 3CCs $\left\{c_{p^{\prime }\rho^{\prime }T^{\prime }}\;\left(a\right),c_{\rho^{\prime }\rho^{\prime }T^{\prime }}\;\left(b\right),c_{p^{\prime }\rho^{\prime }\rho^{\prime }}\;\left(c\right),c_{\rho^{\prime }T^{\prime }T^{\prime }}\;\left(d\right),c_{p^{\prime }T^{\prime }T^{\prime }}\;\left(e\right),c_{p^{\prime }p^{\prime }\rho^{\prime }}\;\left(f\right),c_{p^{\prime }p^{\prime }T^{\prime }}\;\left(g\right)\right\}$ between thermodynamic fluctuations, plotted against the HCB-scaled nondimensional wall-distance $y^{★}$ (logscale), using DNS data [12] for compressible turbulent plane channel flows in the range ${Re}_{\tau^{★}}\in \left[113,985\right]$ and ${\bar{M}}_{{\mathrm{CL}}_{x}}\in \left[0.81,2.49\right]$.

The identities and approximations presented in the paper are assessed or illustrated using DNS data [12] for a turbulent plane channel flow [10,11]. Knowledge of the general behavior of the profiles of the high-order correlations between thermodynamic variables that are considered in the paper is necessary in order to understand these examples. The data for representative flows (${Re}_{\tau^{★}}\;\in \;\left[113,983\right]$ and ${\bar{M}}_{{\mathrm{CL}}_{x}}\;\in \;\left[0.81,2.49\right]$) are briefly reviewed (Figure 1 and Figure 2). ${Re}_{\tau^{★}}$ is the HCB friction Reynolds number [17], ${\bar{M}}_{{\mathrm{CL}}_{x}}$ is the streamwise Mach number at the channel centerline, and $y^{★}$ is the HCB-scaled nondimensional wall-distance [17]. A systematic examination of the data is reported in Appendix B. One should bear in mind that the compressible TPC flow corresponds to very-cold-wall (VCW) conditions (relative to the centerline) [11] and that the thermodynamic turbulence structure (TTS) as determined from the 2CCs and CVs [4] changes with the thermal wall conditions.

**Figure 3:**
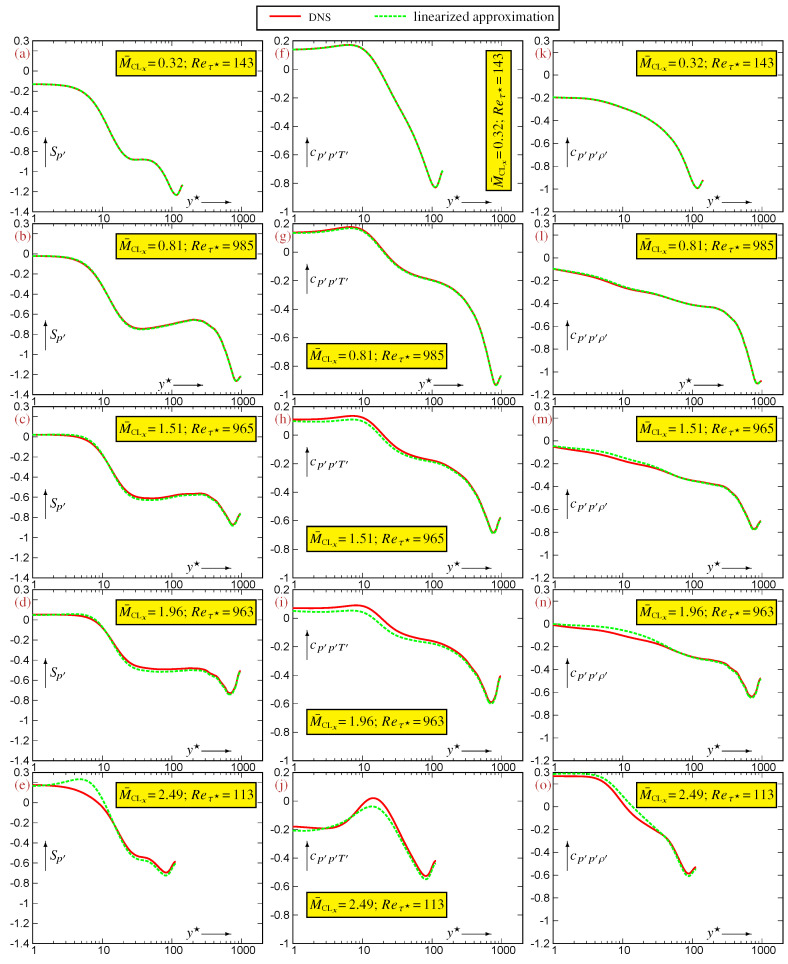
Assessment of the approximate linearized expressions (22a)–(22c) for the 3CCs $\left\{S_{p^{\prime }}\;\left(a–e\right),c_{p^{\prime }p^{\prime }T^{\prime }}\;\left(f–j\right),c_{p^{\prime }p^{\prime }\rho^{\prime }}\;\left(k–o\right)\right\}$, evaluated using DNS data [12] for compressible turbulent plane channel flows in the range ${Re}_{\tau^{★}}\in \left[113,985\right]$ and ${\bar{M}}_{{\mathrm{CL}}_{x}}\in \left[0.32,2.49\right]$, plotted against the HCB-scaled nondimensional wall-distance $y^{★}$ (logscale).

The profiles of $\left\{{C\;V}_{\;\rho^{\prime }},{C\;V}_{\;T^{\prime }}\right\}$ (Figure 1a,c) exhibit a peak close to $y^{★}\;≊\;10$, which extends over most of the buffer region ($5\;⪅\;y^{★}\;⪅\;40$) (Figure 1a,c). These peaks scale approximately with ${\bar{M}}_{{\mathrm{CL}}_{x}}^{2}$ [2], although the exponent is not exacly 2 but probably increases slightly with ${\bar{M}}_{{\mathrm{CL}}_{x}}$ [14] and also has a weak Re dependence. The presence of near-peak values of $\left\{{C\;V}_{\;\rho^{\prime }},{C\;V}_{\;T^{\prime }}\right\}$, that increase approximately with ${\bar{M}}_{{\mathrm{CL}}_{x}}^{2}$, in the entire buffer region ($5\;⪅\;y^{★}\;⪅\;40$) explains the increasing importance of higher-order terms with increasing ${\bar{M}}_{{\mathrm{CL}}_{x}}$ (Appendix C). The level of ${C\;V}_{\;p^{\prime }}$ in the buffer zone is lower than that of $\left\{{C\;V}_{\;\rho^{\prime }},{C\;V}_{\;T^{\prime }}\right\}$ (Figure 1b). It also scales approximately with ${\bar{M}}_{{\mathrm{CL}}_{x}}^{2}$ but additionally has marked Re dependence, in line with the detailed analysis in [10]. The decrease in ${C\;V}_{\;p^{\prime }}$ towards the center of the channel is slower than that for $\left\{{C\;V}_{\;\rho^{\prime }},{C\;V}_{\;T^{\prime }}\right\}$ (Figure 1a–c) so that ${C\;V}_{\;p^{\prime }}$ becomes larger than $\left\{{C\;V}_{\;\rho^{\prime }},{C\;V}_{\;T^{\prime }}\right\}$ in the outer part of the flow ([4], Figure 3, p. 458).

The profiles of skewness $\left\{S_{\rho^{\prime }},S_{p^{\prime }},S_{T^{\prime }}\right\}$ are generally $\in \;[-\frac{3}{2},+\frac{3}{2}]$ (Figure 1d–f). $\left\{S_{\rho^{\prime }},S_{T^{\prime }}\right\}$ vary with both $\left({Re}_{\tau^{★}},{\bar{M}}_{{\mathrm{CL}}_{x}}\right)$ (Figure 1d,f) and exhibit important changes, both in level and sign, with wall-distance. Except near the center of the channel where they are both negative (<0), $\left\{S_{\rho^{\prime }},S_{T^{\prime }}\right\}$ are generally of opposite signs (Figure 1d,f). $S_{p^{\prime }}$ is close to 0 near the wall ($y^{★}\;⪅\;10$) and then drops to a negative value and forms a plateau at ($y^{★}\;≊\;30$) extending up to the wake region (Figure 1e). The $\left({Re}_{\tau^{★}},{\bar{M}}_{{\mathrm{CL}}_{x}}\right)$-effects on $S_{p^{\prime }}$ are less pronounced than those on $\left\{S_{\rho^{\prime }},S_{T^{\prime }}\right\}$ (Figure 1d–f). Notice that the drop in $S_{p^{\prime }}$ from near-0 to negative values at $y^{★}\;≊\;10$ (Figure 1e) occurs very near the location of the $\left({C\;V}_{\;\rho^{\prime }},{C\;V}_{\;T^{\prime }}\right)$-peaks (Figure 1a,c).

**Figure A3:**
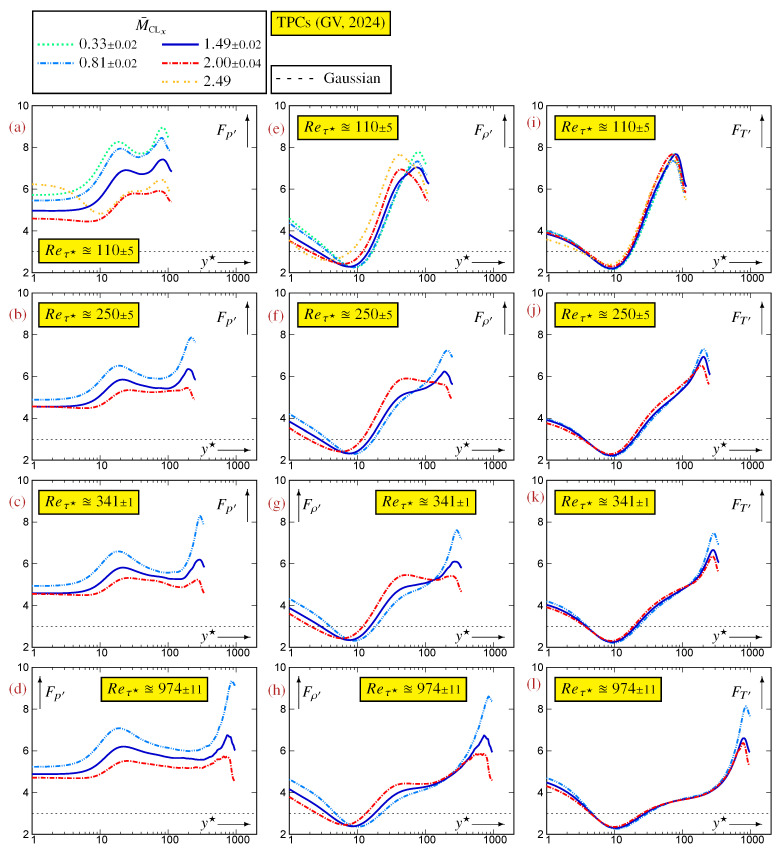
Profiles of kurtosis (flatness) $\left\{F_{p^{\prime }}\;\left(a–d\right),F_{\rho^{\prime }}\;\left(e–h\right),F_{T^{\prime }}\;\left(i–l\right)\right\}$ of thermodynamic fluctuations, plotted against the HCB-scaled nondimensional wall-distance $y^{★}$ (logscale), for varying $0.32\;\leq \;{\bar{M}}_{{\mathrm{CL}}_{x}}\;\leq \;2.49$ at nearly constant $Re_{\tau^{★}}\;\in \;\left\{110,250,340,1000\right\}$, using DNS data [12] for turbulent plane channel flows [10,11].

**Figure A4:**
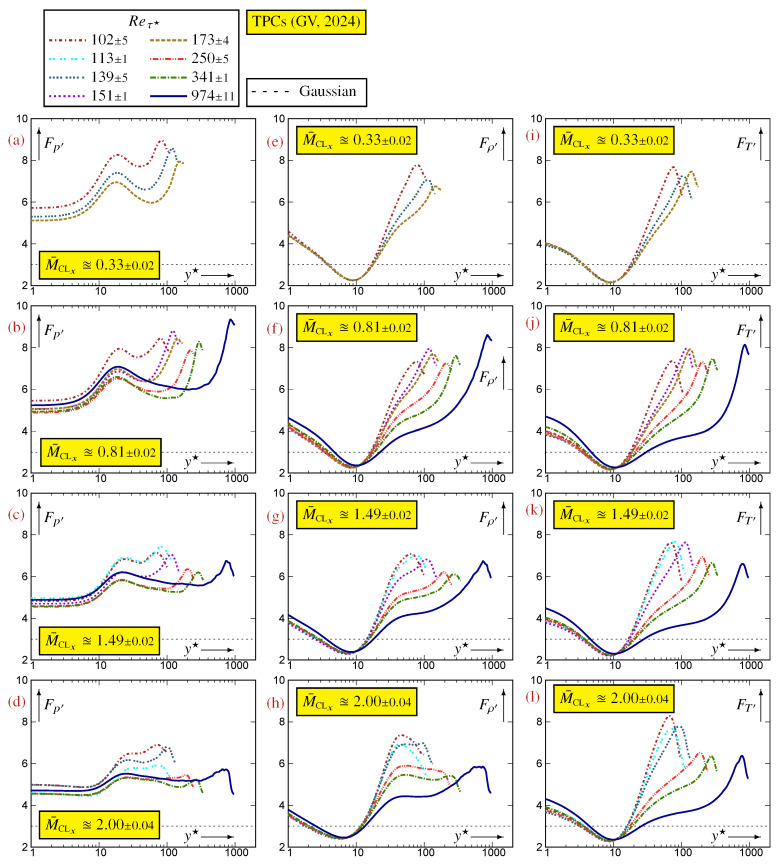
Profiles of kurtosis (flatness) $\left\{F_{p^{\prime }}\;\left(a–d\right),F_{\rho^{\prime }}\;\left(e–h\right),F_{T^{\prime }}\;\left(i–l\right)\right\}$ of thermodynamic fluctuations, plotted against the HCB-scaled nondimensional wall-distance $y^{★}$ (logscale), for varying $97\;\leq \;Re_{\tau^{★}}\;\leq \;985$ at s nearly constant ${\bar{M}}_{{\mathrm{CL}}_{x}}\;\in \;\left\{0.33,0.80,1.50,2.00\right\}$, using DNS data [12] for turbulent plane channel flows [10,11].

The profiles of kurtosis $\left\{F_{\rho^{\prime }},F_{p^{\prime }},F_{T^{\prime }}\right\}$ vary with both $\left({Re}_{\tau^{★}},{\bar{M}}_{{\mathrm{CL}}_{x}}\right)$ (Figure 1g–i). $\left\{F_{\rho^{\prime }},F_{T^{\prime }}\right\}$ exhibit a region of platykurtic distributions (negative excess kurtosis, $F_{{\left(\cdot \right)}^{\prime }}\;<\;3$) at approximately $5\;⪅\;y^{★}\;⪅\;15$ (the boundaries of the $\left(F_{\rho^{\prime }},F_{T^{\prime }}\right)$-platykurtic region vary with ${Re}_{\tau^{★}}$), contrary to $F_{p^{\prime }}$ which is invariably leptokurtic (Figure 1h). Knowledge of kurtosis is important, as it provides bounds for the 3CCs (Section 5). Determination of the precise variation in $\left\{F_{\rho^{\prime }},F_{p^{\prime }},F_{T^{\prime }}\right\}$ with wall-distance and with $\left({Re}_{\tau^{★}},{\bar{M}}_{{\mathrm{CL}}_{x}}\right)$ requires a more systematic examination of the $\left({Re}_{\tau^{★}},{\bar{M}}_{{\mathrm{CL}}_{x}}\right)$-matrix of datasets (Figure A3 and Figure A4).

Regarding the profiles of 3CCs, $\left\{c_{\rho^{\prime }\rho^{\prime }T^{\prime }},c_{\rho^{\prime }T^{\prime }T^{\prime }}\right\}$, which do not contain $p^{\prime }$, are $\in \;[-\frac{3}{2},+\frac{3}{2}]$ (Figure 2b,d). The remaining 3CCs, which involve $p^{\prime }$, $\left\{c_{p^{\prime }\rho^{\prime }T^{\prime }},c_{p^{\prime }\rho^{\prime }\rho^{\prime }},c_{p^{\prime }p^{\prime }\rho^{\prime }},c_{p^{\prime }T^{\prime }T^{\prime }},c_{p^{\prime }p^{\prime }T^{\prime }}\right\}$, are smaller ∈[−1,+1] (Figure 2a,c,e–g), with the trivariate $c_{p^{\prime }\rho^{\prime }T^{\prime }}$ being even smaller $\in \;[-\frac{1}{2},+\frac{1}{2}]$ (Figure 2a). In the near-wall and buffer regions ($y^{★}\;⪅\;40$), all 3CCs containing $p^{\prime }$, $\left\{c_{p^{\prime }\rho^{\prime }T^{\prime }},c_{p^{\prime }\rho^{\prime }\rho^{\prime }},c_{p^{\prime }p^{\prime }\rho^{\prime }},c_{p^{\prime }T^{\prime }T^{\prime }},c_{p^{\prime }p^{\prime }T^{\prime }}\right\}$, are close to 0, dropping progressively to negative values towards the centerline (Figure 2a,c,e–g). Notice, however, the specific behavior of the ${\bar{M}}_{{\mathrm{CL}}_{x}}\;=\;2.49$ data in the near-wall region (Figure 2a,c,e–g), consistent with the observed near-wall compressibility effects induced by organized streamwise pressure waves [10] which increase with an increasing Mach number. Finally, the larger 3CCs, $\left\{c_{\rho^{\prime }\rho^{\prime }T^{\prime }},c_{\rho^{\prime }T^{\prime }T^{\prime }}\right\}$, have generally opposite signs everywhere except very near the centerline (Figure 2b,d).

The above discussion of the data is mostly descriptive. The exact and approximate relations and bounds developed in the following (Section 4 and Section 5) provide the mathematical framework for the analysis of the data that helps explain some of the aforementioned observations.

## 4. Exact and Approximate Relations Between Moments of the Thermodynamic Fluctuations

The fluctuating EoS (7) implies a system of exact relations between trivariate *n*-order moments (Section 4.1) and exact expressions for $\bar{p^{\prime n}}$ in terms of $\left(\rho^{\prime },T^{\prime }\right)$-moments (Section 4.2). Under weakly compressible turbulence conditions, $\max\left({C\;V}_{\;p^{\prime }},{C\;V}_{\;\rho^{\prime }},{C\;V}_{\;T^{\prime }}\right)\;\ll \;1$, these exact relations can be linearized (Section 4.3), leading to approximate structure relations between 3CCs (Section 4.5).

### 4.1. Correlation Coefficients of Order n (nCCs)

Previous work [1,4,14] has concentrated on 2-order moments, which are of major importance for practical turbulent flow analysis. However, the relations between 2-order moments of thermodynamic correlations also include 3-order moments ([4], (2.5), p. 452), which quantify the truncation error of the weakly compressible approximation. Selected 3-order moments relations were also reported in [4] with respect to the study of the approximation error, and these contained 4-order moments. A more systematic approach is followed here.

It is straightforward, starting from the fluctuating EoS (7), to obtain relations for the *n*-moments (and *n*CCs) between the fluctuations in the basic thermodynamic variables $\left\{p^{\prime },\rho^{\prime },T^{\prime }\right\}$. Multiplying (7) by $p^{\prime k}\rho^{\prime \ell }T^{\prime m}$ (with $k+\ell +m\;=\;n-1\;\geq \;1\;;\;k,\ell ,m\in Z_{\geq 0}$) yields, upon averaging the exact relations,

(12) $$\begin{array}{c} \frac{\tilde{T}}{\bar{T}}\frac{\bar{p^{\prime k+1}\rho^{\prime \ell }T^{\prime m}}}{\bar{p}}\overset{\left(7\right)}{=}\frac{\bar{p^{\prime k}\rho^{\prime \ell +1}T^{\prime m}}}{\bar{\rho }}+\frac{\bar{p^{\prime k}\rho^{\prime \ell }T^{\prime m+1}}}{\bar{T}}+\frac{\bar{p^{\prime k}\rho^{\prime \ell +1}T^{\prime m+1}}-\bar{p^{\prime k}\rho^{\prime \ell }T^{\prime m}}\;\bar{\rho^{\prime }T^{\prime }}}{\bar{\rho }\bar{T}}\\ \forall \;k,\ell ,m\in Z_{\geq 0}:k+\ell +m\;=\;n-1\;\geq \;1 \end{array}$$

connecting the *n*-moments and also including a higher (n+1)-moment and a lower (n−1)-moment. Introducing CVs (2a) and (9) and CCs (2b), we obtain the equivalent relation

(13) $$\begin{array}{c} \underset{\mathrm{nCC}}{\underset{︸}{c_{p^{\prime k+1}\rho^{\prime \ell }T^{\prime m}}C\;||V_{p^{\prime }}}}\overset{\left(12),(2),(9\right)}{=}\underset{\mathrm{nCCs}}{\underset{︸}{c_{p^{\prime k}\rho^{\prime \ell +1}T^{\prime m}}{C\;V}_{\;\rho^{\prime }}+c_{p^{\prime k}\rho^{\prime \ell }T^{\prime m+1}}{C\;V}_{\;T^{\prime }}}}+\underset{O({C\;V}_{\;\rho^{\prime }}\;{C\;V}_{\;T^{\prime }})\;\mathrm{HoTs}}{\underset{︸}{(\underset{(n+1)\mathrm{CC}}{\underset{︸}{c_{p^{\prime k}\rho^{\prime \ell +1}T^{\prime m+1}}}}-\underset{(n-1)\mathrm{CC}}{\underset{︸}{c_{p^{\prime k}\rho^{\prime \ell }T^{\prime m}}}}c_{\rho^{\prime }T^{\prime }}){C\;V}_{\;\rho^{\prime }}\;{C\;V}_{\;T^{\prime }}}}\\ \forall \;k,\ell ,m\in Z_{\geq 0}:k+\ell +m\;=\;n-1\;\geq \;1 \end{array}$$

This is the generating equation for the study of the constraints imposed by the dilute-gas EoS (7) on the correlations between fluctuations in the basic thermodynamic variables $\left\{p^{\prime },\rho^{\prime },T^{\prime }\right\}$. It contains a linear $O(C\;||V_{p^{\prime }},{C\;V}_{\;\rho^{\prime }},{C\;V}_{\;T^{\prime }})$ leading-order expression relating *n*CCs (*n*-order moments) and quadratic $O({C\;V}_{\;\rho^{\prime }}\;{C\;V}_{\;T^{\prime }})$ terms containing an (n+1)CC and an (n−1)CC. For the lowest n=2 case, we have k+ℓ+m=n−1=1, so that for n=2, the (n−1)-moment is $\bar{{\left(\cdot \right)}^{\prime }}\;=\;0$. For n>2, the (n−1)-moment in (12) and (13) is generally ≠0.

Of course, since equations between turbulent statistics are an open system, there are ∀n≥2 more unknown *n*CCs than equations (13). For the lowest n=2 case, the moments are $\left\{\bar{p^{\prime 2}},\bar{\rho^{\prime 2}},\bar{T^{\prime 2}},\bar{p^{\prime }\rho^{\prime }},\bar{p^{\prime }T^{\prime }},\bar{\rho^{\prime }T^{\prime }}\right\}$ so that the nondimensional unknowns include the CVs, i.e., they are $\left\{{C\;V}_{\;p^{\prime }},{C\;V}_{\;\rho^{\prime }},{C\;V}_{\;T^{\prime }},c_{p^{\prime }\rho^{\prime }},c_{p^{\prime }T^{\prime }},c_{\rho^{\prime }T^{\prime }}\right\}$.

The products of order n≥2 which appear on the LHS of (12) are of the form $\bar{p^{\prime k+1}\rho^{\prime \ell }T^{\prime m}}$ (with $k+\ell +m\;=\;n-1\;\geq \;1\;;\;k,\ell ,m\in Z_{\geq 0}$). These are combinations with repetition of n−1 objects from a list of three, $\left\{p^{\prime },\rho^{\prime },T^{\prime }\right\}$. There are therefore ([31], p. 744), by construction, 3+n−2n−1=n+1n−1=n(n+1)2 equations. On the other hand, *n*-order moments contain products of the form $\bar{p^{\prime k}\rho^{\prime \ell }T^{\prime m}}$ (with $k+\ell +m\;=\;n\;\geq \;2\;;\;k,\ell ,m\in Z_{\geq 0}$). These are combinations with repetition of *n* objects from a list of three. There are therefore ([31], p. 744) 3+n−1n=n+2n=(n+1)(n+2)2*n*-order moments (Table 1). Solving the system of Equation (13), we can therefore determine $\frac{n(n+1)}{2}$*n*-order moments from the remaining $\frac{\left(n+1)(n+2\right)}{2}\;-\;\frac{n(n+1)}{2}\;=\;n\;+\;1$ independent *n*-order moments (Table 1). The *n*CC terms in the exact Equation (13) between *n*CCs are $O(C\;||V_{p^{\prime }},{C\;V}_{\;\rho^{\prime }},{C\;V}_{\;T^{\prime }})$, and each equation between *n*CCs also involves an $O({C\;V}_{\;\rho^{\prime }}\;{C\;V}_{\;T^{\prime }})$ term containing moments of orders (n+1) and (n−1). This $O({C\;V}_{\;\rho^{\prime }}\;{C\;V}_{\;T^{\prime }})$ term is nonlinear in the sense that it is generated by the nonlinear term ${\left(\rho^{\prime }\;T^{\prime }\right)}^{\prime }\;:=\;\rho^{\prime }\;T^{\prime }\;-\;\bar{\rho^{\prime }\;T^{\prime }}$ appearing in the fluctuating EoS (7).

At the weakly compressible turbulence limit, $\max\left({C\;V}_{\;p^{\prime }},{C\;V}_{\;\rho^{\prime }},{C\;V}_{\;T^{\prime }}\right)\;\ll \;1$, the quadratic $O({C\;V}_{\;\rho^{\prime }}\;{C\;V}_{\;T^{\prime }})$ terms in (13) can be dropped, thus obtaining a linear system between *n*-moments only. The linear system between 2-moments has been studied in [4]. However, for higher-order (n>2) moments, not every choice of (n+1) among the $\frac{\left(n+1)(n+2\right)}{2}$*n*-moments forms a linearly independent set, as shown for 3CCs in Section 4.5.

The study of (13) for 3CCs is reported in Section 4.4, and that for the corresponding linear system at the asymptotic limit of weakly compressible turbulence is reported in Section 4.5.

### 4.2. Central Moments of $p^{\prime }$

Expressions of the central moments of $p^{\prime }$ in terms of other *n*CCs are readily obtained by setting ℓ=m=0 in (12). Central moments of $p^{\prime }$, in line with Section 2.4, are also readily expressed in terms of (ρ,T)-statistics simply by raising the fluctuating EoS (7) to the power *n*. Straightforward calculations, using the multinomial theorem ([32], p. 17) and standard multiple-summation techniques [33,34], yield

(14) T˜T¯np′n¯p¯n=(7)ρ′ρ¯+T′T¯+ρ′T′−ρ′T′¯ρ¯T¯n¯=∑0≤i,j,k,ℓ≤ni+j+k+ℓ=nni,j,k,ℓρ′ρ¯iT′T¯jρ′T′ρ¯T¯k−ρ′T′¯ρ¯T¯ℓ¯=∑i=0n∑j=0n−i∑k=0n−i−jn!i!j!k!(n−i−j−k)!ρ′ρ¯iT′T¯jρ′T′ρ¯T¯k¯−ρ′T′¯ρ¯T¯n−i−j−k=∑i=0n∑j=0n−i∑k=0n−i−j(−1)n−i−j−kn!i!j!k!(n−i−j−k)!ρ′ρ¯i+kT′T¯j+k¯ρ′T′¯ρ¯T¯n−i−j−k

which using CCs and CVs (2) reads as

(15a) $$\begin{array}{c} {\left(\frac{\tilde{T}}{\bar{T}}\right)}^{\;\;n}\;\frac{\bar{p^{\prime n}}}{{\bar{p}}^{n}}\overset{\left(14)\mathrm{and}(2\right)}{=}\sum_{i=0}^{n}\sum_{j=0}^{n-i}\;\sum_{k=0}^{n-i-j}\left[\frac{{\left(-1\right)}^{n-i-j-k}\;n!}{i!\;j!\;k!\;(n\;-\;i\;-\;j\;-\;k)!}\;c_{{\rho^{\prime }}^{i+k}T^{\prime j+k}}\;c_{\rho^{\prime }T^{\prime }}^{n-i-j-k}\;{C\;V}_{\;\rho^{\prime }}^{n-j}\;{C\;V}_{\;T^{\prime }}^{n-i}\right] \end{array}$$

where we extend the general definitions (2), valid for ij≥1, by re-expressing $c_{{\rho^{\prime }}^{i}T^{\prime j}}$ in terms of standardized variables

(15b) $$\begin{array}{c} c_{{\rho^{\prime }}^{i}{T^{\prime }}^{j}}:=\bar{{\left(\frac{\rho^{\prime }}{\sqrt{\bar{\rho^{\prime 2}}}}\right)}^{\;\;i}{\left(\frac{T^{\prime }}{\sqrt{\bar{T^{\prime 2}}}}\right)}^{\;\;j}}\Rightarrow \left\{\begin{array}{c} c_{{\rho^{\prime }}^{0}T^{\prime 0}}=1\\ c_{\rho^{\prime }T^{\prime 0}}=c_{{\rho^{\prime }}^{0}T^{\prime }}=0 \end{array}\right. \end{array}$$

and by (9),

(15c) $$\begin{array}{c} {\left(\frac{\tilde{T}}{\bar{T}}\right)}^{\;\;n}\;\frac{\bar{p^{\prime n}}}{{\bar{p}}^{n}}\overset{\left(9)\mathrm{and}\;(2\right)}{=}\frac{\bar{p^{\prime n}}}{p_{\mathrm{rms}}^{\prime n}}{C\;||V}_{p^{\prime }}^{n} \end{array}$$

where $\bar{p^{\prime n}}/p_{\mathrm{rms}}^{\prime n}$, for n≥3, are identified with skewnesses (2c) and (2d) for *n* odd and flatnesses (2c) and (2d) for *n* even. According to (15), the exact general expression of $\bar{p^{\prime n}}$ in terms of $\left(\rho^{\prime },T^{\prime }\right)$-correlations involves up to 2nCCs appearing in terms of $O({C\;V}_{\;\rho^{\prime }}^{n}\;{C\;V}_{\;T^{\prime }}^{n})$, consistently with the conceptual approach of Section 2.4. This approach requires therefore many higher-than-*n*-order CCs to exactly calculate $\bar{p^{\prime n}}$ compared to (12), which only includes an (n+1)-order correlation, but where the other *n*CCs include $p^{\prime }$. The alternative approach (12) is simpler and was followed in [4] to develop linearized approximations for the 2CCs in terms of the coefficients of variation.

It is straightforward to calculate from (15) the relations for ${C\;||V}_{p^{\prime }}^{2}$ (9)

(16) $$\begin{array}{cc} {C\;||V}_{p^{\prime }}^{2}\overset{\left(15)\mathrm{and}\;(2\right)}{=} & {C\;V}_{\;\rho^{\prime }}^{2}+{C\;V}_{\;T^{\prime }}^{2}+2\;c_{\rho^{\prime }T^{\prime }}\;{C\;V}_{\;T^{\prime }}\;{C\;V}_{\;\rho^{\prime }}\\ + & 2\;c_{\rho^{\prime }\rho^{\prime }T^{\prime }}\;{C\;V}_{\;T^{\prime }}\;{C\;V}_{\;\rho^{\prime }}^{2}+2\;c_{\rho^{\prime }T^{\prime }T^{\prime }}\;{C\;V}_{\;T^{\prime }}^{2}\;{C\;V}_{\;\rho^{\prime }}\\ + & \left(c_{\rho^{\prime }\rho^{\prime }T^{\prime }T^{\prime }}-c_{\rho^{\prime }T^{\prime }}^{2}\right)\;{C\;V}_{\;T^{\prime }}^{2}\;{C\;V}_{\;\rho^{\prime }}^{2} \end{array}$$

and for the skewness Sp′=Sp′3 (2c)

(17) $$\begin{array}{cc} S_{p^{\prime }}\;{C\;||V}_{p^{\prime }}^{3}\overset{\left(15)\mathrm{and}\;(2\right)}{=} & S_{\rho^{\prime }}\;{C\;V}_{\;\rho^{\prime }}^{3}+S_{T^{\prime }}\;{C\;V}_{\;T^{\prime }}^{3}+3\;c_{\rho^{\prime }\;\rho^{\prime }T^{\prime }}\;{C\;V}_{\;T^{\prime }}\;{C\;V}_{\;\rho^{\prime }}^{2}+3\;c_{\rho^{\prime }T^{\prime }T^{\prime }}\;{C\;V}_{\;T^{\prime }}^{2}\;{C\;V}_{\;\rho^{\prime }}\\ + & 3\left(c_{\rho^{\prime }\rho^{\prime }\rho^{\prime }T^{\prime }}-c_{\rho^{\prime }T^{\prime }}\right)\;{C\;V}_{\;T^{\prime }}\;{C\;V}_{\;\rho^{\prime }}^{3}+6\left(c_{\rho^{\prime }\rho^{\prime }T^{\prime }T^{\prime }}-c_{\rho^{\prime }T^{\prime }}^{2}\right)\;{C\;V}_{\;T^{\prime }}^{2}\;{C\;V}_{\;\rho^{\prime }}^{2}+3\left(c_{\rho^{\prime }T^{\prime }T^{\prime }T^{\prime }}-c_{\rho^{\prime }T^{\prime }}\right)\;{C\;V}_{\;T^{\prime }}^{3}\;{C\;V}_{\;\rho^{\prime }}\\ + & 3\left(c_{\rho^{\prime }\rho^{\prime }\rho^{\prime }T^{\prime }T^{\prime }}-2\;c_{\rho^{\prime }T^{\prime }}\;c_{\rho^{\prime }\rho^{\prime }T^{\prime }}\right)\;{C\;V}_{\;T^{\prime }}^{2}\;{C\;V}_{\;\rho^{\prime }}^{3}+3\left(c_{\rho^{\prime }\rho^{\prime }T^{\prime }T^{\prime }T^{\prime }}-2\;c_{\rho^{\prime }T^{\prime }}\;c_{\rho^{\prime }T^{\prime }T^{\prime }}\right)\;{C\;V}_{\;T^{\prime }}^{3}\;{C\;V}_{\;\rho^{\prime }}^{2}\\ + & \left(c_{\rho^{\prime }\rho^{\prime }\rho^{\prime }T^{\prime }T^{\prime }T^{\prime }}-3\;c_{\rho^{\prime }T^{\prime }}\;c_{\rho^{\prime }\rho^{\prime }T^{\prime }T^{\prime }}+2\;c_{\rho^{\prime }T^{\prime }}^{3}\right)\;{C\;V}_{\;T^{\prime }}^{3}\;{C\;V}_{\;\rho^{\prime }}^{3} \end{array}$$

illustrating the rapid increase in the number of higher-order correlations when using (15). Each line in (16) and (17) corresponds to an increasingly smaller order of magnitude, with the first line containing the leading-order expression which is approximately used at the weakly compressible limit. Of course, the asymptotic expressions at the weakly compressible turbulence limit are identical for both approaches (13) and (15).

### 4.3. Unscrambling the Exact Relations

It is straightforward to expand relations (13). The three relations between the six second-order moments in Table 1 were given in [4], (2.5), p. 452, and are reproduced for completeness:

(18a) $$\begin{array}{cccccccccc} & C\;||V_{p^{\prime }} & \overset{\left(13\right)}{=}\; & & c_{p^{\prime }\rho^{\prime }}\; & {\mathrm{CV}}_{\rho^{\prime }}+ & c_{p^{\prime }T^{\prime }}\; & {\mathrm{CV}}_{T^{\prime }}+ & c_{p^{\prime }\rho^{\prime }T^{\prime }}\; & {\mathrm{CV}}_{\rho^{\prime }}\;{\mathrm{CV}}_{T^{\prime }} \end{array}$$

(18b) $$\begin{array}{cccccccccc} c_{p^{\prime }\rho^{\prime }}\; & C\;||V_{p^{\prime }} & \overset{\left(13\right)}{=}\; & & & {\mathrm{CV}}_{\rho^{\prime }}+ & c_{\rho^{\prime }T^{\prime }}\; & {\mathrm{CV}}_{T^{\prime }}+ & c_{\rho^{\prime }\rho^{\prime }T^{\prime }}\; & {\mathrm{CV}}_{\rho^{\prime }}\;{\mathrm{CV}}_{T^{\prime }} \end{array}$$

(18c) $$\begin{array}{cccccccccc} c_{p^{\prime }T^{\prime }}\; & C\;||V_{p^{\prime }} & \overset{\left(13\right)}{=}\; & & c_{\rho^{\prime }T^{\prime }}\; & {\mathrm{CV}}_{\rho^{\prime }}+ & & {\mathrm{CV}}_{T^{\prime }}+ & c_{\rho^{\prime }T^{\prime }T^{\prime }}\; & {\mathrm{CV}}_{\rho^{\prime }}\;{\mathrm{CV}}_{T^{\prime }} \end{array}$$

These equations were the basis of the linearized analysis of the thermodynamic turbulence structure in [4], which focused on 2-order moments. The origin of the terms containing the product ${\mathrm{CV}}_{\rho^{\prime }}\;{\mathrm{CV}}_{T^{\prime }}$ on the RHS of (18) is the nonlinear term ${\left(\rho^{\prime }\;T^{\prime }\right)}^{\prime }$ in the fluctuating EoS (7). The linearized approximation drops terms of $O({\mathrm{CV}}_{\rho^{\prime }}\;{\mathrm{CV}}_{T^{\prime }})$ or higher in (18). Therefore, the linearization error of the weakly compressible approximation depends on the 3CCs $\left\{c_{p^{\prime }\rho^{\prime }T^{\prime }},c_{\rho^{\prime }\rho^{\prime }T^{\prime }},c_{\rho^{\prime }T^{\prime }T^{\prime }}\right\}$ in (18).

The six distinct relations between the 10 third-order moments (Table 1) read as (13)

(19a) $$\begin{array}{cccccccccccc} S_{p^{\prime }}\; & C\;||V_{p^{\prime }} & \overset{\left(13\right)}{=}\; & & c_{p^{\prime }p^{\prime }\rho^{\prime }} & \;{C\;V}_{\;\rho^{\prime }}+ & c_{p^{\prime }p^{\prime }T^{\prime }} & \;{C\;V}_{\;T^{\prime }}+ & & (c_{p^{\prime }p^{\prime }\rho^{\prime }T^{\prime }}- & c_{\rho^{\prime }T^{\prime }}) & \;{C\;V}_{\;\rho^{\prime }}\;{C\;V}_{\;T^{\prime }} \end{array}$$

(19b) $$\begin{array}{cccccccccccc} c_{p^{\prime }p^{\prime }T^{\prime }}\; & C\;||V_{p^{\prime }} & \overset{\left(13\right)}{=}\; & & c_{p^{\prime }\rho^{\prime }T^{\prime }} & \;{C\;V}_{\;\rho^{\prime }}+ & c_{p^{\prime }T^{\prime }T^{\prime }} & \;{C\;V}_{\;T^{\prime }}+ & & (c_{p^{\prime }\rho^{\prime }T^{\prime }T^{\prime }}- & c_{p^{\prime }T^{\prime }}\;c_{\rho^{\prime }T^{\prime }}) & \;{C\;V}_{\;\rho^{\prime }}\;{C\;V}_{\;T^{\prime }} \end{array}$$

(19c) $$\begin{array}{cccccccccccc} c_{p^{\prime }p^{\prime }\rho^{\prime }}\; & C\;||V_{p^{\prime }} & \overset{\left(13\right)}{=}\; & & c_{p^{\prime }\rho^{\prime }\rho^{\prime }} & \;{C\;V}_{\;\rho^{\prime }}+ & c_{p^{\prime }\rho^{\prime }T^{\prime }} & \;{C\;V}_{\;T^{\prime }}+ & & (c_{p^{\prime }\rho^{\prime }\rho^{\prime }T^{\prime }}- & c_{p^{\prime }\rho^{\prime }}\;c_{\rho^{\prime }T^{\prime }}) & \;{C\;V}_{\;\rho^{\prime }}\;{C\;V}_{\;T^{\prime }} \end{array}$$

(19d) $$\begin{array}{cccccccccccc} c_{p^{\prime }\rho^{\prime }\rho^{\prime }}\; & C\;||V_{p^{\prime }} & \overset{\left(13\right)}{=}\; & & S_{\rho^{\prime }} & \;{C\;V}_{\;\rho^{\prime }}+ & c_{\rho^{\prime }\rho^{\prime }T^{\prime }} & \;{C\;V}_{\;T^{\prime }}+ & & (c_{\rho^{\prime }\rho^{\prime }\rho^{\prime }T^{\prime }}- & c_{\rho^{\prime }T^{\prime }}) & \;{C\;V}_{\;\rho^{\prime }}\;{C\;V}_{\;T^{\prime }} \end{array}$$

(19e) $$\begin{array}{cccccccccccc} c_{p^{\prime }T^{\prime }T^{\prime }}\; & C\;||V_{p^{\prime }} & \overset{\left(13\right)}{=}\; & & c_{\rho^{\prime }T^{\prime }T^{\prime }} & \;{C\;V}_{\;\rho^{\prime }}+ & S_{T^{\prime }} & \;{C\;V}_{\;T^{\prime }}+ & & (c_{\rho^{\prime }T^{\prime }T^{\prime }T^{\prime }}- & c_{\rho^{\prime }T^{\prime }}) & \;{C\;V}_{\;\rho^{\prime }}\;{C\;V}_{\;T^{\prime }} \end{array}$$

(19f) $$\begin{array}{cccccccccccc} c_{p^{\prime }\rho^{\prime }T^{\prime }}\; & C\;||V_{p^{\prime }} & \overset{\left(13\right)}{=}\; & & c_{\rho^{\prime }\rho^{\prime }T^{\prime }} & \;{C\;V}_{\;\rho^{\prime }}+ & c_{\rho^{\prime }T^{\prime }T^{\prime }} & \;{C\;V}_{\;T^{\prime }}+ & & (c_{\rho^{\prime }\rho^{\prime }T^{\prime }T^{\prime }}- & c_{\rho^{\prime }T^{\prime }}^{2}) & \;{C\;V}_{\;\rho^{\prime }}\;{C\;V}_{\;T^{\prime }} \end{array}$$

Similarly to (18) for the 2CCs, the origin of the terms containing the product ${\mathrm{CV}}_{\rho^{\prime }}\;{\mathrm{CV}}_{T^{\prime }}$ on the RHS of (19) is the nonlinear term ${\left(\rho^{\prime }\;T^{\prime }\right)}^{\prime }$ in the fluctuating EoS (7). The linearized approximation drops terms of $O({\mathrm{CV}}_{\rho^{\prime }}\;{\mathrm{CV}}_{T^{\prime }})$ or higher in (19). Therefore, the linearization error of the weakly compressible turbulence approximation for 3CCs depends on the differences between 4CCs and 2CCs in (19). This approximation error determines in particular the limit of the fluctuation intensities $\left\{{C\;V}_{\;p^{\prime }},{C\;V}_{\;\rho^{\prime }},{C\;V}_{\;T^{\prime }}\right\}$ for which the identities (19) can be linearized with acceptable accuracy. Since the magnitudes of 4CCs are generally larger than those of 3CCs (e.g., skewnesses squared are smaller than flatnesses [35]), we would expect the linearized approximation for 3CCs to become inaccurate at lower Mach numbers (lower fluctuation amplitudes) than the linearized approximation for 2CCs. Furthermore, as the approximation error depends on CCs, it obviously differs from one flow configuration to another.

Similarly, the 10 distinct relations between the 15 fourth-order moments (Table 1) read as (13)

(20a) $$\begin{array}{cccccccccccc} F_{p^{\prime }}\; & C\;||V_{p^{\prime }} & \overset{\left(13\right)}{=}\; & & c_{p^{\prime }p^{\prime }p^{\prime }\rho^{\prime }} & \;{C\;V}_{\;\rho^{\prime }}+ & c_{p^{\prime }p^{\prime }p^{\prime }T^{\prime }} & \;{C\;V}_{\;T^{\prime }}+ & & (c_{p^{\prime }p^{\prime }p^{\prime }\rho^{\prime }T^{\prime }}- & S_{p^{\prime }}\;c_{\rho^{\prime }T^{\prime }}) & \;{C\;V}_{\;\rho^{\prime }}\;{C\;V}_{\;T^{\prime }} \end{array}$$

(20b) $$\begin{array}{cccccccccccc} c_{p^{\prime }p^{\prime }p^{\prime }T^{\prime }}\; & C\;||V_{p^{\prime }} & \overset{\left(13\right)}{=}\; & & c_{p^{\prime }p^{\prime }\rho^{\prime }T^{\prime }} & \;{C\;V}_{\;\rho^{\prime }}+ & c_{p^{\prime }p^{\prime }T^{\prime }T^{\prime }} & \;{C\;V}_{\;T^{\prime }}+ & & (c_{p^{\prime }p^{\prime }\rho^{\prime }T^{\prime }T^{\prime }}- & c_{p^{\prime }p^{\prime }T^{\prime }}\;c_{\rho^{\prime }T^{\prime }}) & \;{C\;V}_{\;\rho^{\prime }}\;{C\;V}_{\;T^{\prime }} \end{array}$$

(20c) $$\begin{array}{cccccccccccc} c_{p^{\prime }p^{\prime }p^{\prime }\rho^{\prime }}\; & C\;||V_{p^{\prime }} & \overset{\left(13\right)}{=}\; & & c_{p^{\prime }p^{\prime }\rho^{\prime }\rho^{\prime }} & \;{C\;V}_{\;\rho^{\prime }}+ & c_{p^{\prime }p^{\prime }\rho^{\prime }T^{\prime }} & \;{C\;V}_{\;T^{\prime }}+ & & (c_{p^{\prime }p^{\prime }\rho^{\prime }\rho^{\prime }T^{\prime }}- & c_{p^{\prime }p^{\prime }\rho^{\prime }}\;c_{\rho^{\prime }T^{\prime }}) & \;{C\;V}_{\;\rho^{\prime }}\;{C\;V}_{\;T^{\prime }} \end{array}$$

(20d) $$\begin{array}{cccccccccccc} c_{p^{\prime }p^{\prime }T^{\prime }T^{\prime }}\; & C\;||V_{p^{\prime }} & \overset{\left(13\right)}{=}\; & & c_{p^{\prime }\rho^{\prime }T^{\prime }T^{\prime }} & \;{C\;V}_{\;\rho^{\prime }}+ & c_{p^{\prime }T^{\prime }T^{\prime }T^{\prime }} & \;{C\;V}_{\;T^{\prime }}+ & & (c_{p^{\prime }\rho^{\prime }T^{\prime }T^{\prime }T^{\prime }}- & c_{p^{\prime }T^{\prime }T^{\prime }}\;c_{\rho^{\prime }T^{\prime }}) & \;{C\;V}_{\;\rho^{\prime }}\;{C\;V}_{\;T^{\prime }} \end{array}$$

(20e) $$\begin{array}{cccccccccccc} c_{p^{\prime }p^{\prime }\rho^{\prime }T^{\prime }}\; & C\;||V_{p^{\prime }} & \overset{\left(13\right)}{=}\; & & c_{p^{\prime }\rho^{\prime }\rho^{\prime }T^{\prime }} & \;{C\;V}_{\;\rho^{\prime }}+ & c_{p^{\prime }\rho^{\prime }T^{\prime }T^{\prime }} & \;{C\;V}_{\;T^{\prime }}+ & & (c_{p^{\prime }\rho^{\prime }\rho^{\prime }T^{\prime }T^{\prime }}- & c_{p^{\prime }\rho^{\prime }T^{\prime }}\;c_{\rho^{\prime }T^{\prime }}) & \;{C\;V}_{\;\rho^{\prime }}\;{C\;V}_{\;T^{\prime }} \end{array}$$

(20f) $$\begin{array}{cccccccccccc} c_{p^{\prime }p^{\prime }\rho^{\prime }\rho^{\prime }}\; & C\;||V_{p^{\prime }} & \overset{\left(13\right)}{=}\; & & c_{p^{\prime }\rho^{\prime }\rho^{\prime }\rho^{\prime }} & \;{C\;V}_{\;\rho^{\prime }}+ & c_{p^{\prime }\rho^{\prime }\rho^{\prime }T^{\prime }} & \;{C\;V}_{\;T^{\prime }}+ & & (c_{p^{\prime }\rho^{\prime }\rho^{\prime }\rho^{\prime }T^{\prime }}- & c_{p^{\prime }\rho^{\prime }\rho^{\prime }}\;c_{\rho^{\prime }T^{\prime }}) & \;{C\;V}_{\;\rho^{\prime }}\;{C\;V}_{\;T^{\prime }} \end{array}$$

(20g) $$\begin{array}{cccccccccccc} c_{p^{\prime }T^{\prime }T^{\prime }T^{\prime }}\; & C\;||V_{p^{\prime }} & \overset{\left(13\right)}{=}\; & & c_{\rho^{\prime }T^{\prime }T^{\prime }T^{\prime }} & \;{C\;V}_{\;\rho^{\prime }}+ & F_{T^{\prime }} & \;{C\;V}_{\;T^{\prime }}+ & & (c_{\rho^{\prime }T^{\prime }T^{\prime }T^{\prime }T^{\prime }}- & S_{T^{\prime }}\;c_{\rho^{\prime }T^{\prime }}) & \;{C\;V}_{\;\rho^{\prime }}\;{C\;V}_{\;T^{\prime }} \end{array}$$

(20h) $$\begin{array}{cccccccccccc} c_{p^{\prime }\rho^{\prime }T^{\prime }T^{\prime }}\; & C\;||V_{p^{\prime }} & \overset{\left(13\right)}{=}\; & & c_{\rho^{\prime }\rho^{\prime }T^{\prime }T^{\prime }} & \;{C\;V}_{\;\rho^{\prime }}+ & c_{\rho^{\prime }T^{\prime }T^{\prime }T^{\prime }} & \;{C\;V}_{\;T^{\prime }}+ & & (c_{\rho^{\prime }\rho^{\prime }T^{\prime }T^{\prime }T^{\prime }}- & c_{\rho^{\prime }T^{\prime }T^{\prime }}\;c_{\rho^{\prime }T^{\prime }}) & \;{C\;V}_{\;\rho^{\prime }}\;{C\;V}_{\;T^{\prime }} \end{array}$$

(20i) $$\begin{array}{cccccccccccc} c_{p^{\prime }\rho^{\prime }\rho^{\prime }T^{\prime }}\; & C\;||V_{p^{\prime }} & \overset{\left(13\right)}{=}\; & & c_{\rho^{\prime }\rho^{\prime }\rho^{\prime }T^{\prime }} & \;{C\;V}_{\;\rho^{\prime }}+ & c_{\rho^{\prime }\rho^{\prime }T^{\prime }T^{\prime }} & \;{C\;V}_{\;T^{\prime }}+ & & (c_{\rho^{\prime }\rho^{\prime }\rho^{\prime }T^{\prime }T^{\prime }}- & c_{\rho^{\prime }\rho^{\prime }T^{\prime }}\;c_{\rho^{\prime }T^{\prime }}) & \;{C\;V}_{\;\rho^{\prime }}\;{C\;V}_{\;T^{\prime }} \end{array}$$

(20j) $$\begin{array}{cccccccccccc} c_{p^{\prime }\rho^{\prime }\rho^{\prime }\rho^{\prime }}\; & C\;||V_{p^{\prime }} & \overset{\left(13\right)}{=}\; & & F_{\rho^{\prime }} & \;{C\;V}_{\;\rho^{\prime }}+ & c_{\rho^{\prime }\rho^{\prime }\rho^{\prime }T^{\prime }} & \;{C\;V}_{\;T^{\prime }}+ & & (c_{\rho^{\prime }\rho^{\prime }\rho^{\prime }\rho^{\prime }T^{\prime }}- & S_{\rho^{\prime }}\;c_{\rho^{\prime }T^{\prime }}) & \;{C\;V}_{\;\rho^{\prime }}\;{C\;V}_{\;T^{\prime }} \end{array}$$

In this case, the nonlinear terms containing the product ${\mathrm{CV}}_{\rho^{\prime }}\;{\mathrm{CV}}_{T^{\prime }}$ on the RHS of (20) depend on differences between 5CCs and 3CCs.

The leading terms in (18)–(20), and generally for the *n*CCs in (13), are $O(C\;||V_{p^{\prime }},{C\;V}_{\;\rho^{\prime }},{C\;V}_{\;T^{\prime }})$, with the three CVs being approximately of the same order-of-magnitude The $O({C\;V}_{\;\rho^{\prime }}\;{C\;V}_{\;T^{\prime }})$ nonlinear terms on the RHS of (18)–(20) are obviously 2oTs (3), with the coefficient depending on (n+1)CCs. The nonlinearity included in $C\;||V_{p^{\prime }}$ is of a higher order (21).

### 4.4. Linearization of the Equations Between 3CCs

For sufficiently weak fluctuation amplitudes, $\max\left({C\;V}_{\;p^{\prime }},{C\;V}_{\;\rho^{\prime }},{C\;V}_{\;T^{\prime }}\right)\;\ll \;1$, the six exact equations (19) for the 3CCs can be linearized by dropping $O({C\;V}_{\;\rho^{\prime }}\;{C\;V}_{\;T^{\prime }})$ and higher-order terms. The term $C\;||V_{p^{\prime }}$ is easily linearized by replacing

(21) $$\begin{array}{c} C\;||V_{p^{\prime }}={C\;V}_{\;p^{\prime }}+\left({C\;||}_{p^{\prime }}-{C\;V}_{\;p^{\prime }}\right)\overset{\left(9\right)}{=}{C\;V}_{\;p^{\prime }}+c_{\rho^{\prime }T^{\prime }}\;{C\;V}_{\;p^{\prime }}\;{C\;V}_{\;\rho^{\prime }}\;{C\;V}_{\;T^{\prime }} \end{array}$$

on the LHS of (19) and transferring the $c_{\rho^{\prime }T^{\prime }}\;{C\;V}_{\;p^{\prime }}\;{C\;V}_{\;\rho^{\prime }}\;{C\;V}_{\;T^{\prime }}$ term to the RHS. Upon division by ${C\;V}_{\;p^{\prime }}$, (19) yields the, formally $O({C\;V}_{\;T^{\prime }})$-accurate, linearized approximations

(22a) $$\begin{array}{ccccccccc} S_{p^{\prime }} & \overset{\left(19a)\mathrm{and}\;(21\right)}{≊} & \;c_{p^{\prime }p^{\prime }\rho^{\prime }} & \;\frac{{C\;V}_{\;\rho^{\prime }}}{{C\;V}_{\;p^{\prime }}} & + & & c_{p^{\prime }p^{\prime }T^{\prime }} & \;\frac{{C\;V}_{\;T^{\prime }}}{{C\;V}_{\;p^{\prime }}} & +O\left({C\;V}_{\;T^{\prime }}\right) \end{array}$$

(22b) $$\begin{array}{ccccccccc} c_{p^{\prime }p^{\prime }T^{\prime }} & \overset{\left(19b)\mathrm{and}\;(21\right)}{≊} & \;c_{p^{\prime }\rho^{\prime }T^{\prime }} & \;\frac{{C\;V}_{\;\rho^{\prime }}}{{C\;V}_{\;p^{\prime }}} & + & & c_{p^{\prime }T^{\prime }T^{\prime }} & \;\frac{{C\;V}_{\;T^{\prime }}}{{C\;V}_{\;p^{\prime }}} & +O\left({C\;V}_{\;T^{\prime }}\right) \end{array}$$

(22c) $$\begin{array}{ccccccccc} c_{p^{\prime }p^{\prime }\rho^{\prime }} & \overset{\left(19c)\mathrm{and}\;(21\right)}{≊} & \;c_{p^{\prime }\rho^{\prime }\rho^{\prime }} & \;\frac{{C\;V}_{\;\rho^{\prime }}}{{C\;V}_{\;p^{\prime }}} & + & & c_{p^{\prime }\rho^{\prime }T^{\prime }} & \;\frac{{C\;V}_{\;T^{\prime }}}{{C\;V}_{\;p^{\prime }}} & +O\left({C\;V}_{\;T^{\prime }}\right) \end{array}$$

(22d) $$\begin{array}{ccccccccc} c_{p^{\prime }\rho^{\prime }\rho^{\prime }} & \overset{\left(19d)\mathrm{and}\;(21\right)}{≊} & \;S_{\rho^{\prime }} & \;\frac{{C\;V}_{\;\rho^{\prime }}}{{C\;V}_{\;p^{\prime }}} & + & & c_{\rho^{\prime }\rho^{\prime }T^{\prime }} & \;\frac{{C\;V}_{\;T^{\prime }}}{{C\;V}_{\;p^{\prime }}} & +O\left({C\;V}_{\;T^{\prime }}\right) \end{array}$$

(22e) $$\begin{array}{ccccccccc} c_{p^{\prime }T^{\prime }T^{\prime }} & \overset{\left(19e)\mathrm{and}\;(21\right)}{≊} & \;c_{\rho^{\prime }T^{\prime }T^{\prime }} & \;\frac{{C\;V}_{\;\rho^{\prime }}}{{C\;V}_{\;p^{\prime }}} & + & & S_{T^{\prime }} & \;\frac{{C\;V}_{\;T^{\prime }}}{{C\;V}_{\;p^{\prime }}} & +O\left({C\;V}_{\;T^{\prime }}\right) \end{array}$$

(22f) $$\begin{array}{ccccccccc} c_{p^{\prime }\rho^{\prime }T^{\prime }} & \overset{\left(19f)\mathrm{and}\;(21\right)}{≊} & \;c_{\rho^{\prime }\rho^{\prime }T^{\prime }} & \;\frac{{C\;V}_{\;\rho^{\prime }}}{{C\;V}_{\;p^{\prime }}} & + & & c_{\rho^{\prime }T^{\prime }T^{\prime }} & \;\frac{{C\;V}_{\;T^{\prime }}}{{C\;V}_{\;p^{\prime }}} & +O\left({C\;V}_{\;T^{\prime }}\right) \end{array}$$

This is a linear system of six equations between the 10 3CCs (Table 1) whose coefficients are the ratios $\left\{{C\;V}_{\;\rho^{\prime }}/{C\;V}_{\;p^{\prime }},{C\;V}_{\;T^{\prime }}/{C\;V}_{\;p^{\prime }}\right\}$ of the fluctuation amplitudes. The approximation errors in (22) are determined by (19) and (21) as

(23a) $$\begin{array}{ccccccc} & \mathrm{error}\left(S_{p^{\prime }}\right) & \overset{\left(22),(21),(19\right)}{=}\; & (c_{p^{\prime }p^{\prime }\rho^{\prime }T^{\prime }}- & c_{\rho^{\prime }T^{\prime }}) & \;\frac{{C\;V}_{\;\rho^{\prime }}}{{C\;V}_{\;p^{\prime }}}\;{C\;V}_{\;T^{\prime }}- & S_{p^{\prime }}\;c_{\rho^{\prime }T^{\prime }}\;{C\;V}_{\;\rho^{\prime }}\;{C\;V}_{\;T^{\prime }} \end{array}$$

(23b) $$\begin{array}{ccccccc} & \mathrm{error}\left(c_{p^{\prime }p^{\prime }T^{\prime }}\right) & \overset{\left(22),(21),(19\right)}{=}\; & (c_{p^{\prime }\rho^{\prime }T^{\prime }T^{\prime }}- & c_{p^{\prime }T^{\prime }}\;c_{\rho^{\prime }T^{\prime }}) & \;\frac{{C\;V}_{\;\rho^{\prime }}}{{C\;V}_{\;p^{\prime }}}\;{C\;V}_{\;T^{\prime }}- & c_{p^{\prime }p^{\prime }T^{\prime }}\;c_{\rho^{\prime }T^{\prime }}\;{C\;V}_{\;\rho^{\prime }}\;{C\;V}_{\;T^{\prime }} \end{array}$$

(23c) $$\begin{array}{ccccccc} & \mathrm{error}\left(c_{p^{\prime }p^{\prime }\rho^{\prime }}\right) & \overset{\left(22),(21),(19\right)}{=}\; & (c_{p^{\prime }\rho^{\prime }\rho^{\prime }T^{\prime }}- & c_{p^{\prime }\rho^{\prime }}\;c_{\rho^{\prime }T^{\prime }}) & \;\frac{{C\;V}_{\;\rho^{\prime }}}{{C\;V}_{\;p^{\prime }}}\;{C\;V}_{\;T^{\prime }}- & c_{p^{\prime }p^{\prime }\rho^{\prime }}\;c_{\rho^{\prime }T^{\prime }}\;{C\;V}_{\;\rho^{\prime }}\;{C\;V}_{\;T^{\prime }} \end{array}$$

(23d) $$\begin{array}{ccccccc} & \mathrm{error}\left(c_{p^{\prime }\rho^{\prime }\rho^{\prime }}\right) & \overset{\left(22),(21),(19\right)}{=}\; & (c_{\rho^{\prime }\rho^{\prime }\rho^{\prime }T^{\prime }}- & c_{\rho^{\prime }T^{\prime }}) & \;\frac{{C\;V}_{\;\rho^{\prime }}}{{C\;V}_{\;p^{\prime }}}\;{C\;V}_{\;T^{\prime }}- & c_{p^{\prime }\rho^{\prime }\rho^{\prime }}\;c_{\rho^{\prime }T^{\prime }}\;{C\;V}_{\;\rho^{\prime }}\;{C\;V}_{\;T^{\prime }} \end{array}$$

(23e) $$\begin{array}{ccccccc} & \mathrm{error}\left(c_{p^{\prime }T^{\prime }T^{\prime }}\right) & \overset{\left(22),(21),(19\right)}{=}\; & (c_{\rho^{\prime }T^{\prime }T^{\prime }T^{\prime }}- & c_{\rho^{\prime }T^{\prime }}) & \;\frac{{C\;V}_{\;\rho^{\prime }}}{{C\;V}_{\;p^{\prime }}}\;{C\;V}_{\;T^{\prime }}- & c_{p^{\prime }T^{\prime }T^{\prime }}\;c_{\rho^{\prime }T^{\prime }}\;{C\;V}_{\;\rho^{\prime }}\;{C\;V}_{\;T^{\prime }} \end{array}$$

(23f) $$\begin{array}{ccccccc} & \mathrm{error}\left(c_{p^{\prime }\rho^{\prime }T^{\prime }}\right) & \overset{\left(22),(21),(19\right)}{=}\; & (c_{\rho^{\prime }\rho^{\prime }T^{\prime }T^{\prime }}- & c_{\rho^{\prime }T^{\prime }}^{2}) & \;\frac{{C\;V}_{\;\rho^{\prime }}}{{C\;V}_{\;p^{\prime }}}\;{C\;V}_{\;T^{\prime }}- & c_{p^{\prime }\rho^{\prime }T^{\prime }}\;c_{\rho^{\prime }T^{\prime }}\;{C\;V}_{\;\rho^{\prime }}\;{C\;V}_{\;T^{\prime }} \end{array}$$

with a leading $O({C\;V}_{\;T^{\prime }})$ term and a higher $O({C\;V}_{\;\rho^{\prime }}\;{C\;V}_{\;T^{\prime }})$ term whose origin is (21). This higher $O({C\;V}_{\;\rho^{\prime }}\;{C\;V}_{\;T^{\prime }})$ term is proportional to the 3CC that is approximated (the corresponding approximation error is implicit) and was found to be negligibly small compared to the leading $O({C\;V}_{\;T^{\prime }})$ terms in (23) for all available TPC flow data [12]. In compressible TPC flows, the ${C\;V}_{\;T^{\prime }}$-peak in the buffer zone increases roughly with ${\bar{M}}_{{\mathrm{CL}}_{x}}^{2}$ (Figure 1c). Therefore, the linearized approximations (22) are expected to lose accuracy with an increasing ${\bar{M}}_{{\mathrm{CL}}_{x}}$.

**Figure 4:**
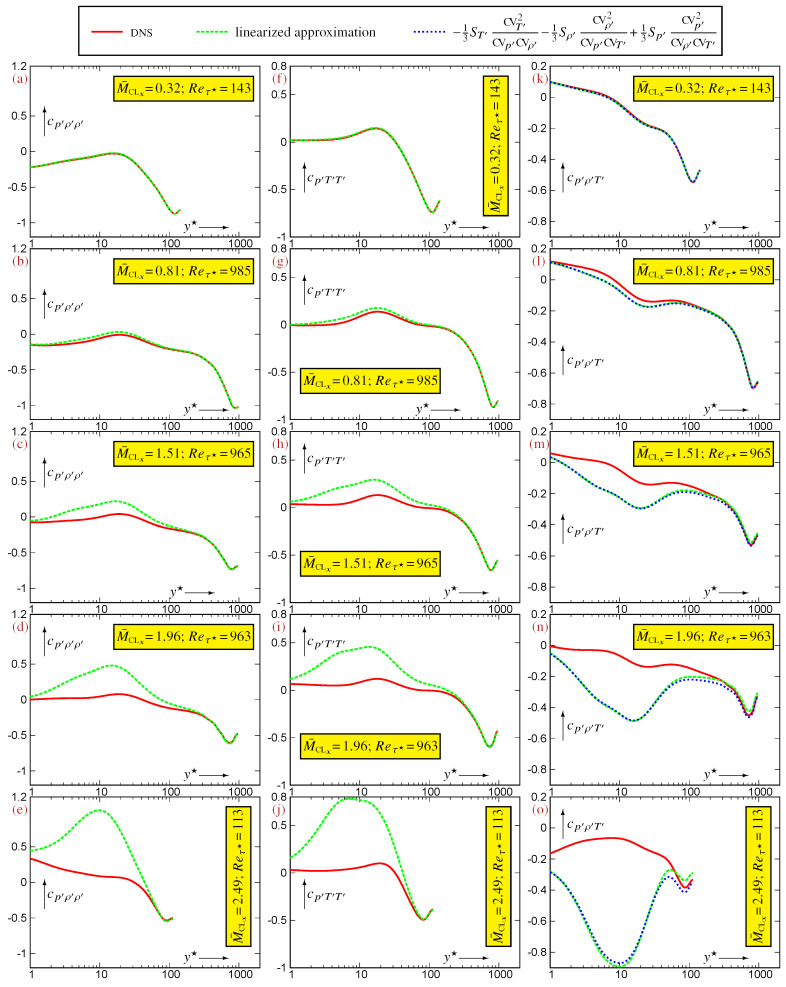
Assessment of the approximate linearized expressions (22d)–(22f) for the 3CCs $\left\{c_{p^{\prime }\rho^{\prime }\rho^{\prime }}\;\left(a–e\right),c_{p^{\prime }T^{\prime }T^{\prime }}\;\left(f–j\right),c_{p^{\prime }\rho^{\prime }T^{\prime }}\;\left(k–o\right)\right\}$ and also of (26) for $c_{p^{\prime }\rho^{\prime }T^{\prime }}\;\left(k–o\right)$, evaluated using DNS data [12] for compressible turbulent plane channel flows in the range ${Re}_{\tau^{★}}\in \left[113,985\right]$ and ${\bar{M}}_{{\mathrm{CL}}_{x}}\in \left[0.32,2.49\right]$, plotted against the HCB-scaled nondimensional wall-distance $y^{★}$ (logscale).

An indication of the flow’s Mach number beyond which the linearized approximation is no longer accurate is obtained by assessing the linearized expressions (22) for the 3CCs using DNS data for representative compressible TPC flows (Figure 3 and Figure 4). This limit ${\bar{M}}_{{\mathrm{CL}}_{x}}$ depends on the specific correlation that is considered. Approximations (22a)–(22c), expressing the 3CCs $\left\{S_{p^{\prime }},c_{p^{\prime }p^{\prime }T^{\prime }},c_{p^{\prime }p^{\prime }\rho^{\prime }}\right\}$, are very robust and remain very accurate up to the highest available ${\bar{M}}_{{\mathrm{CL}}_{x}}\;=\;2.49$ (Figure 3). On the contrary, approximations (22d)–(22f) expressing the 3CCs $\left\{c_{p^{\prime }\rho^{\prime }\rho^{\prime }},c_{p^{\prime }T^{\prime }T^{\prime }},c_{p^{\prime }\rho^{\prime }T^{\prime }}\right\}$ are less robust (Figure 4). The excellent accuracy observed at ${\bar{M}}_{{\mathrm{CL}}_{x}}\;=\;0.32$ (Figure 4a,f,k) remains satisfactory at ${\bar{M}}_{{\mathrm{CL}}_{x}}\;=\;0.81$ (Figure 4b,g,l), but noticeable discrepancies appear already at ${\bar{M}}_{{\mathrm{CL}}_{x}}\;=\;1.51$ (Figure 4c,h,m), in the buffer region ($5\;⪅\;y^{★}\;⪅\;40$). These discrepancies grow with increasing ${\bar{M}}_{{\mathrm{CL}}_{x}}$ and become unacceptable at ${\bar{M}}_{{\mathrm{CL}}_{x}}\;=\;1.96$ (Figure 4d,i,n) and ${\bar{M}}_{{\mathrm{CL}}_{x}}\;=\;2.49$ (Figure 4e,j,o). Hence, for compressible TPC flows, the system (23) remains an accurate approximation of the system of exact relations (19) only for ${\bar{M}}_{{\mathrm{CL}}_{x}}\;⪅\;1.5$. This ${\bar{M}}_{{\mathrm{CL}}_{x}}\;⪅\;1.5$ limit is more stringent than the corresponding ${\bar{M}}_{{\mathrm{CL}}_{x}}\;⪅\;2.0$ limit for 2CCs [4], for which a similar accuracy is obtained for the linearization of system (18), suggesting that the nonlinear effects associated with the product ${\left(\rho^{\prime }\;T^{\prime }\right)}^{\prime }$ in the fluctuating EoS (7) are stronger in relations between 3CCs compared to relations between 2CCs.

An intuitive explanation of the difference in accuracy with increasing ${\bar{M}}_{{\mathrm{CL}}_{x}}$ between the robust approximations (22a)–(22c) for $\left\{S_{p^{\prime }},c_{p^{\prime }p^{\prime }T^{\prime }},c_{p^{\prime }p^{\prime }\rho^{\prime }}\right\}$ and the fragile approximations (22d)–(22f) for $\left\{c_{p^{\prime }\rho^{\prime }\rho^{\prime }},c_{p^{\prime }T^{\prime }T^{\prime }},c_{p^{\prime }\rho^{\prime }T^{\prime }}\right\}$ is that the former express 3CCs containing $p^{\prime }$ in terms of 3CCs also containing $p^{\prime }$, whereas the latter express 3CCs containing $p^{\prime }$ in terms of $\left(\rho^{\prime }T^{\prime }\right)$-statistics. Exact expressions for $\bar{p^{\prime n}}$ in terms of $\left(\rho^{\prime },T^{\prime }\right)$-statistics (15) involve higher 2n-order correlations, e.g., (17) for $S_{p^{\prime }}$ involves 6-order moments. However, this explanation, although plausible, remains conjectural.

**Figure 5:**
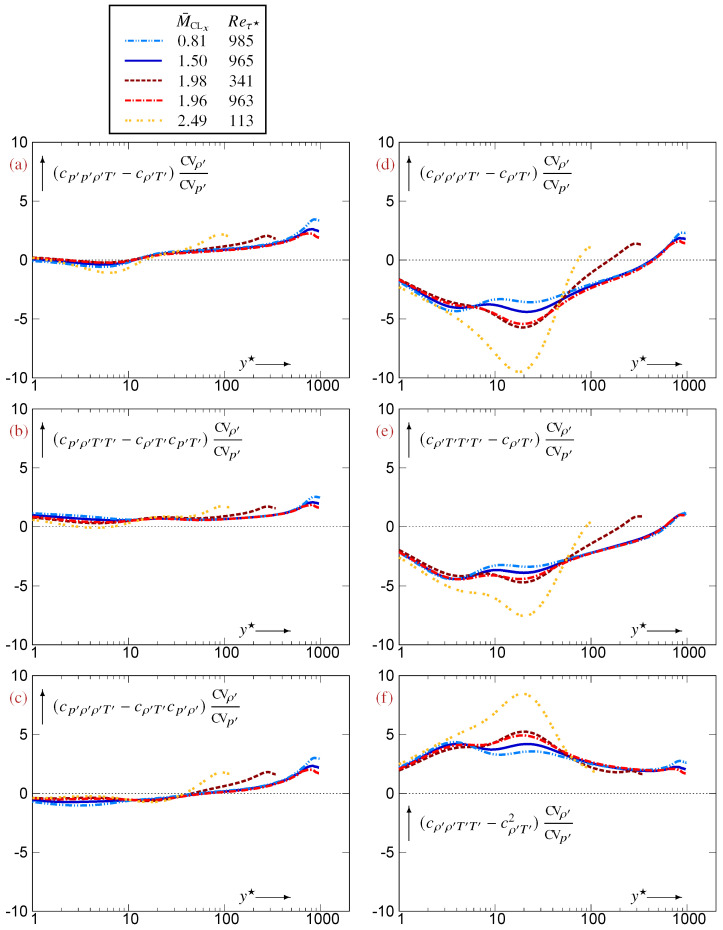
Coefficient multiplying ${C\;V}_{\;T^{\prime }}$ in the expression for the leading-order term of the linearization error (23) in the approximate expressions (22) for the 3CCs, $S_{p^{\prime }}$ (**a**), $c_{p^{\prime }p^{\prime }T^{\prime }}$ (**b**), $c_{p^{\prime }p^{\prime }\rho^{\prime }}$ (**c**), $c_{p^{\prime }\rho^{\prime }\rho^{\prime }}$ (**d**), $c_{p^{\prime }T^{\prime }T^{\prime }}$ (**e**) and $c_{p^{\prime }\rho^{\prime }T^{\prime }}$ (**f**), evaluated using DNS data [12] for compressible turbulent plane channel flows in the range ${Re}_{\tau^{★}}\in \left[113,985\right]$ and ${\bar{M}}_{{\mathrm{CL}}_{x}}\in \left[0.81,2.49\right]$, plotted against the HCB-scaled nondimensional wall-distance $y^{★}$ (logscale).

A quantitative assessment is obtained by considering (Figure 5) the coefficients multiplying ${C\;V}_{\;T^{\prime }}$ in the leading-order term of the approximation error (23) for each of the linearized relations (22). Invariably, these coefficients contain the ratio ${C\;V}_{\;\rho^{\prime }}/{C\;V}_{\;p^{\prime }}$ which reaches values ∈[2,4] in the buffer region ([4], Figure 3, p. 458), where the peak of ${C\;V}_{\;\rho^{\prime }}$ occurs (Figure 1a), whereas ${C\;V}_{\;p^{\prime }}$ has a much lower inner peak (Figure 1b). Therefore, differences in the increase in the approximation error of the linearized relations (22) with increasing ${\bar{M}}_{{\mathrm{CL}}_{x}}$ (increasing ${C\;V}_{\;T^{\prime }}$) are essentially controlled by the 4CCs in the leading-order term of the approximation error (23).

The DNS data for compressible TPC flows indicate that in the buffer region ($5\;⪅\;y^{★}\;⪅\;40$), the coefficients of ${C\;V}_{\;T^{\prime }}$ in the leading-order term of the approximation error (23a)–(23c) for the 3CCs $\left\{S_{p^{\prime }},c_{p^{\prime }p^{\prime }T^{\prime }},c_{p^{\prime }p^{\prime }\rho^{\prime }}\right\}$, for which the linearized approximation is robust (Figure 3), are small (Figure 5a–c), <1 in absolute value. On the contrary, for the 3CCs $\left\{c_{p^{\prime }\rho^{\prime }\rho^{\prime }},c_{p^{\prime }T^{\prime }T^{\prime }},c_{p^{\prime }\rho^{\prime }T^{\prime }}\right\}$, for which the linearized approximation is fragile with increasing ${\bar{M}}_{{\mathrm{CL}}_{x}}$ (Figure 4), the coefficients of ${C\;V}_{\;T^{\prime }}$ in the leading-order term of the approximation error (23d)–(23f) are large (Figure 5d–f), ∼5 in absolute value. Furthermore, their absolute value increases with increasing ${\bar{M}}_{{\mathrm{CL}}_{x}}$ to reach ∼10 for ${\bar{M}}_{{\mathrm{CL}}_{x}}\;=\;2.49$ (Figure 5d–f). Further analysis requires the determination of bounds for the quadruple correlation coefficients (4CCs), which are studied in Section 5.

### 4.5. Linearized Relations Between 3CCs

Despite the rather low $M_{{\mathrm{CL}}_{x}}\;⪅\;1.5$ limit of validity of the linearized approximations for 3CCs, the study of the linear system (22) provides insight into the interdependencies that the fluctuating EoS (7) imposes on the 3CCs.

There are 3+3−13=10 combinations with repetitions ([31], p. 744) of the fluctuations of the three basic thermodynamic variables $\left\{p^{\prime },\rho^{\prime },T^{\prime }\right\}$ into distinct triple correlations $\{\bar{p^{\prime }\rho^{\prime }T^{\prime }}$, $\bar{p^{\prime }p^{\prime }\rho^{\prime }}$, $\bar{p^{\prime }p^{\prime }T^{\prime }}$, $\bar{p^{\prime 3}}$, $\bar{p^{\prime }\rho^{\prime }\rho^{\prime }}$, $\bar{\rho^{\prime }\rho^{\prime }T^{\prime }}$, $\bar{T^{\prime 3}}$, $\bar{p^{\prime }T^{\prime }T^{\prime }}$, $\bar{\rho^{\prime }T^{\prime }T^{\prime }}$, $\bar{\rho^{\prime 3}}\}$, and only six linear equations (22) are obtained by truncating (19) to $O({C\;V}_{\;p^{\prime }},{C\;V}_{\;\rho^{\prime }},{C\;V}_{\;T^{\prime }})$. We can therefore approximate 6 of the 10 3CCs in terms of 4 linearly independent ones (it is assumed that the coefficients of variation $\left\{{C\;V}_{\;p^{\prime }},{C\;V}_{\;\rho^{\prime }},{C\;V}_{\;T^{\prime }}\right\}$ are known).

The last three relations (22d)–(22f) express $\left\{c_{p^{\prime }\rho^{\prime }\rho^{\prime }},c_{p^{\prime }T^{\prime }T^{\prime }},c_{p^{\prime }\rho^{\prime }T^{\prime }}\right\}$ in terms of $\left(\rho^{\prime },T^{\prime }\right)$-statistics only. The higher-order $O({C\;V}_{\;\rho^{\prime }}\;{C\;V}_{\;T^{\prime }})$ terms that were dropped from (19d)–(19f) to obtain (22d)–(22f) are also $\left(\rho^{\prime },T^{\prime }\right)$-statistics. The system (22) can be solved for all third-order correlations containing $p^{\prime }$, $\left\{S_{p^{\prime }},c_{p^{\prime }p^{\prime }\rho^{\prime }},c_{p^{\prime }p^{\prime }T^{\prime }},c_{p^{\prime }\rho^{\prime }\rho^{\prime }},c_{p^{\prime }T^{\prime }T^{\prime }},c_{p^{\prime }\rho^{\prime }T^{\prime }}\right\}$, thus replacing (19a)–(19c) with

(24a) $$\begin{array}{cccccccccc} S_{p^{\prime }} & \overset{\left(22\right)}{≊} & \;3\;c_{\rho^{\prime }\rho^{\prime }T^{\prime }} & \;\frac{{C\;V}_{\;\rho^{\prime }}^{2}\;{C\;V}_{\;T^{\prime }}}{{C\;V}_{\;p^{\prime }}^{3}} & + & & \;3\;c_{\rho^{\prime }T^{\prime }T^{\prime }} & \;\frac{{C\;V}_{\;\rho^{\prime }}\;{C\;V}_{\;T^{\prime }}^{2}}{{C\;V}_{\;p^{\prime }}^{3}} & + & S_{\rho^{\prime }}\;\frac{{C\;V}_{\;\rho^{\prime }}^{3}}{{C\;V}_{\;p^{\prime }}^{3}}+S_{T^{\prime }}\;\frac{{C\;V}_{\;T^{\prime }}^{3}}{{C\;V}_{\;p^{\prime }}^{3}}+\mathrm{HoTs} \end{array}$$

(24b) $$\begin{array}{cccccccccc} c_{p^{\prime }p^{\prime }\rho^{\prime }} & \overset{\left(22\right)}{≊} & \;2\;c_{\rho^{\prime }\rho^{\prime }T^{\prime }} & \;\frac{{C\;V}_{\;\rho^{\prime }}\;{C\;V}_{\;T^{\prime }}}{{C\;V}_{\;p^{\prime }}^{2}} & + & & \;c_{\rho^{\prime }T^{\prime }T^{\prime }} & \;\frac{{C\;V}_{\;T^{\prime }}^{2}}{{C\;V}_{\;p^{\prime }}^{2}} & + & S_{\rho^{\prime }}\;\frac{{C\;V}_{\;\rho^{\prime }}^{2}}{{C\;V}_{\;p^{\prime }}^{2}}+\mathrm{HoTs} \end{array}$$

(24c) $$\begin{array}{cccccccccc} c_{p^{\prime }p^{\prime }T^{\prime }} & \overset{\left(22\right)}{≊} & \;c_{\rho^{\prime }\rho^{\prime }T^{\prime }} & \;\frac{{C\;V}_{\;\rho^{\prime }}^{2}}{{C\;V}_{\;p^{\prime }}^{2}} & + & & \;2\;c_{\rho^{\prime }T^{\prime }T^{\prime }} & \;\frac{{C\;V}_{\;\rho^{\prime }}\;{C\;V}_{\;T^{\prime }}}{{C\;V}_{\;p^{\prime }}^{2}} & + & S_{T^{\prime }}\;\frac{{C\;V}_{\;T^{\prime }}^{2}}{{C\;V}_{\;p^{\prime }}^{2}}+\mathrm{HoTs} \end{array}$$

The approximate linearized expression (24a) for the skewness $S_{p^{\prime }}$ corresponds to the leading terms (first line) of the exact expression (17) for $S_{p^{\prime }}$ in terms of $\left(\rho^{\prime },T^{\prime }\right)$-statistics which contains up to 6oTs (6-order terms) involving 6CCs, highlighting the fact that all of these extra terms in the exact expression represent effects dependent on the fluctuation intensity.

The system consisting of the three relations (24) and of the three relations (22d)–(22f) expresses $\left\{S_{p^{\prime }},c_{p^{\prime }p^{\prime }\rho^{\prime }},c_{p^{\prime }p^{\prime }T^{\prime }},c_{p^{\prime }\rho^{\prime }\rho^{\prime }},c_{p^{\prime }T^{\prime }T^{\prime }},c_{p^{\prime }\rho^{\prime }T^{\prime }}\right\}$ in terms of the four independent variables $\left\{S_{\rho^{\prime }},S_{T^{\prime }},c_{\rho^{\prime }\rho^{\prime }T^{\prime }},c_{\rho^{\prime }T^{\prime }T^{\prime }}\right\}$, in line with the conceptual approach of expressing all correlations in terms of $\left(\rho^{\prime },T^{\prime }\right)$-statistics (Section 2.4). More generally, there are ([31], p. 744) 2+n−1n=n+1 bivariate $\left(\rho^{\prime },T^{\prime }\right)$ *n*-moments, constituting a linearly independent (n+1)-tuple (Table 1).

However, not all 4-tuples of 3CCs are linearly independent. In particular, $c_{p^{\prime }\rho^{\prime }T^{\prime }}$ is linearly dependent on several symmetric combinations of the other 3CCs. Solving each of the relations (22f), (22b), (22c) for $c_{p^{\prime }\rho^{\prime }T^{\prime }}$ yields

(25a) $$\begin{array}{cc} c_{p^{\prime }\rho^{\prime }T^{\prime }}\overset{\left(22f\right)}{≊} & \;c_{\rho^{\prime }\rho^{\prime }T^{\prime }}\;\frac{{\mathrm{CV}}_{\rho^{\prime }}}{{\mathrm{CV}}_{p^{\prime }}}\;+\;c_{\rho^{\prime }T^{\prime }T^{\prime }}\;\frac{{\mathrm{CV}}_{T^{\prime }}}{{\mathrm{CV}}_{p^{\prime }}}\;+\overset{∼\frac{{\mathrm{CV}}_{\rho^{\prime }}}{{\mathrm{CV}}_{p^{\prime }}}\;O\left({\mathrm{CV}}_{T^{\prime }}\right)}{\overset{︷}{O\left(\frac{{\mathrm{CV}}_{\rho^{\prime }}\;{\mathrm{CV}}_{T^{\prime }}}{{\mathrm{CV}}_{p^{\prime }}}\right)}} \end{array}$$

(25b) $$\begin{array}{cc} \overset{\left(22b\right)}{≊} & \;c_{p^{\prime }p^{\prime }T^{\prime }}\;\frac{{\mathrm{CV}}_{p^{\prime }}}{{\mathrm{CV}}_{\rho^{\prime }}}\;-\;c_{p^{\prime }T^{\prime }T^{\prime }}\;\frac{{\mathrm{CV}}_{T^{\prime }}}{{\mathrm{CV}}_{\rho^{\prime }}}\;+O\left({\mathrm{CV}}_{T^{\prime }}\right) \end{array}$$

(25c) $$\begin{array}{cc} \overset{\left(22c\right)}{≊} & \;c_{p^{\prime }p^{\prime }\rho^{\prime }}\;\frac{{\mathrm{CV}}_{p^{\prime }}}{{\mathrm{CV}}_{T^{\prime }}}\;-\;c_{p^{\prime }\rho^{\prime }\rho^{\prime }}\;\frac{{\mathrm{CV}}_{\rho^{\prime }}}{{\mathrm{CV}}_{T^{\prime }}}\;+O\left({\mathrm{CV}}_{\rho^{\prime }}\right) \end{array}$$

Combining (24a) and (25a), we have

(26) $$\begin{array}{c} c_{p^{\prime }\rho^{\prime }T^{\prime }}\overset{\left(22),(24a),(25a\right)}{≊}\frac{\frac{1}{3}S_{p^{\prime }}\;{C\;V}_{\;p^{\prime }}^{3}-\frac{1}{3}S_{\rho^{\prime }}\;{C\;V}_{\;\rho^{\prime }}^{3}-\frac{1}{3}S_{T^{\prime }}\;{C\;V}_{\;T^{\prime }}^{3}}{{C\;V}_{\;p^{\prime }}\;{C\;V}_{\;\rho^{\prime }}\;{C\;V}_{\;T^{\prime }}}+O\left({C\;V}_{\;p^{\prime }},{C\;V}_{\;\rho^{\prime }},{C\;V}_{\;T^{\prime }},\frac{{C\;V}_{\;\rho^{\prime }}^{2}}{{C\;V}_{\;p^{\prime }}},\frac{{C\;V}_{\;T^{\prime }}^{2}}{{C\;V}_{\;p^{\prime }}},\frac{{C\;V}_{\;\rho^{\prime }}{C\;V}_{\;T^{\prime }}}{{C\;V}_{\;p^{\prime }}}\right) \end{array}$$

where the order of magnitude of the approximation error is obtained by including in the calculations the orders of magnitude of the approximation errors in (22). The accuracy of (26) with an increasing fluctuation amplitude is similar to the accuracy of (22f), which approximates $c_{p^{\prime }\rho^{\prime }T^{\prime }}$ with $\left(\rho^{\prime },T^{\prime }\right)$-statistics (Figure 4k–n). According to (26), $\left\{c_{p^{\prime }\rho^{\prime }T^{\prime }},S_{p^{\prime }},S_{\rho^{\prime }},S_{T^{\prime }}\right\}$ are not linearly independent because at the weakly compressible limit, the individual skewnesses and CVs determine the triple correlation coefficient $c_{p^{\prime }\rho^{\prime }T^{\prime }}$. Therefore, to express the 3CCs in terms of the individual skewnesses, we cannot choose $c_{p^{\prime }\rho^{\prime }T^{\prime }}$ as the fourth independent variable. Choosing, e.g., as independent variables $\left\{c_{\rho^{\prime }\rho^{\prime }T^{\prime }},S_{p^{\prime }},S_{\rho^{\prime }},S_{T^{\prime }}\right\}$ and solving the system (22) for $\left\{c_{p^{\prime }\rho^{\prime }T^{\prime }},c_{p^{\prime }p^{\prime }\rho^{\prime }},c_{p^{\prime }\rho^{\prime }\rho^{\prime }},c_{p^{\prime }p^{\prime }T^{\prime }},c_{p^{\prime }T^{\prime }T^{\prime }},c_{\rho^{\prime }T^{\prime }T^{\prime }}\right\}$ yields (26) and five other equations:

(27a) $$\begin{array}{cc} c_{p^{\prime }p^{\prime }\rho^{\prime }}\;{C\;V}_{\;p^{\prime }}^{2}\;{C\;V}_{\;\rho^{\prime }}\overset{\left(22\right)}{≊} & +c_{\rho^{\prime }\rho^{\prime }T^{\prime }}\;{C\;V}_{\;\rho^{\prime }}^{2}\;{C\;V}_{\;T^{\prime }}+\frac{1}{3}S_{p^{\prime }}\;{C\;V}_{\;p^{\prime }}^{3}+\frac{2}{3}S_{\rho^{\prime }}\;{C\;V}_{\;\rho^{\prime }}^{3}-\frac{1}{3}S_{T^{\prime }}\;{C\;V}_{\;T^{\prime }}^{3}+\mathrm{HoTs} \end{array}$$

(27b) $$\begin{array}{cc} c_{p^{\prime }\rho^{\prime }\rho^{\prime }}\;{C\;V}_{\;p^{\prime }}\;{C\;V}_{\;\rho^{\prime }}^{2}\overset{\left(22\right)}{≊} & +c_{\rho^{\prime }\rho^{\prime }T^{\prime }}\;{C\;V}_{\;\rho^{\prime }}^{2}\;{C\;V}_{\;T^{\prime }}+S_{\rho^{\prime }}\;{C\;V}_{\;\rho^{\prime }}^{3}+\mathrm{HoTs}\Leftrightarrow \left(22d\right) \end{array}$$

(27c) $$\begin{array}{cc} c_{p^{\prime }p^{\prime }T^{\prime }}\;{C\;V}_{\;p^{\prime }}^{2}\;{C\;V}_{\;T^{\prime }}\overset{\left(22\right)}{≊} & -c_{\rho^{\prime }\rho^{\prime }T^{\prime }}\;{C\;V}_{\;\rho^{\prime }}^{2}\;{C\;V}_{\;T^{\prime }}+\frac{2}{3}S_{p^{\prime }}\;{C\;V}_{\;p^{\prime }}^{3}-\frac{2}{3}S_{\rho^{\prime }}\;{C\;V}_{\;\rho^{\prime }}^{3}+\frac{1}{3}S_{T^{\prime }}\;{C\;V}_{\;T^{\prime }}^{3}+\mathrm{HoTs} \end{array}$$

(27d) $$\begin{array}{cc} c_{p^{\prime }T^{\prime }T^{\prime }}\;{C\;V}_{\;p^{\prime }}\;{C\;V}_{\;T^{\prime }}^{2}\overset{\left(22\right)}{≊} & -c_{\rho^{\prime }\rho^{\prime }T^{\prime }}\;{C\;V}_{\;\rho^{\prime }}^{2}\;{C\;V}_{\;T^{\prime }}+\frac{1}{3}S_{p^{\prime }}\;{C\;V}_{\;p^{\prime }}^{3}-\frac{1}{3}S_{\rho^{\prime }}\;{C\;V}_{\;\rho^{\prime }}^{3}+\frac{2}{3}S_{T^{\prime }}\;{C\;V}_{\;T^{\prime }}^{3}+\mathrm{HoTs} \end{array}$$

(27e) $$\begin{array}{cc} c_{\rho^{\prime }T^{\prime }T^{\prime }}\;{C\;V}_{\;\rho^{\prime }}\;{C\;V}_{\;T^{\prime }}^{2}\overset{\left(22\right)}{≊} & -c_{\rho^{\prime }\rho^{\prime }T^{\prime }}\;{C\;V}_{\;\rho^{\prime }}^{2}\;{C\;V}_{\;T^{\prime }}+\frac{1}{3}S_{p^{\prime }}\;{C\;V}_{\;p^{\prime }}^{3}-\frac{1}{3}S_{\rho^{\prime }}\;{C\;V}_{\;\rho^{\prime }}^{3}-\frac{1}{3}S_{T^{\prime }}\;{C\;V}_{\;T^{\prime }}^{3}+\mathrm{HoTs}\overset{\left(24a\right)}{\Leftrightarrow }\left(22f\right) \end{array}$$

Alternatively, choosing $\left\{c_{\rho^{\prime }T^{\prime }T^{\prime }},S_{p^{\prime }},S_{\rho^{\prime }},S_{T^{\prime }}\right\}$ as independent variables and solving the system (22) for $\left\{c_{p^{\prime }\rho^{\prime }T^{\prime }},c_{p^{\prime }p^{\prime }\rho^{\prime }},c_{p^{\prime }\rho^{\prime }\rho^{\prime }},c_{p^{\prime }p^{\prime }T^{\prime }},c_{p^{\prime }T^{\prime }T^{\prime }},c_{\rho^{\prime }\rho^{\prime }T^{\prime }}\right\}$ yields (26) and five other equations:

(28a) $$\begin{array}{cc} c_{p^{\prime }p^{\prime }\rho^{\prime }}\;{C\;V}_{\;p^{\prime }}^{2}\;{C\;V}_{\;\rho^{\prime }}\overset{\left(22\right)}{≊} & -c_{\rho^{\prime }T^{\prime }T^{\prime }}\;{C\;V}_{\;\rho^{\prime }}\;{C\;V}_{\;T^{\prime }}^{2}+\frac{2}{3}S_{p^{\prime }}\;{C\;V}_{\;p^{\prime }}^{3}+\frac{1}{3}S_{\rho^{\prime }}\;{C\;V}_{\;\rho^{\prime }}^{3}-\frac{2}{3}S_{T^{\prime }}\;{C\;V}_{\;T^{\prime }}^{3}+\mathrm{HoTs} \end{array}$$

(28b) $$\begin{array}{cc} c_{p^{\prime }\rho^{\prime }\rho^{\prime }}\;{C\;V}_{\;p^{\prime }}\;{C\;V}_{\;\rho^{\prime }}^{2}\overset{\left(22\right)}{≊} & -c_{\rho^{\prime }T^{\prime }T^{\prime }}\;{C\;V}_{\;\rho^{\prime }}\;{C\;V}_{\;T^{\prime }}^{2}+\frac{1}{3}S_{p^{\prime }}\;{C\;V}_{\;p^{\prime }}^{3}+\frac{2}{3}S_{\rho^{\prime }}\;{C\;V}_{\;\rho^{\prime }}^{3}-\frac{1}{3}S_{T^{\prime }}\;{C\;V}_{\;T^{\prime }}^{3}+\mathrm{HoTs} \end{array}$$

(28c) $$\begin{array}{cc} c_{p^{\prime }p^{\prime }T^{\prime }}\;{C\;V}_{\;p^{\prime }}^{2}\;{C\;V}_{\;T^{\prime }}\overset{\left(22\right)}{≊} & +c_{\rho^{\prime }T^{\prime }T^{\prime }}\;{C\;V}_{\;\rho^{\prime }}\;{C\;V}_{\;T^{\prime }}^{2}+\frac{1}{3}S_{p^{\prime }}\;{C\;V}_{\;p^{\prime }}^{3}-\frac{1}{3}S_{\rho^{\prime }}\;{C\;V}_{\;\rho^{\prime }}^{3}+\frac{2}{3}S_{T^{\prime }}\;{C\;V}_{\;T^{\prime }}^{3}+\mathrm{HoTs} \end{array}$$

(28d) $$\begin{array}{cc} c_{p^{\prime }T^{\prime }T^{\prime }}\;{C\;V}_{\;p^{\prime }}\;{C\;V}_{\;T^{\prime }}^{2}\overset{\left(22\right)}{≊} & +c_{\rho^{\prime }T^{\prime }T^{\prime }}\;{C\;V}_{\;\rho^{\prime }}\;{C\;V}_{\;T^{\prime }}^{2}+S_{T^{\prime }}\;{C\;V}_{\;T^{\prime }}^{3}+\mathrm{HoTs}\Leftrightarrow \left(22e\right) \end{array}$$

(28e) $$\begin{array}{cc} c_{\rho^{\prime }\rho^{\prime }T^{\prime }}\;{C\;V}_{\;\rho^{\prime }}^{2}\;{C\;V}_{\;T^{\prime }}\overset{\left(22\right)}{≊} & -c_{\rho^{\prime }T^{\prime }T^{\prime }}\;{C\;V}_{\;\rho^{\prime }}\;{C\;V}_{\;T^{\prime }}^{2}+\frac{1}{3}S_{p^{\prime }}\;{C\;V}_{\;p^{\prime }}^{3}-\frac{1}{3}S_{\rho^{\prime }}\;{C\;V}_{\;\rho^{\prime }}^{3}-\frac{1}{3}S_{T^{\prime }}\;{C\;V}_{\;T^{\prime }}^{3}+\mathrm{HoTs}\Leftrightarrow \left(27e\right) \end{array}$$

Notice that there are 104=210 possible 4-tuples that can be formed from the 10 3CCs (combinations without repetitions ([31], p. 744)), but it is outside the scope of the paper to systematically investigate which 4-tuples are linearly independent. The approximate equations derived in this subsection (24)–(28) are useful for analyzing the DNS results for the triple correlations (Section 6).

## 5. Statistical Identities and Bounds

Contrary to the relations developed in the previous sections, which are consequences of the fluctuating EoS (7), we consider here general identities for third-order and fourth-order correlation coefficients between the fluctuations in three random variables $\left\{a^{\prime },b^{\prime },c^{\prime }\right\}$. These identities can be readily combined with the Schwarz inequality to determine bounds for the 3CCs.

Lumley [20] remarks that the general bounds obtained by the Schwarz inequality are not very restrictive. Progress to determining sharper bounds has been made by several authors [22]. Ogasawara’s lemma ([22], Lemma 1, p. 13) provides such slightly sharper bounds, valid for general pdfs, as a multidimensional extension of Pearson’s inequality [35]. This approach considers Schwarz’s inequality for fluctuations in functions (products) of the fluctuating variables, formalizing and systematically extending earlier approaches ([36], (2), p. 477).

We reinterpret here Ogasawara’s lemma ([22], Lemma 1, p. 13) in terms of the Schwarz inequality for the correlation coefficient

(29) $$\begin{array}{c} c_{{\left(\cdot \right)}^{\prime }{\left[\cdot \right]}^{\prime }}:=\frac{\bar{{\left(\cdot \right)}^{\prime }{\left[\cdot \right]}^{\prime }}}{{\left(\cdot \right)}_{\mathrm{rms}}^{\prime }\;{\left[\cdot \right]}_{\mathrm{rms}}^{\prime }}\;;\;c_{{\left(\cdot \right)}^{\prime }{\left[\cdot \right]}^{\prime }}^{2}\leq 1 \end{array}$$

written in the formalism of Reynolds averages and fluctuations $\left(\cdot \right)=\bar{\left(\cdot \right)}+{\left(\cdot \right)}^{\prime }$.

Functions (products) of fluctuations can be separated into an average and a fluctuating part:

(30a) $$\begin{array}{cccccccccccccc} {\left(a^{\prime }b^{\prime }\right)}^{\prime } & := & a^{\prime }b^{\prime }\;-\;\bar{a^{\prime }b^{\prime }}\; & \Rightarrow & \;\bar{{\left(a^{\prime }b^{\prime }\right)}^{\prime 2}} & = & & \bar{{\left(a^{\prime }b^{\prime }\;-\;\bar{a^{\prime }b^{\prime }}\right)}^{2}} & = & \bar{a^{\prime 2}b^{\prime 2}}\;-\;{\bar{a^{\prime }b^{\prime }}}^{2}\; & \overset{\left(2\right)}{\Rightarrow } & \;{\left(a^{\prime }b^{\prime }\right)}_{\mathrm{rms}}^{\prime } & = & a_{\mathrm{rms}}^{\prime }\;b_{\mathrm{rms}}^{\prime }\sqrt{c_{a^{\prime }a^{\prime }b^{\prime }b^{\prime }}\;-\;c_{a^{\prime }b^{\prime }}^{2}} \end{array}$$

(30b) $$\begin{array}{cccccccccccccc} {\left(a^{\prime 2}\right)}^{\prime } & := & a^{\prime 2}-\bar{a^{\prime 2}}\; & \Rightarrow & \;\bar{{\left(a^{\prime 2}\right)}^{\prime 2}} & = & & \bar{{\left(a^{\prime 2}\;-\;\bar{a^{\prime 2}}\right)}^{2}} & = & \bar{a^{\prime 4}}\;-\;{\bar{a^{\prime 2}}}^{2}\; & \overset{\left(2\right)}{\Rightarrow } & \;{\left(a^{\prime 2}\right)}_{\mathrm{rms}}^{\prime } & = & a_{\mathrm{rms}}^{\prime 2}\sqrt{F_{a^{\prime }}\;-\;1} \end{array}$$

Notice that (30a) also implies that for any pair of random variables $\left(a^{\prime },b^{\prime }\right)$,

(30c) $$\begin{array}{c} \bar{{\left(a^{\prime }b^{\prime }\right)}^{\prime 2}}\geq 0\overset{\left(30a\right)}{\Rightarrow }c_{a^{\prime }a^{\prime }b^{\prime }b^{\prime }}\;-\;c_{a^{\prime }b^{\prime }}^{2}\geq 0 \end{array}$$

Using the results (30) for the variances, the following identities for the correlation coefficients between fluctuations of products-of-fluctuations are readily obtained:

(31a) $$\begin{array}{cccccccc} c_{a^{\prime }{\left(a^{\prime 2}\right)}^{\prime }}:= & \frac{\bar{a^{\prime }\left(a^{\prime 2}-\bar{a^{\prime 2}}\right)}}{a_{\mathrm{rms}}^{\prime }\;{\left(a^{\prime 2}\right)}_{\mathrm{rms}}^{\prime }}\overset{\left(30\right)}{=}\frac{\bar{a^{\prime 3}}}{a_{\mathrm{rms}}^{\prime 3}\sqrt{F_{a^{\prime }}-1}} & \Rightarrow & S_{a^{\prime }} & = & c_{a^{\prime }{\left(a^{\prime 2}\right)}^{\prime }} & & \sqrt{F_{a^{\prime }}-1} \end{array}$$

(31b) $$\begin{array}{cccccccc} c_{a^{\prime }{\left(a^{\prime }b^{\prime }\right)}^{\prime }}:= & \frac{\bar{a^{\prime }\left(a^{\prime }b^{\prime }-\bar{a^{\prime }b^{\prime }}\right)}}{a_{\mathrm{rms}}^{\prime }\;{\left(a^{\prime }b^{\prime }\right)}_{\mathrm{rms}}^{\prime }}\overset{\left(30\right)}{=}\frac{\bar{a^{\prime 2}b^{\prime }}}{a^{\prime 2}\;b_{\mathrm{rms}}^{\prime }\sqrt{c_{a^{\prime }a^{\prime }b^{\prime }b^{\prime }}\;-\;c_{a^{\prime }b^{\prime }}^{2}}} & \Rightarrow & c_{a^{\prime }a^{\prime }b^{\prime }} & = & c_{a^{\prime }{\left(a^{\prime }b^{\prime }\right)}^{\prime }} & & \sqrt{c_{a^{\prime }a^{\prime }b^{\prime }b^{\prime }}\;-\;c_{a^{\prime }b^{\prime }}^{2}} \end{array}$$

(31c) $$\begin{array}{cccccccc} c_{{\left(a^{\prime 2}\right)}^{\prime }b^{\prime }}:= & \frac{\bar{\left(a^{\prime 2}-\bar{a^{\prime 2}}\right)b^{\prime }}}{{\left(a^{\prime 2}\right)}_{\mathrm{rms}}^{\prime }\;b_{\mathrm{rms}}^{\prime }}\overset{\left(30\right)}{=}\frac{\bar{a^{\prime 2}b^{\prime }}}{a^{\prime 2}\;b_{\mathrm{rms}}^{\prime }\sqrt{F_{a^{\prime }}-1}} & \Rightarrow & c_{a^{\prime }a^{\prime }b^{\prime }} & = & c_{{\left(a^{\prime 2}\right)}^{\prime }b^{\prime }} & & \sqrt{F_{a^{\prime }}-1} \end{array}$$

(31d) $$\begin{array}{cccccccc} c_{{\left(a^{\prime }b^{\prime }\right)}^{\prime }c^{\prime }}:= & \frac{\bar{\left(a^{\prime }b^{\prime }-\bar{a^{\prime }b^{\prime }}\right)c^{\prime }}}{{\left(a^{\prime }b^{\prime }\right)}_{\mathrm{rms}}^{\prime }\;c_{\mathrm{rms}}^{\prime }}\overset{\left(30\right)}{=}\frac{\bar{a^{\prime }b^{\prime }c^{\prime }}}{a_{\mathrm{rms}}^{\prime }\;b_{\mathrm{rms}}^{\prime }\;c_{\mathrm{rms}}^{\prime }\sqrt{c_{a^{\prime }a^{\prime }b^{\prime }b^{\prime }}\;-\;c_{a^{\prime }b^{\prime }}^{2}}} & \Rightarrow & c_{a^{\prime }b^{\prime }c^{\prime }} & = & c_{{\left(a^{\prime }b^{\prime }\right)}^{\prime }c^{\prime }} & & \sqrt{c_{a^{\prime }a^{\prime }b^{\prime }b^{\prime }}\;-\;c_{a^{\prime }b^{\prime }}^{2}} \end{array}$$

(31e) $$\begin{array}{cccccccc} c_{a^{\prime }{\left(b^{\prime }c^{\prime }\right)}^{\prime }}:= & \frac{\bar{a^{\prime }\left(b^{\prime }c^{\prime }-\bar{b^{\prime }c^{\prime }}\right)}}{a^{\prime }\;{\left(b^{\prime }c^{\prime }\right)}_{\mathrm{rms}}^{\prime }}\overset{\left(30\right)}{=}\frac{\bar{a^{\prime }b^{\prime }c^{\prime }}}{a_{\mathrm{rms}}^{\prime }\;b_{\mathrm{rms}}^{\prime }\;c_{\mathrm{rms}}^{\prime }\sqrt{c_{b^{\prime }b^{\prime }c^{\prime }c^{\prime }}\;-\;c_{b^{\prime }c^{\prime }}^{2}}} & \Rightarrow & c_{a^{\prime }b^{\prime }c^{\prime }} & = & c_{a^{\prime }{\left(b^{\prime }c^{\prime }\right)}^{\prime }} & & \sqrt{c_{b^{\prime }b^{\prime }c^{\prime }c^{\prime }}\;-\;c_{b^{\prime }c^{\prime }}^{2}} \end{array}$$

(31f) $$\begin{array}{cccccccc} c_{b^{\prime }{\left(a^{\prime }c^{\prime }\right)}^{\prime }}:= & \frac{\bar{b^{\prime }\left(a^{\prime }c^{\prime }-\bar{a^{\prime }c^{\prime }}\right)}}{b^{\prime }\;{\left(a^{\prime }c^{\prime }\right)}_{\mathrm{rms}}^{\prime }}\overset{\left(30\right)}{=}\frac{\bar{a^{\prime }b^{\prime }c^{\prime }}}{a_{\mathrm{rms}}^{\prime }\;b_{\mathrm{rms}}^{\prime }\;c_{\mathrm{rms}}^{\prime }\sqrt{c_{a^{\prime }a^{\prime }c^{\prime }c^{\prime }}\;-\;c_{a^{\prime }c^{\prime }}^{2}}} & \Rightarrow & c_{a^{\prime }b^{\prime }c^{\prime }} & = & c_{b^{\prime }{\left(a^{\prime }c^{\prime }\right)}^{\prime }} & & \sqrt{c_{a^{\prime }a^{\prime }c^{\prime }c^{\prime }}\;-\;c_{a^{\prime }c^{\prime }}^{2}} \end{array}$$

These identities express all 3CCs as products of a correlation coefficient of order 2 multiplied by a square root of the 4CCs. Straightforward application of the Schwarz inequality (29) provides upper bounds for the absolute values of the 3CCs:

(32a) $$\begin{array}{cccc} (31a)\mathrm{and}\;(29)\Rightarrow & |S_{a^{\prime }}| & \leq & \sqrt{F_{a^{\prime }}-1} \end{array}$$

(32b) $$\begin{array}{cccc} (31b),(31c),(29)\Rightarrow & |c_{a^{\prime }a^{\prime }b^{\prime }}| & \leq & \min\left(\sqrt{c_{a^{\prime }a^{\prime }b^{\prime }b^{\prime }}\;-\;c_{a^{\prime }b^{\prime }}^{2}},\sqrt{F_{a^{\prime }}-1}\right) \end{array}$$

(32c) $$\begin{array}{cccc} (32b)\Rightarrow & |c_{a^{\prime }b^{\prime }b^{\prime }}| & \leq & \min\left(\sqrt{c_{a^{\prime }a^{\prime }b^{\prime }b^{\prime }}\;-\;c_{a^{\prime }b^{\prime }}^{2}},\sqrt{F_{b^{\prime }}-1}\right) \end{array}$$

(32d) $$\begin{array}{cccc} (31d),(31e),(31f),(29)\Rightarrow & |c_{a^{\prime }b^{\prime }c^{\prime }}| & \leq & \min\left(\sqrt{c_{a^{\prime }a^{\prime }b^{\prime }b^{\prime }}\;-\;c_{a^{\prime }b^{\prime }}^{2}},\sqrt{c_{b^{\prime }b^{\prime }c^{\prime }c^{\prime }}\;-\;c_{b^{\prime }c^{\prime }}^{2}},\sqrt{c_{a^{\prime }a^{\prime }c^{\prime }c^{\prime }}\;-\;c_{a^{\prime }c^{\prime }}^{2}}\right) \end{array}$$

where (32c) is obtained from (32b) by interchanging the random variables a and b. Analogous relations were given in [22], where a different notation was used. They were redemonstrated here for completeness and notational consistency. Inequality (31a) is Pearson’s inequality [35]. The inequalities (32d) that were obtained from identities (31d)–(31f) correspond to ([22], (2.8), p. 13), and the inequalities (32b) that were obtained from identities (31b) and (31c) correspond to ([22], (2.10), p. 14).

There are two upper bounds (32b) for $|c_{a^{\prime }a^{\prime }b^{\prime }}|$, either from joint $\left(a^{\prime },b^{\prime }\right)$-statistics or from the single-variable kurtosis $F_{a^{\prime }}$, while there are three upper bounds (32d) for $|c_{a^{\prime }b^{\prime }c^{\prime }}|$ from the joint statistics of the three couples of variables. Many of these bounds (31) are of the form $c_{a^{\prime }a^{\prime }b^{\prime }b^{\prime }}\;-\;c_{a^{\prime }b^{\prime }}^{2}\;\geq \;0$ (30c). An upper bound for $c_{a^{\prime }a^{\prime }b^{\prime }b^{\prime }}$ can be obtained in terms of the individual kurtoses from

(33a) $$\begin{array}{cccc} c_{{\left(a^{\prime 2}\right)}^{\prime }{\left(b^{\prime 2}\right)}^{\prime }}:= & \frac{\bar{\left(a^{\prime 2}-\bar{a^{\prime 2}}\right)\left(b^{\prime 2}-\bar{b^{\prime 2}}\right)}}{{\left(a^{\prime 2}\right)}_{\mathrm{rms}}^{\prime }\;{\left(b^{\prime 2}\right)}_{\mathrm{rms}}^{\prime }}\overset{\left(30\right)}{=} & & \frac{\bar{a^{\prime 2}b^{\prime 2}}-\bar{a^{\prime 2}}\;\bar{b^{\prime 2}}}{a_{\mathrm{rms}}^{\prime 2}\;b_{\mathrm{rms}}^{\prime 2}\sqrt{F_{a^{\prime }}-1}\sqrt{F_{b^{\prime }}-1}} \end{array}$$

(33b) $$\begin{array}{cccc} c_{{\left(a^{\prime 2}\right)}^{\prime }{\left(a^{\prime }b^{\prime }\right)}^{\prime }}:= & \frac{\bar{\left(a^{\prime 2}-\bar{a^{\prime 2}}\right)\left(a^{\prime }b^{\prime }-\bar{a^{\prime }b^{\prime }}\right)}}{{\left(a^{\prime 2}\right)}_{\mathrm{rms}}^{\prime }\;{\left(a^{\prime }b^{\prime }\right)}_{\mathrm{rms}}^{\prime }}\overset{\left(30\right)}{=} & & \frac{\bar{a^{\prime 3}b^{\prime }}-\bar{a^{\prime 2}}\;\bar{a^{\prime }b^{\prime }}}{a_{\mathrm{rms}}^{\prime 3}\;b_{\mathrm{rms}}^{\prime }\sqrt{F_{a^{\prime }}-1}\sqrt{c_{a^{\prime }a^{\prime }b^{\prime }b^{\prime }}\;-\;c_{a^{\prime }b^{\prime }}^{2}}} \end{array}$$

(33c) $$\begin{array}{cccc} c_{{\left(a^{\prime 2}\right)}^{\prime }{\left(b^{\prime }c^{\prime }\right)}^{\prime }}:= & \frac{\bar{\left(a^{\prime 2}-\bar{a^{\prime 2}}\right)\left(b^{\prime }c^{\prime }-\bar{b^{\prime }c^{\prime }}\right)}}{{\left(a^{\prime 2}\right)}_{\mathrm{rms}}^{\prime }\;{\left(b^{\prime }c^{\prime }\right)}_{\mathrm{rms}}^{\prime }}\overset{\left(30\right)}{=} & & \frac{\bar{a^{\prime 2}b^{\prime }c^{\prime }}-\bar{a^{\prime 2}}\;\bar{b^{\prime }c^{\prime }}}{a_{\mathrm{rms}}^{\prime 2}\;b_{\mathrm{rms}}^{\prime }\;c_{\mathrm{rms}}^{\prime }\sqrt{F_{a^{\prime }}-1}\sqrt{c_{b^{\prime }b^{\prime }c^{\prime }c^{\prime }}\;-\;c_{b^{\prime }c^{\prime }}^{2}}} \end{array}$$

(33d) $$\begin{array}{cccc} c_{{\left(a^{\prime }b^{\prime }\right)}^{\prime }{\left(a^{\prime }c^{\prime }\right)}^{\prime }}:= & \frac{\bar{\left(a^{\prime }b^{\prime }-\bar{a^{\prime }b^{\prime }}\right)\left(a^{\prime }c^{\prime }-\bar{a^{\prime }c^{\prime }}\right)}}{{\left(a^{\prime }b^{\prime }\right)}_{\mathrm{rms}}^{\prime }\;{\left(a^{\prime }c^{\prime }\right)}_{\mathrm{rms}}^{\prime }}\overset{\left(30\right)}{=} & & \frac{\bar{a^{\prime 2}b^{\prime }c^{\prime }}-\bar{a^{\prime }b^{\prime }}\;\bar{a^{\prime }c^{\prime }}}{a_{\mathrm{rms}}^{\prime 2}\;b_{\mathrm{rms}}^{\prime }\;c_{\mathrm{rms}}^{\prime }\sqrt{c_{a^{\prime }a^{\prime }b^{\prime }b^{\prime }}\;-\;c_{a^{\prime }b^{\prime }}^{2}}\sqrt{c_{a^{\prime }a^{\prime }c^{\prime }c^{\prime }}\;-\;c_{a^{\prime }c^{\prime }}^{2}}} \end{array}$$

leading to the identities

(34a) $$\begin{array}{cccccc} \left(33a)\mathrm{and}\;(2\right) & \Rightarrow & c_{a^{\prime }a^{\prime }b^{\prime }b^{\prime }}-1 & = & c_{{\left(a^{\prime 2}\right)}^{\prime }{\left(b^{\prime 2}\right)}^{\prime }} & \sqrt{F_{a^{\prime }}-1}\sqrt{F_{b^{\prime }}-1} \end{array}$$

(34b) $$\begin{array}{cccccc} \left(33b)\mathrm{and}\;(2\right) & \Rightarrow & c_{a^{\prime }a^{\prime }a^{\prime }b^{\prime }}-c_{a^{\prime }b^{\prime }} & = & c_{{\left(a^{\prime 2}\right)}^{\prime }{\left(a^{\prime }b^{\prime }\right)}^{\prime }} & \sqrt{F_{a^{\prime }}-1}\sqrt{c_{a^{\prime }a^{\prime }b^{\prime }b^{\prime }}\;-\;c_{a^{\prime }b^{\prime }}^{2}} \end{array}$$

(34c) $$\begin{array}{cccccc} \left(33c)\mathrm{and}\;(2\right) & \Rightarrow & c_{a^{\prime }a^{\prime }b^{\prime }c^{\prime }}-c_{b^{\prime }c^{\prime }} & = & c_{{\left(a^{\prime 2}\right)}^{\prime }{\left(b^{\prime }c^{\prime }\right)}^{\prime }} & \sqrt{F_{a^{\prime }}-1}\sqrt{c_{b^{\prime }b^{\prime }c^{\prime }c^{\prime }}\;-\;c_{b^{\prime }c^{\prime }}^{2}} \end{array}$$

(34d) $$\begin{array}{cccccc} \left(33d)\mathrm{and}\;(2\right) & \Rightarrow & c_{a^{\prime }a^{\prime }b^{\prime }c^{\prime }}-c_{a^{\prime }b^{\prime }}\;c_{a^{\prime }c^{\prime }} & = & c_{{\left(a^{\prime }b^{\prime }\right)}^{\prime }{\left(a^{\prime }c^{\prime }\right)}^{\prime }} & \sqrt{c_{a^{\prime }a^{\prime }b^{\prime }b^{\prime }}\;-\;c_{a^{\prime }b^{\prime }}^{2}}\sqrt{c_{a^{\prime }a^{\prime }c^{\prime }c^{\prime }}\;-\;c_{a^{\prime }c^{\prime }}^{2}} \end{array}$$

Identity (34a) can be used in (34b)–(34d) to express the RHSs in terms of individual kurtoses. The above identities (34) contain the 2CCs $\left\{c_{{\left(a^{\prime 2}\right)}^{\prime }{\left(b^{\prime 2}\right)}^{\prime }},c_{{\left(a^{\prime 2}\right)}^{\prime }{\left(a^{\prime }b^{\prime }\right)}^{\prime }},c_{{\left(a^{\prime 2}\right)}^{\prime }{\left(b^{\prime }c^{\prime }\right)}^{\prime }},c_{{\left(a^{\prime }b^{\prime }\right)}^{\prime }{\left(a^{\prime }c^{\prime }\right)}^{\prime }}\right\}$ which are bounded by the covariance inequality (29), readily providing bounds for the 4CCs:

(35a) $$\begin{array}{cccccccc} \left(34a)\mathrm{and}\;(29\right) & \Rightarrow & c_{a^{\prime }b^{\prime }}^{2}\overset{\left(30c\right)}{\leq } & c_{a^{\prime }a^{\prime }b^{\prime }b^{\prime }} & \leq & 1+ & & \sqrt{F_{a^{\prime }}-1}\sqrt{F_{b^{\prime }}-1} \end{array}$$

(35b) $$\begin{array}{cccccccc} \left(34b)\mathrm{and}\;(29\right) & \Rightarrow & & |c_{a^{\prime }a^{\prime }a^{\prime }b^{\prime }}-c_{a^{\prime }b^{\prime }}| & \leq & & & \sqrt{F_{a^{\prime }}-1}\sqrt{c_{a^{\prime }a^{\prime }b^{\prime }b^{\prime }}\;-\;c_{a^{\prime }b^{\prime }}^{2}} \end{array}$$

(35c) $$\begin{array}{cccccccc} \left(34c)\mathrm{and}\;(29\right) & \Rightarrow & & |c_{a^{\prime }a^{\prime }b^{\prime }c^{\prime }}-c_{b^{\prime }c^{\prime }}| & \leq & & & \sqrt{F_{a^{\prime }}-1}\sqrt{c_{b^{\prime }b^{\prime }c^{\prime }c^{\prime }}\;-\;c_{b^{\prime }c^{\prime }}^{2}} \end{array}$$

(35d) $$\begin{array}{cccccccc} \left(34d)\mathrm{and}\;(29\right) & \Rightarrow & & |c_{a^{\prime }a^{\prime }b^{\prime }c^{\prime }}-c_{a^{\prime }b^{\prime }}\;c_{a^{\prime }c^{\prime }}| & \leq & & & \sqrt{c_{a^{\prime }a^{\prime }b^{\prime }b^{\prime }}\;-\;c_{a^{\prime }b^{\prime }}^{2}}\sqrt{c_{a^{\prime }a^{\prime }c^{\prime }c^{\prime }}\;-\;c_{a^{\prime }c^{\prime }}^{2}} \end{array}$$

where again, (34a) and (35a) can be used to express these bounds in terms of individual (single-variable) kurtoses, but this would render the bounds less sharp. Inequalities (35) are related to ([22], Theorem 3, p. 14).

Quantities of the form $c_{a^{\prime }a^{\prime }b^{\prime }b^{\prime }}\;-\;c_{a^{\prime }b^{\prime }}^{2}$ appear very frequently in the above identities and inequalities. Through construction as the average of a product of squares $c_{a^{\prime }a^{\prime }b^{\prime }b^{\prime }}\;\geq \;0$ is positive (30c). However, a stricter lower bound can be obtained by combining the inequalities (32b)–(32d) with (35a), *viz*

(36a) $$\begin{array}{c} \left(32\right)\mathrm{and}\;\left(35a\right)\;\Rightarrow \;0\;\leq \;c_{a^{\prime }b^{\prime }}^{2}\;+\;\max\left(c_{a^{\prime }a^{\prime }b^{\prime }}^{2},c_{a^{\prime }b^{\prime }b^{\prime }}^{2},c_{a^{\prime }b^{\prime }c^{\prime }}^{2}\right)\;\leq \;c_{a^{\prime }a^{\prime }b^{\prime }b^{\prime }}\;\leq \;1\;+\;\sqrt{F_{a^{\prime }}\;-\;1}\sqrt{F_{b^{\prime }}\;-\;1} \end{array}$$

where the lower bound includes the maximum for any random variable $c^{\prime }$ other than $\left\{a^{\prime },b^{\prime }\right\}$. Notice that (36a) also implies

(36b) $$\begin{array}{c} \left(36a\right)\;\Rightarrow \;0\;\leq \;\max\left(c_{a^{\prime }a^{\prime }b^{\prime }}^{2},c_{a^{\prime }b^{\prime }b^{\prime }}^{2},c_{a^{\prime }b^{\prime }c^{\prime }}^{2}\right)\;\leq \;c_{a^{\prime }a^{\prime }b^{\prime }b^{\prime }}\;-\;c_{a^{\prime }b^{\prime }}^{2}\;\leq \;1\;+\;\sqrt{F_{a^{\prime }}\;-\;1}\sqrt{F_{b^{\prime }}\;-\;1} \end{array}$$

since invariably, $-c_{a^{\prime }b^{\prime }}^{2}\;\leq \;0$.

These upper bounds, although sharper than more naive approaches, are still not very restrictive, while the sharper bounds obtained by Lumley [20] are only valid for particular cases. Notice that in the univariate case, sharper estimates are obtained when the pdf is unimodal, e.g., Pearson’s inequality [35] for unimodal distributions is sharpened to $S_{{\left(\cdot \right)}^{\prime }}^{2}\;\leq \;F_{{\left(\cdot \right)}^{\prime }}-\frac{189}{125}$ [21]. This suggests that the above results can probably be slightly sharpened in the case of unimodal trivariate distributions [37].

## 6. Implications for **$\left(p^{\prime },\rho^{\prime },T^{\prime }\right)$**-Correlations

The general statistical inequalities (Section 5) and the exact and approximate relations derived from the fluctuating EoS (Section 4) provide explanations for some of the DNS results for TPC flows (Figure 1 and Figure 2) and for the approximation errors (Figure 3, Figure 4 and Figure 5) of the linearized relations (22).

### 6.1. Profiles of 3CCs

The analysis in this subsection applies to the specific case of canonical compressible TPC flow [5]. In a large part of this class of flows, except in the wake region, $c_{\rho^{\prime }\rho^{\prime }T^{\prime }}$ and $c_{\rho^{\prime }T^{\prime }T^{\prime }}$ are of opposite signs (Figure 2b,d). According to (25a), this is consistent with the fact that $|c_{p^{\prime }\rho^{\prime }T^{\prime }}|$ takes small values everywhere except in the wake region (Figure 2a). In the buffer region ($5\;⪅\;y^{★}\;⪅\;40$) of compressible TPC flows, where $|c_{p^{\prime }\rho^{\prime }T^{\prime }}|\;\ll \;1$ (Figure 2a) and ${C\;V}_{\;\rho^{\prime }}\;≊\;{C\;V}_{\;T^{\prime }}$ (Figure 1a,c), (25a) implies $c_{\rho^{\prime }\rho^{\prime }T^{\prime }}\;≊\;-c_{\rho^{\prime }T^{\prime }T^{\prime }}$, in agreement with DNS data (Figure 2b,d). Further away from the wall, as long as $|c_{p^{\prime }\rho^{\prime }T^{\prime }}|\;\ll \;1$ (Figure 2a), (25a) implies the more general relation $c_{\rho^{\prime }\rho^{\prime }T^{\prime }}\;{C\;V}_{\;\rho^{\prime }}\;≊\;-c_{\rho^{\prime }T^{\prime }T^{\prime }}\;{C\;V}_{\;T^{\prime }}$ which takes into account the differences in fluctuation intensities ${C\;V}_{\;\rho^{\prime }}$ and ${C\;V}_{\;T^{\prime }}$. Nearer to the centerline, $|c_{p^{\prime }\rho^{\prime }T^{\prime }}|$ increases (Figure 2a), modifying this relation, with $c_{\rho^{\prime }\rho^{\prime }T^{\prime }}$ and $c_{\rho^{\prime }T^{\prime }T^{\prime }}$ being of the same sign in the centerline region (Figure 2b,d). Similarly, $|c_{p^{\prime }\rho^{\prime }T^{\prime }}|\;\ll \;1$ in the buffer zone (Figure 2a) justifies according to (25b) that $c_{p^{\prime }T^{\prime }T^{\prime }}$ and $c_{p^{\prime }p^{\prime }T^{\prime }}$ are of the same sign (Figure 2e,g) there, and (25c) explains why $c_{p^{\prime }\rho^{\prime }\rho^{\prime }}$ and $c_{p^{\prime }p^{\prime }\rho^{\prime }}$ are of the same sign (Figure 2c,f).

### 6.2. Linearization Error in Relations Between 3CCs

The breakdown of the linearized approximation (22f) for $c_{p^{\prime }\rho^{\prime }T^{\prime }}$, for TPC flows at ${\bar{M}}_{{\mathrm{CL}}_{x}}\;⪆\;2$ (Figure 4n,o), is easily explained from inequality (36), written for $c_{\rho^{\prime }\rho^{\prime }T^{\prime }T^{\prime }}$:

(37) $$\begin{array}{c} \left(36\right)\Rightarrow 0\leq \max\left(c_{\rho^{\prime }\rho^{\prime }T^{\prime }}^{2},c_{\rho^{\prime }T^{\prime }T^{\prime }}^{2},c_{p^{\prime }\rho^{\prime }T^{\prime }}^{2}\right)\leq c_{\rho^{\prime }\rho^{\prime }T^{\prime }T^{\prime }}-c_{\rho^{\prime }T^{\prime }}^{2}\leq 1+\sqrt{F_{\rho^{\prime }}-1}\sqrt{F_{T^{\prime }}-1} \end{array}$$

which provides a lower bound for the linearization error (23f) of the approximate relation for $c_{p^{\prime }\rho^{\prime }T^{\prime }}$ (22f)

(38) $$\begin{array}{c} \mathrm{error}\left(c_{p^{\prime }\rho^{\prime }T^{\prime }}\right)\overset{\left(23f)\mathrm{and}\;(37\right)}{\geq }\max\left(c_{\rho^{\prime }\rho^{\prime }T^{\prime }}^{2},c_{\rho^{\prime }T^{\prime }T^{\prime }}^{2},c_{p^{\prime }\rho^{\prime }T^{\prime }}^{2}\right)\;\frac{{C\;V}_{\;\rho^{\prime }}}{{C\;V}_{\;p^{\prime }}}\;{C\;V}_{\;T^{\prime }}-c_{p^{\prime }\rho^{\prime }T^{\prime }}\;c_{\rho^{\prime }T^{\prime }}\;{C\;V}_{\;\rho^{\prime }}\;{C\;V}_{\;T^{\prime }} \end{array}$$

The error (23f) is maximal (Figure 4n,o) in the buffer region ($5\;⪅\;y^{★}\;⪅\;40$) where the peak of ${C\;V}_{\;T^{\prime }}$ occurs (Figure 1c). In that region, $\max\left(c_{\rho^{\prime }\rho^{\prime }T^{\prime }}^{2},c_{\rho^{\prime }T^{\prime }T^{\prime }}^{2},c_{p^{\prime }\rho^{\prime }T^{\prime }}^{2}\right)\;\in \;\left[1,2.25\right]$ (Figure 2a,b,d), and the ratio ${C\;V}_{\;\rho^{\prime }}/{C\;V}_{\;p^{\prime }}\;\in \;\left[3.5,4\right]$ ([4], Figure 3, p. 458). Combining these values with the very approximate relation ${C\;V}_{\;T^{\prime }}\;≊\;0.02{\bar{M}}_{{\mathrm{CL}}_{x}}^{2}$ (Figure 1c) determines through (38) an estimate of the lower bound of the leading term of the error, $\mathrm{error}\left(c_{p^{\prime }\rho^{\prime }T^{\prime }}\right)\;⪆\;\alpha \;{\bar{M}}_{{\mathrm{CL}}_{x}}^{2}$, with α∈[0.07,0.18], which is coherent with the observed errors (Figure 4n,o) of the linearized approximation (23f).

However, the general statistical inequalities (Section 5) do not provide such lower bounds for the other 4CC expressions that determine the leading-order term of the approximation error (23) of the linearized relations (22). Further work is needed to determine whether the difference in the linearized approximation accuracy (Figure 3 and Figure 4) between the robust relations (22a)–(22c) and the fragile relations (22d)–(22f) is specific to the turbulence structure of canonical TPC flows or more general.

## 7. Conclusions

This paper provides the mathematical framework for the study of the exact equations satisfied by *n*-order correlations between fluctuations in the basic thermodynamic variables $\left\{p^{\prime },\rho^{\prime },T^{\prime }\right\}$ that are imposed by the dilute-gas EoS, which describes with satisfactory accuracy most aerodynamic flows. All correlations between $\left\{p^{\prime },\rho^{\prime },T^{\prime }\right\}$ can be expressed in terms of $\left(\rho^{\prime },T^{\prime }\right)$-statistics, but with an increasing correlation order, progressively, a larger number of higher-order $\left(\rho^{\prime },T^{\prime }\right)$-statistics are involved.

Systems of $\frac{n(n+1)}{2}$ equations between the $\frac{\left(n+1)(n+2\right)}{2}$ correlation coefficients of order *n* (*n*CCs) are readily derived from the fluctuating EoS ($\forall \;n\;\in \;N_{\geq 2}$). The nonlinear terms in the equations for *n*CCs are $\propto \;{C\;V}_{\;T^{\prime }}$ and contain an (n+1)CC. At the weakly compressible turbulence limit, $\max\left({C\;V}_{\;p^{\prime }},{C\;V}_{\;\rho^{\prime }},{C\;V}_{\;T^{\prime }}\right)\;\ll \;1$, these systems can be linearized and solved in terms of n+1 linearly independent *n*CCs, implying that a Mach-independent thermodynamic turbulence structure is reached, at a sufficiently low Mach number, for a given type of flow. Nonetheless, the study of the system for 3CCs shows that not all 4-tuples of 3CCs are linearly independent, as illustrated by several relations expressing $c_{p^{\prime }\rho^{\prime }T^{\prime }}$ in terms of various couples of other 3CCs or in terms of the individual skewnesses. Several possible choices of 4-tuples of linearly independent 3CCs were explored.

However, the Mach number limit, below which the weakly compressible turbulence linearized approximation holds, depends not only on the particular flow but also on the order of correlations that is considered (diminishing with an increasing correlation order). For instance, for compressible turbulent plane channel (TPC) flows, the 2CC system can be linearized with acceptable (respectively, very good) accuracy for ${\bar{M}}_{{\mathrm{CL}}_{x}}\;\leq \;2$ (respectively, ${\bar{M}}_{{\mathrm{CL}}_{x}}\;\leq \;1.5$), whereas for the 3CCs system, these limits are ${\bar{M}}_{{\mathrm{CL}}_{x}}\;\leq \;1.5$ (respectively, ${\bar{M}}_{{\mathrm{CL}}_{x}}\;\leq \;1$). This is easily explained by the fact that the linearization error for the system of 2CCs is proportional to 3CCs, whereas the linearization error for the system of 3CCs is proportional to 4CCs, which are generally larger. This effect generalizes to the linearization error of the system of *n*CCs, which is proportionnal to (n+1)CCs. With an increasing correlation order *n*, the thermodynamic turbulence structure of *n*CCs becomes Mach-dependent at progressively lower Mach numbers.

Recent results on the identities and bounds of multivariate skewnesses and flatnesses were applied to 3CCs and 4CCs of $\left\{p^{\prime },\rho^{\prime },T^{\prime }\right\}$ and used in the analysis of the linearization error of the equations between 3CCs of $\left\{p^{\prime },\rho^{\prime },T^{\prime }\right\}$.

DNS data for 3CCs of $\left\{p^{\prime },\rho^{\prime },T^{\prime }\right\}$ in TPC flows, covering the range ${Re}_{\tau^{★}}\;\in \;\left[97,983\right]$ and ${\bar{M}}_{{\mathrm{CL}}_{x}}\;\in \;\left[0.32,2.49\right]$, were analyzed to determine the influence of Mach and Reynolds numbers. These data reveal that the pressure skewness $S_{p^{\prime }}$ robustly defines various zones of the flow and that the temperature pdf ($S_{T^{\prime }}$ and $F_{T^{\prime }}$ data) is not very sensitive to ${\bar{M}}_{{\mathrm{CL}}_{x}}$, except perhaps near the centerline. The data also show that a canonical TPC flow reaches a Mach-independent thermodynamic turbulence structure when ${\bar{M}}_{{\mathrm{CL}}_{x}}\;\leq \;0.8$. On the other hand, even at ${Re}_{\tau^{★}}\;=\;1000$, some statistics, in particular the skewnesses of density $S_{\rho^{\prime }}$ and of temperature $S_{T^{\prime }}$, have not reached a Reynolds-asymptotic inner/overlap law yet, highlighting the need for computations at higher Reynolds numbers.

## Figures and Tables

**Table 1:** Number of *n*-order moments between $\left\{p^{\prime },\rho^{\prime },T^{\prime }\right\}$-fluctuations, number of Equations (12) and (13) implied by the fluctuating EoS (7) between *n*-order moments and number of independent *n*-order moments (at the weakly compressible limit).

*n*	$\frac{\left(n+1)(n+2\right)}{2}$ Moments	$\frac{n(n+1)}{2}$ Equation (13)	(n+1) Independent
2	6	3	3
3	10	6	4
4	15	10	5
5	21	15	6
6	28	21	7

**Figure A1:**
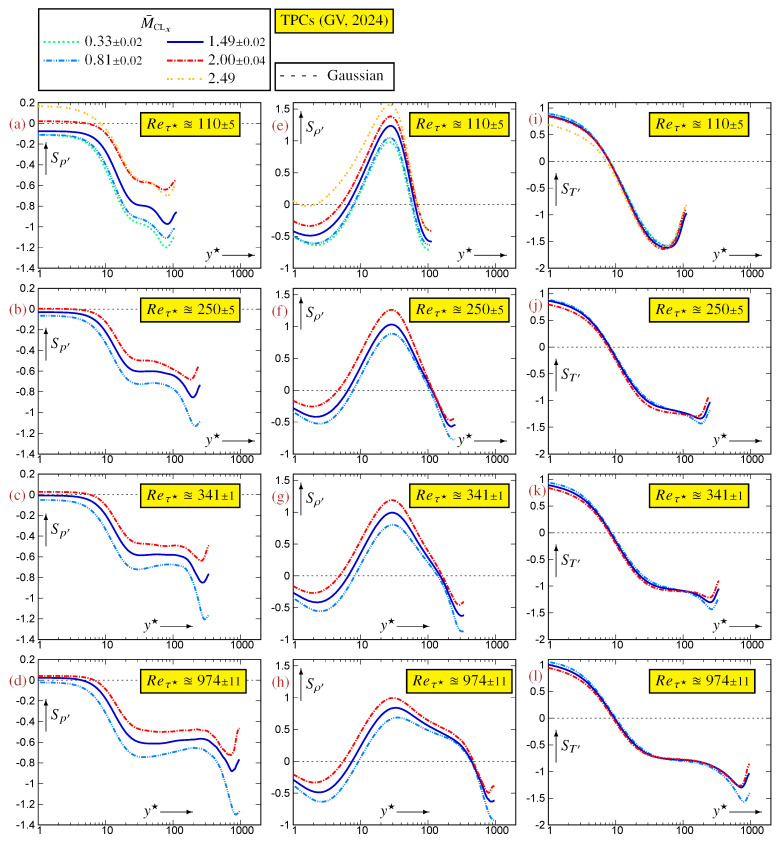
Profiles of skewness $\left\{S_{p^{\prime }}\;\left(a–d\right),S_{\rho^{\prime }}\;\left(e–h\right),S_{T^{\prime }}\;\left(i–l\right)\right\}$ of thermodynamic fluctuations, plotted against the HCB-scaled nondimensional wall-distance $y^{★}$ (logscale), for varying $0.32\;\leq \;{\bar{M}}_{{\mathrm{CL}}_{x}}\;\leq \;2.49$ at nearly constant $Re_{\tau^{★}}\;\in \;\left\{110,250,340,1000\right\}$, using DNS data [12] for turbulent plane channel flows [10,11].

**Figure A2:**
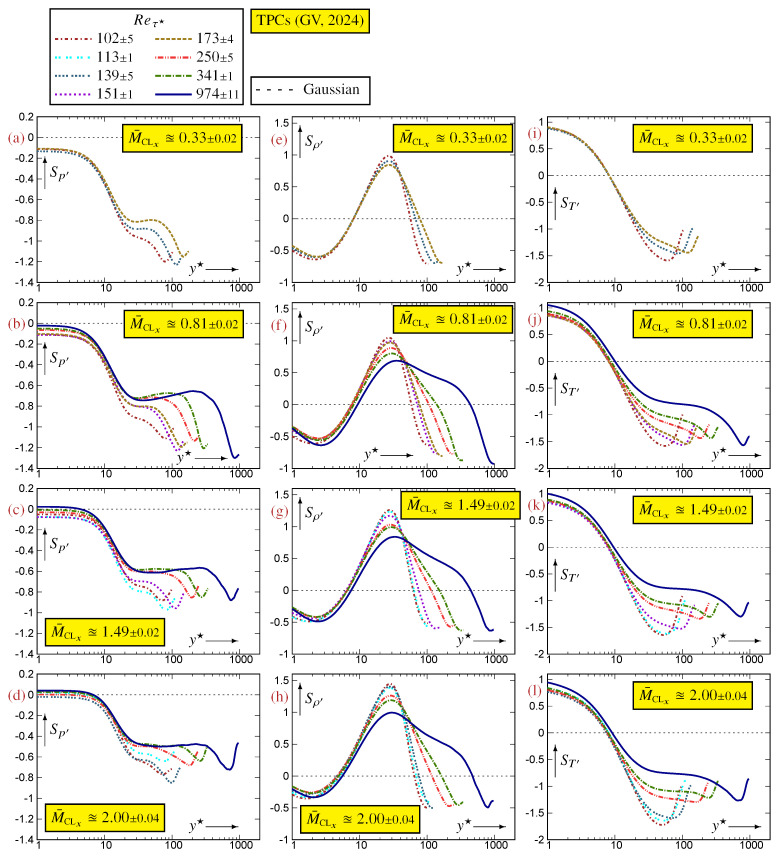
Profiles of skewness $\left\{S_{p^{\prime }}\;\left(a–d\right),S_{\rho^{\prime }}\;\left(e–h\right),S_{T^{\prime }}\;\left(i–l\right)\right\}$ of thermodynamic fluctuations, plotted against the HCB-scaled nondimensional wall-distance $y^{★}$ (logscale), for varying $97\;\leq \;Re_{\tau^{★}}\;\leq \;985$ at nearly constant ${\bar{M}}_{{\mathrm{CL}}_{x}}\;\in \;\left\{0.33,0.80,1.50,2.00\right\}$, using DNS data [12] for turbulent plane channel flows [10,11].

**Figure A5:**
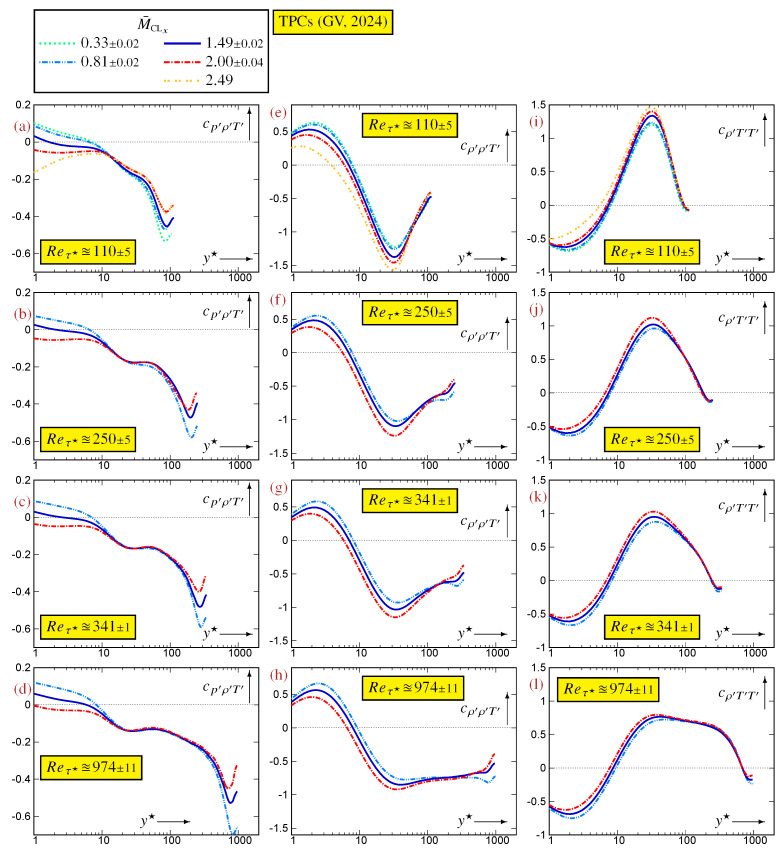
Profiles of correlation coefficients $\left\{c_{p^{\prime }\rho^{\prime }T^{\prime }}\;\left(a–d\right),c_{\rho^{\prime }\rho^{\prime }T^{\prime }}\;\left(e–h\right),c_{\rho^{\prime }T^{\prime }T^{\prime }}\;\left(i–l\right)\right\}$ between thermodynamic fluctuations, plotted against the HCB-scaled nondimensional wall-distance $y^{★}$ (logscale), for varying $0.32\;\leq \;{\bar{M}}_{{\mathrm{CL}}_{x}}\;\leq \;2.49$ at nearly constant $Re_{\tau^{★}}\;\in \;\left\{110,250,340,1000\right\}$, using DNS data [12] for turbulent plane channel flows [10,11].

**Figure A6:**
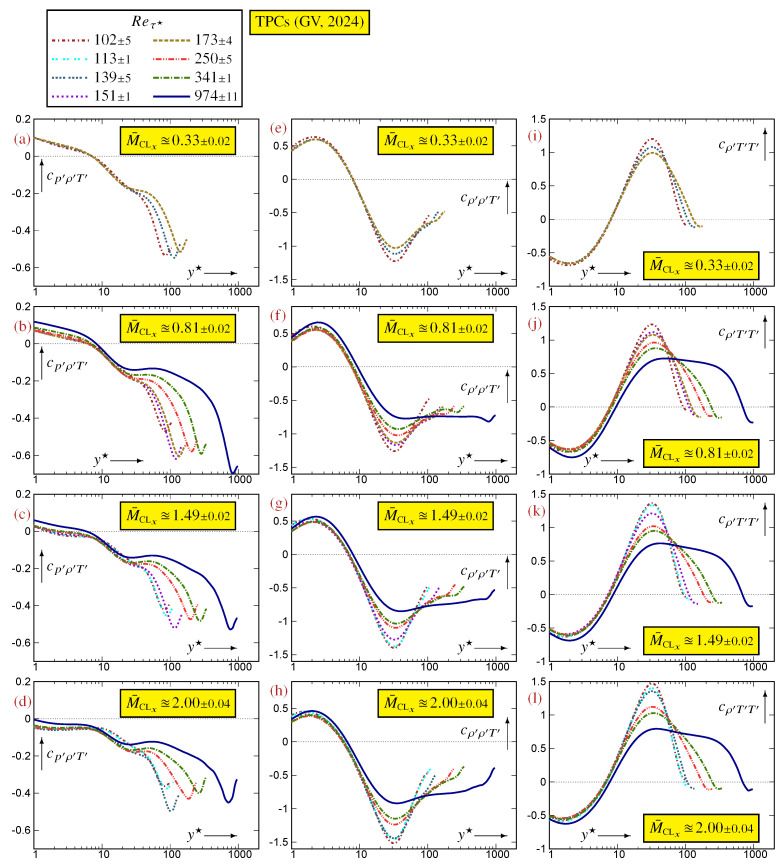
Profiles of the correlation coefficients $\left\{c_{p^{\prime }\rho^{\prime }T^{\prime }}\;\left(a–d\right),c_{\rho^{\prime }\rho^{\prime }T^{\prime }}\;\left(e–h\right),c_{\rho^{\prime }T^{\prime }T^{\prime }}\;\left(i–l\right)\right\}$ between thermodynamic fluctuations, plotted against the HCB-scaled nondimensional wall-distance $y^{★}$ (logscale), for varying $97\;\leq \;Re_{\tau^{★}}\;\leq \;985$ at nearly constant ${\bar{M}}_{{\mathrm{CL}}_{x}}\;\in \;\left\{0.33,0.80,1.50,2.00\right\}$, using DNS data [12] for turbulent plane channel flows [10,11].

**Figure A7:**
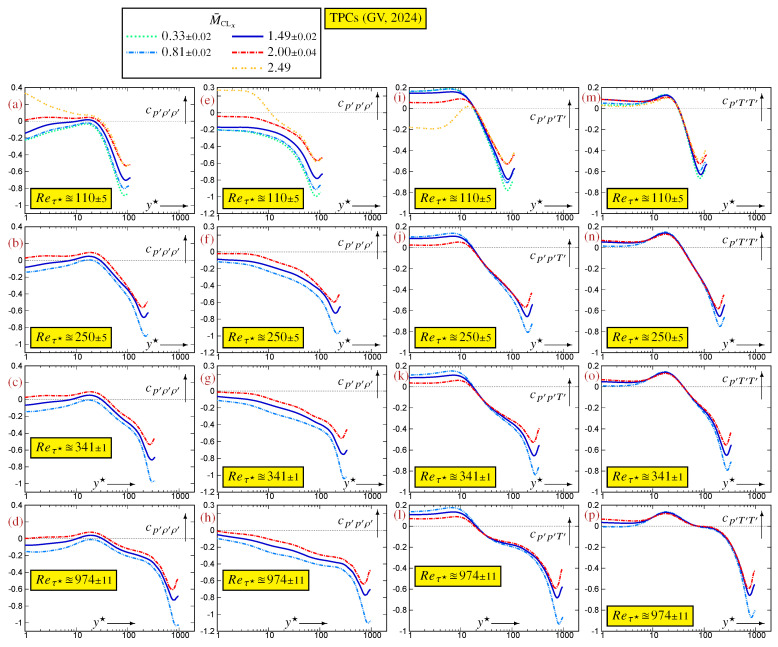
Profiles of the correlation coefficients $\left\{c_{p^{\prime }\rho^{\prime }\rho^{\prime }}\;\left(a–d\right),c_{p^{\prime }p^{\prime }\rho^{\prime }}\;\left(e–h\right),c_{p^{\prime }p^{\prime }T^{\prime }}\;\left(i–l\right),c_{p^{\prime }T^{\prime }T^{\prime }}\;\left(m–p\right)\right\}$ between thermodynamic fluctuations, plotted against the HCB-scaled nondimensional wall-distance $y^{★}$ (logscale), for varying $0.32\;\leq \;{\bar{M}}_{{\mathrm{CL}}_{x}}\;\leq \;2.49$ at nearly constant $Re_{\tau^{★}}\;\in \;\left\{110,250,340,1000\right\}$, using DNS data [12] for turbulent plane channel flows [10,11].

**Figure A8:**
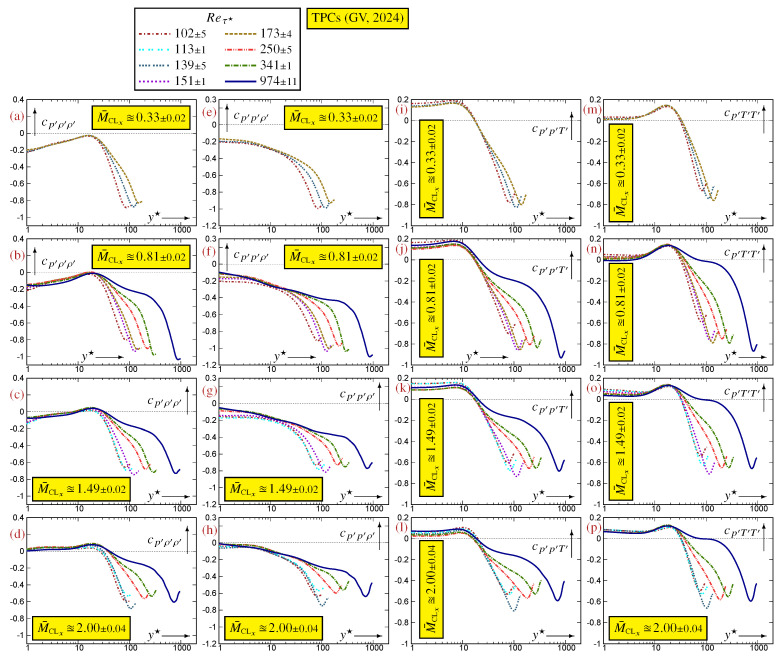
Profiles of the correlation coefficients $\left\{c_{p^{\prime }\rho^{\prime }\rho^{\prime }}\;\left(a–d\right),c_{p^{\prime }p^{\prime }\rho^{\prime }}\;\left(e–h\right),c_{p^{\prime }p^{\prime }T^{\prime }}\;\left(i–l\right),c_{p^{\prime }T^{\prime }T^{\prime }}\;\left(m–p\right)\right\}$ between thermodynamic fluctuations, plotted against the HCB-scaled nondimensional wall-distance $y^{★}$ (logscale), for varying $97\;\leq \;Re_{\tau^{★}}\;\leq \;985$ at nearly constant ${\bar{M}}_{{\mathrm{CL}}_{x}}\;\in \;\left\{0.33,0.80,1.50,2.00\right\}$, using DNS data [12] for turbulent plane channel flows [10,11].

**Figure A9:**
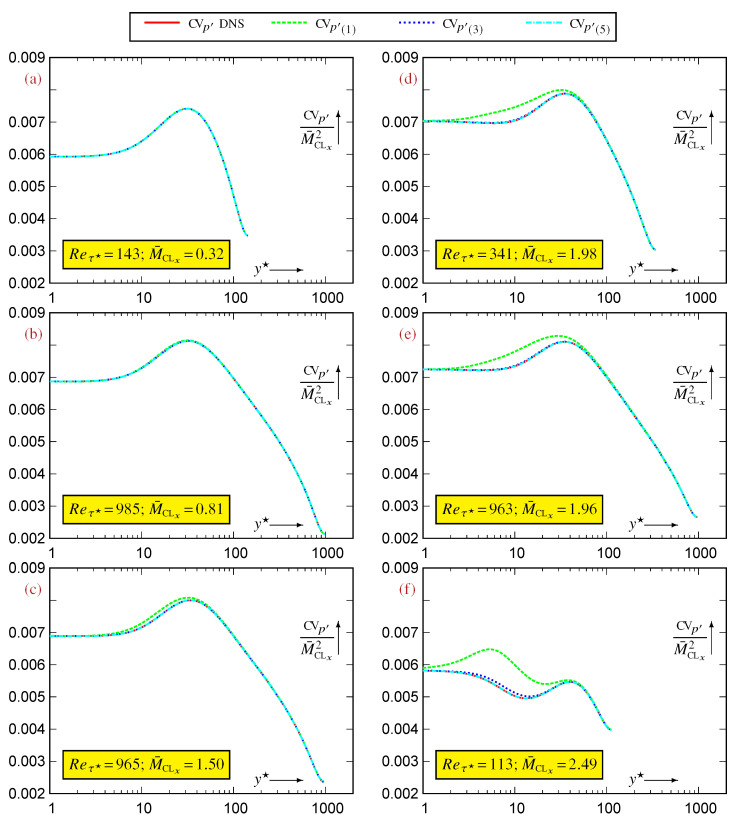
Convergence of successive approximations ${{C\;V}_{\;p^{\prime }}}_{\;(2n-1)}$ (A12) to the ${C\;V}_{\;p^{\prime }}$ profile, evaluated using DNS data [12] for compressible turbulent plane channel flows (**a**–**f**) in the range ${Re}_{\tau^{★}}\in \left[113,985\right]$ and ${\bar{M}}_{{\mathrm{CL}}_{x}}\in \left[0.32,2.49\right]$, plotted against the HCB-scaled nondimensional wall-distance $y^{★}$ (logscale).

## Data Availability

The DNS data are available at Gerolymos, G. A.; Vallet, I. (2024), “Compressible turbulent plane channel DNS database”, Mendeley Data, V1, doi:10.17632/wt8t5kxzbs.1.

## Abbreviations

The following abbreviations are used in this manuscript:

CC, CCs	correlation coefficients
*n*CCs, 2CCs, 3CCs, 4CCs	*n*-order, 2-order, 3-order, 4-order CCs
CV, CVs	coefficient(s) of variation
DNS	direct numerical simulation
HCB	Huang–Coleman–Bradshaw [17]
HoTs, *n*oTs	higher-order terms, *n*-order terms
pdf	probability density function
rms	root-mean-square
TPC	turbulent plane channel

## Appendix A. Does Favre-Averaging Simplify the Equations?

The main reason for using Favre decomposition (4) in transport equations [2,38] is essentially the simplification of triple (mean flow) or quadruple (Reynolds-stress transport) products of density with fluctuations in other flow quantities (e.g., Reynolds stresses $-\;\bar{\rho u_{i}^{\prime \prime }u_{j}^{\prime \prime }}$, turbulent heatfluxes $\bar{\rho h^{\prime \prime }u_{i}^{\prime \prime }}$ or diffusion fluxes of the Reynolds stresses by the fluctuating velocity field $-\bar{\rho u_{i}^{\prime \prime }u_{j}^{\prime \prime }u_{k}^{\prime \prime }}$), where mass-weighting lumps $\rho^{\prime }$-related higher-order moments (HoMs) into the average. This approach greatly simplifies the mean flow equations compared to a Reynolds decomposition approach, but Reynolds decomposition is required for the molecular stresses and the heatfluxes ([38], (3), pp. 190–191), also persisting in Reynolds stress transport ([38], (2), p. 189). We remind the reader here that *Favre averaging does not commute with differentiation* and that expressions noted as ${\tilde{\tau }}_{ij}$ or ${\tilde{q}}_{i}$ simply because the differentiated quantity is a Favre average *are mathematical errors* [2]. The exact equations highlighting this (unfortunately widespread) erroneous notation can be found in ([2], (2.7,2.8), pp. 711–712). The coexistence of Favre and Reynolds decompositions is even more pronounced in the transport equations for the thermodynamic variables [2], where averages weighted by 1/T or by the molecular viscosity μ or the molecular heat conductivity λ are useful for simplifying the expressions. Recall that the original Favre papers [39,40] introduced general (not only mass-weighted) averages. The point made here is that Favre and Reynolds decompositions always coexist in the equations, and that the molecular stresses $\tau_{ij}$ and heatfluxes $q_{i}$ are invariably Reynolds-decomposed. Therefore, the eventual usefulness of weighted averages lies in their ability to simplify the final equations.

The fluctuating EoS is greatly compacted using Favre decomposition of temperature, $T\;=\;\tilde{T}\;+\;T^{\prime \prime }$:

(A1a) $$\begin{array}{cc} Z\left(\rho ,T\right)=1\overset{\left(1)\mathrm{and}\;(4\right)}{\Rightarrow } & \bar{p}=\bar{\rho }R_{g}\tilde{T} \end{array}$$

(A1b) $$\begin{array}{cc} \overset{\left(4\right)}{\Rightarrow } & \frac{p^{\prime }}{\bar{p}}=\frac{\rho^{\prime }}{\bar{\rho }}+\frac{\rho T^{\prime \prime }}{\bar{\rho }\tilde{T}} \end{array}$$

with all terms related to the fluctuating density lumped into the product $\rho T^{\prime \prime }$. However, all equations result from Reynolds averaging (Favre averaging is ρ-weighted Reynolds averaging), and trying to develop equations for higher-order moments (HoMs) does not lead to a simplified system. For instance, using $\left\{p^{\prime },\rho^{\prime },T^{\prime \prime }\right\}$ as the fluctuating thermodynamic variables, and multiplying the Favre-fluctuating EoS (A1b) by these variables, yields

(A2a) $$\begin{array}{cccccccccccc} \frac{\bar{p^{\prime 2}}}{\bar{p}} & \overset{\left(A1b\right)}{=} & \frac{\bar{p^{\prime }\rho^{\prime }}}{\bar{\rho }} & + & \frac{\bar{\rho p^{\prime }T^{\prime \prime }}}{\bar{\rho }\tilde{T}} & \overset{\left(4\right)}{\Rightarrow } & & \frac{\bar{p^{\prime 2}}}{\bar{p}} & = & \frac{\bar{p^{\prime }\rho^{\prime }}}{\bar{\rho }} & + & \frac{\tilde{p^{\prime }T^{\prime \prime }}}{\tilde{T}} \end{array}$$

(A2b) $$\begin{array}{cccccccccccc} \frac{\bar{p^{\prime }\rho^{\prime }}}{\bar{p}} & \overset{\left(A1b\right)}{=} & \frac{\bar{\rho^{\prime 2}}}{\bar{\rho }} & + & \frac{\bar{\rho \rho^{\prime }T^{\prime \prime }}}{\bar{\rho }\tilde{T}} & \overset{\left(4\right)}{\Rightarrow } & & \frac{\bar{p^{\prime }\rho^{\prime }}}{\bar{p}} & = & \frac{\bar{\rho^{\prime 2}}}{\bar{\rho }} & + & \frac{\tilde{\rho^{\prime }T^{\prime \prime }}}{\tilde{T}} \end{array}$$

(A2c) $$\begin{array}{cccccccccccc} \frac{\bar{p^{\prime }T^{\prime \prime }}}{\bar{p}} & \overset{\left(A1b\right)}{=} & \frac{\bar{\rho^{\prime }T^{\prime \prime }}}{\bar{\rho }} & + & \frac{\bar{\rho T^{\prime \prime }T^{\prime \prime }}}{\bar{\rho }\tilde{T}} & \overset{\left(4\right)}{\Rightarrow } & & \frac{\bar{p^{\prime }T^{\prime }}}{\bar{p}} & = & \frac{\bar{\rho^{\prime }T^{\prime }}}{\bar{\rho }} & + & \frac{\tilde{T^{\prime \prime 2}}}{\tilde{T}} \end{array}$$

where we used the definition and properties of Favre averaging (4), including the identity $\bar{{\left(\cdot \right)}^{\prime }{\left[\cdot \right]}^{\prime \prime }}=\bar{{\left(\cdot \right)}^{\prime \prime }{\left[\cdot \right]}^{\prime }}=\bar{{\left(\cdot \right)}^{\prime }{\left[\cdot \right]}^{\prime }}$ (4d)

Comparing (A2) with the corresponding relations (12) that use Reynolds fluctuations only and lead to the easily interpretable system (18), it becomes clear that lumping the higher-order Reynolds correlations into the Favre averages does not simplify the equations. Instead, Reynolds-averaged moments $\left\{\bar{p^{\prime }\rho^{\prime }},\bar{\rho^{\prime }T^{\prime }},\bar{p^{\prime 2}},\bar{\rho^{\prime 2}}\right\}$ coexist with the Favre-averaged moments $\left\{\tilde{p^{\prime }T^{\prime \prime }},\tilde{\rho^{\prime }T^{\prime \prime }},\tilde{T^{\prime \prime 2}}\right\}$. The higher-order compressibility effects in (A2) are lumped into these Favre-averaged moments.

Similarly, squaring and averaging (A1b) yields

(A3) $$\begin{array}{c} {C\;V}_{\;p^{\prime }}^{2}={C\;V}_{\;\rho^{\prime }}^{2}+\frac{\bar{{\left(\rho T^{\prime \prime }\right)}^{2}}}{\rho^{2}{\tilde{T}}^{2}}+\frac{2\bar{\rho \rho^{\prime }T^{\prime \prime }}}{\rho^{2}\tilde{T}} \end{array}$$

which is more difficult to interpret than the Reynolds-averaged expression (16). It contains fewer terms, but $\bar{{\left(\rho T^{\prime \prime }\right)}^{2}}$ and $\bar{\rho \rho^{\prime }T^{\prime \prime }}$ are unusual expressions that require further expansion.

Finally, the ratios

(A4a) $$\begin{array}{ccc} \frac{\sqrt{\tilde{{\left(\cdot \right)}^{\prime \prime 2}}}}{\bar{\left(\cdot \right)}} & \overset{\left(4)\mathrm{and}\;(2\right)}{=} & {C\;V}_{\;{\left(\cdot \right)}^{\prime }}\sqrt{1+c_{\rho^{\prime }{\left(\cdot \right)}^{\prime }{\left(\cdot \right)}^{\prime }}\;{C\;V}_{\;\rho^{\prime }}-c_{\rho^{\prime }{\left(\cdot \right)}^{\prime }}^{2}\;{C\;V}_{\;\rho^{\prime }}^{2}} \end{array}$$

(A4b) $$\begin{array}{ccc} \frac{\sqrt{\tilde{{\left(\cdot \right)}^{\prime \prime 2}}}}{\tilde{\left(\cdot \right)}} & \overset{\left(A4a)\mathrm{and}\;(4a\right)}{=} & {C\;V}_{\;{\left(\cdot \right)}^{\prime }}\frac{\sqrt{1+c_{\rho^{\prime }{\left(\cdot \right)}^{\prime }{\left(\cdot \right)}^{\prime }}\;{C\;V}_{\;\rho^{\prime }}-c_{\rho^{\prime }{\left(\cdot \right)}^{\prime }}^{2}\;{C\;V}_{\;\rho^{\prime }}^{2}}}{1+c_{\rho^{\prime }{\left(\cdot \right)}^{\prime }}{C\;V}_{\;\rho^{\prime }}{C\;V}_{\;{\left(\cdot \right)}^{\prime }}} \end{array}$$

are not coefficients of variation in the pdf of the single variable (·) but involve correlation coefficients with $\rho^{\prime }$ as well (2CCs and 3CCs). To obtain (A4a), we used

(A4c) $$\begin{array}{cc} \frac{\tilde{{\left(\cdot \right)}^{\prime \prime 2}}}{{\bar{\left(\cdot \right)}}^{2}}\overset{\left(4a)\mathrm{and}\;(4b\right)}{=}\frac{\bar{\rho {\left({\left(\cdot \right)}^{\prime }+\bar{{\left(\cdot \right)}^{\prime \prime }}\right)}^{2}}}{\bar{\rho }{\bar{\left(\cdot \right)}}^{2}}= & \frac{\bar{\rho {\left(\cdot \right)}^{\prime 2}+2\rho {\left(\cdot \right)}^{\prime }\bar{{\left(\cdot \right)}^{\prime \prime }}+\rho {\bar{{\left(\cdot \right)}^{\prime \prime }}}^{2}}}{\bar{\rho }{\bar{\left(\cdot \right)}}^{2}}\\ = & \frac{\bar{{\left(\cdot \right)}^{\prime 2}}}{{\bar{\left(\cdot \right)}}^{2}}+\frac{\bar{\rho^{\prime }{\left(\cdot \right)}^{\prime 2}}}{\bar{\rho }{\bar{\left(\cdot \right)}}^{2}}+\frac{2\bar{\rho^{\prime }{\left(\cdot \right)}^{\prime }}\;\;\bar{{\left(\cdot \right)}^{\prime \prime }}+\bar{\rho }{\bar{{\left(\cdot \right)}^{\prime \prime }}}^{2}}{\bar{\rho }{\bar{\left(\cdot \right)}}^{2}}\\ \overset{\left(4c\right)}{=} & \frac{\bar{{\left(\cdot \right)}^{\prime 2}}}{{\bar{\left(\cdot \right)}}^{2}}+\frac{\bar{\rho^{\prime }{\left(\cdot \right)}^{\prime }{\left(\cdot \right)}^{\prime }}}{\bar{\rho }{\bar{\left(\cdot \right)}}^{2}}-\frac{{\bar{{\left(\cdot \right)}^{\prime \prime }}}^{2}}{{\bar{\left(\cdot \right)}}^{2}}\\ \overset{\left(4c\right)}{=} & \frac{\bar{{\left(\cdot \right)}^{\prime 2}}}{{\bar{\left(\cdot \right)}}^{2}}+\frac{\bar{\rho^{\prime }{\left(\cdot \right)}^{\prime }{\left(\cdot \right)}^{\prime }}}{\bar{\rho }{\bar{\left(\cdot \right)}}^{2}}-\frac{{\bar{\rho^{\prime }{\left(\cdot \right)}^{\prime }}}^{2}}{{\bar{\rho }}^{2}{\bar{\left(\cdot \right)}}^{2}}\\ \overset{\left(2\right)}{=} & {C\;V}_{\;{\left(\cdot \right)}^{\prime }}^{2}\left(1+c_{\rho^{\prime }{\left(\cdot \right)}^{\prime }{\left(\cdot \right)}^{\prime }}\;{C\;V}_{\;\rho^{\prime }}-c_{\rho^{\prime }{\left(\cdot \right)}^{\prime }}^{2}\;{C\;V}_{\;\rho^{\prime }}^{2}\right) \end{array}$$

## Appendix B. Reynolds and Mach Number Effects in a Turbulent Plane Channel Flow

In addition to the selected $\left(Re_{\tau^{★}},{\bar{M}}_{{\mathrm{CL}}_{x}}\right)$-examples used to illustrate the mathematical relations developed in the body of the paper, a more systematic study of the dependence of higher-order correlations of the fluctuations in the thermodynamic variables $\left\{p^{\prime },\rho^{\prime },T^{\prime }\right\}$ on the flow’s Mach and Reynolds numbers is undertaken. We use the HCB [17] friction Reynolds number $Re_{\tau^{★}}$ ([10], (2.1b), p. A19-5) and the average centerline streamwise Mach number ${\bar{M}}_{{\mathrm{CL}}_{x}}$ ([10], (2.4), p. A19-9) to define the flow conditions in the database [12]. Data are plotted against the HCB-scaled nondimensional wall-distance $y^{★}$ ([10], (2.1b), p. A19-5). The terms inner, overlap and wake are used to denote the corresponding wall-regions [41].

Notice first that the profiles for ${\bar{M}}_{{\mathrm{CL}}_{x}}\;\in \;\left\{0.33,0.81\right\}$ at $Re_{\tau^{★}}\;≊\;110$ (Figure A1a, Figure A3a, Figure A5a and Figure A7a) are nearly identical for all of the correlations studied, revealing that an asymptotic low-Mach-number thermodynamic turbulence structure is reached already at ${\bar{M}}_{{\mathrm{CL}}_{x}}\;\leq \;0.8$.

The profiles of pressure skewness $S_{p^{\prime }}$ exhibit clearly identified zones. At $Re_{\tau^{★}}\;≊\;1000$ (Figure A1d), a plateau of near-0 skewness ($1\;⪅\;y^{★}\;⪅\;10$) suddenly drops to a plateau of negative skewness ($20\;⪅\;y^{★}\;⪅\;400$), followed by a wavelike zone in the centerline region. These regions are clearly identified at a lower $Re_{\tau^{★}}$ as well (Figure A1a–c), with the negative skewness plateau terminating at correspondingly lower values of $y^{★}$. With increasing ${\bar{M}}_{{\mathrm{CL}}_{x}}$, $S_{p^{\prime }}$ becomes less negative, with the near-wall plateau ($1\;⪅\;y^{★}\;⪅\;10$) moving from slightly negative at the low-Mach-number limit to slightly positive (Figure A1a–d). The near-wall ($1\;⪅\;y^{★}\;⪅\;10$) Mach effect is more pronounced at a low $Re_{\tau^{★}}$ (Figure A1a), in agreement with the appearance of very-near-wall organized pressure waves as ${\bar{M}}_{{\mathrm{CL}}_{x}}$ increases [10]. Temperature skewness $S_{T^{\prime }}$ (Figure A1i–l) changes from values close to +1 at $y^{★}\;≊\;1$ to values close to −1.5 at the centerline, switching signs at $y^{★}\;≊\;10$. There is very little ${\bar{M}}_{{\mathrm{CL}}_{x}}$-influence on $S_{T^{\prime }}$, except for a slight decrease very near the wall ($1\;⪅\;y^{★}\;⪅\;10$) for the strongly compressible $\left(Re_{\tau^{★}},{\bar{M}}_{{\mathrm{CL}}_{x}}\right)\;=\;\left(113,2,49\right)$ flow (Figure A1i).

The profiles of density skewness $S_{\rho^{\prime }}$ show the strongest $Re_{\tau^{★}}$ influence (Figure A1e–h), and this trend is more clearly observed when comparing the $S_{\rho^{\prime }}$-profiles at nearly constant ${\bar{M}}_{{\mathrm{CL}}_{x}}$ (Figure A2e–h). The DNS data at $Re_{\tau^{★}}\;≊\;1000$ (Figure A1h) suggest that with an increasing $Re_{\tau^{★}}$, a positive $S_{\rho^{\prime }}$ plateau will probably form in the overlap region, similarly to $S_{p^{\prime }}$ (Figure A1d). This is corroborated by the plots at constant ${\bar{M}}_{{\mathrm{CL}}_{x}}$ (Figure A2f–h). Computations at higher $Re_{\tau^{★}}$ are necessary to fully substantiate this conjecture. In contrast with $S_{p^{\prime }}$, where the different zones are clearly identified at all $Re_{\tau^{★}}$, the $S_{\rho^{\prime }}$ plateau is not visible yet at the lower $Re_{\tau^{★}}$ (Figure A1e–g). Notice that $S_{\rho^{\prime }}$ changes sign from positive to negative towards the centerline (Figure A1e–h) at approximately the end of the overlap $S_{p^{\prime }}$ plateau (Figure A1a–d). The plots at constant ${\bar{M}}_{{\mathrm{CL}}_{x}}$ show that the pressure skewness $S_{p^{\prime }}$ (Figure A2a–d) reaches a Re-asymptotic inner/overlap $y^{★}$-law already at $Re_{\tau^{★}}\;≊\;250$, whereas the temperature skewness $S_{T^{\prime }}$ plateau has not reached an asymptotic level yet (Figure A2i–l).

Similarly to $S_{T^{\prime }}$, the ${\bar{M}}_{{\mathrm{CL}}_{x}}$ effect on the temperature kurtosis $F_{T^{\prime }}$ is small (Figure A3i–l), even in the strongly compressible near-wall region at $\left(Re_{\tau^{★}},{\bar{M}}_{{\mathrm{CL}}_{x}}\right)\;=\;\left(113,2,49\right)$ (Figure A3i). The small ${\bar{M}}_{{\mathrm{CL}}_{x}}$ influence, at constant $Re_{\tau^{★}}$, both on skewness $S_{T^{\prime }}$ (Figure A1i–l) and on kurtosis $F_{T^{\prime }}$ (Figure A3i–l) suggests that the temperature pdfs $f_{T^{\prime }}$ are not very sensitive to ${\bar{M}}_{{\mathrm{CL}}_{x}}$ but rather depend on the thermal wall condition. There is, nonetheless, in the centerline region a trend of a decreasing (more negative) $S_{T^{\prime }}$ (Figure A1j–l) and an increasing $F_{T^{\prime }}$ (Figure A3j–l) with a decreasing ${\bar{M}}_{{\mathrm{CL}}_{x}}$. This trend seems more pronounced with an increasing $Re_{\tau^{★}}$ (Figure A1l and Figure A3l). On the contrary, the pressure kurtosis $F_{p^{\prime }}$ is quite sensitive to ${\bar{M}}_{{\mathrm{CL}}_{x}}$. The low $Re_{\tau^{★}}\;≊\;110$ data (Figure A3a) indicate that $F_{p^{\prime }}$ decreases with increasing ${\bar{M}}_{{\mathrm{CL}}_{x}}$ for ${\bar{M}}_{{\mathrm{CL}}_{x}}\;\leq \;2$, but noticeable compressibility effects appear near the wall at ${\bar{M}}_{{\mathrm{CL}}_{x}}\;=\;2.49$. The $F_{p^{\prime }}$ data at constant ${\bar{M}}_{{\mathrm{CL}}_{x}}\;≊\;2$ suggest a Re-asymptotic inner/overlap $y^{★}$-law (Figure A4d), but the lower ${\bar{M}}_{{\mathrm{CL}}_{x}}$ data (Figure A4a–c) indicate that this law has a $Re_{\tau^{★}}$-dependence. Computations at higher $Re_{\tau^{★}}$ are required to elucidate this point.

The region of platykurtic distributions (negative excess kurtosis, $F_{{\left(\cdot \right)}^{\prime }}\;<\;3$) of $F_{T^{\prime }}$ (Figure A3i–l) has a minimum kurtosis close to 2 at $y^{★}\;≊\;10$. This region is practically independent of ${\bar{M}}_{{\mathrm{CL}}_{x}}$ (Figure A3i–l) but extends to a higher $y^{★}$ with an increasing $Re_{\tau^{★}}$ (Figure A4i–l). The density kurtosis $F_{\rho^{\prime }}$ also exhibits a region of platykurtic distributions at approximately (but not exactly) the same locations as $F_{T^{\prime }}$ but with an ${\bar{M}}_{{\mathrm{CL}}_{x}}$ dependence (Figure A3e–h), also visible in the centerline region. These differences in the profiles of $\left\{F_{\rho^{\prime }},F_{T^{\prime }}\right\}$ notwithstanding, $F_{\rho^{\prime }}$ (Figure A4e–h) and $F_{T^{\prime }}$ (Figure A4i–l) are quite similar near the wall but differ in the outer part of the flow.

The profiles of the 3CCs $\left\{c_{p^{\prime }\rho^{\prime }T^{\prime }},c_{\rho^{\prime }\rho^{\prime }T^{\prime }},c_{\rho^{\prime }T^{\prime }T^{\prime }},c_{p^{\prime }\rho^{\prime }\rho^{\prime }},c_{p^{\prime }p^{\prime }\rho^{\prime }},c_{p^{\prime }p^{\prime }T^{\prime }},c_{p^{\prime },T^{\prime }T^{\prime }}\right\}$, at constant $Re_{\tau^{★}}$ (Figure A5 and Figure A7), show ${\bar{M}}_{{\mathrm{CL}}_{x}}$ dependence, especially in the near-wall region ($1\;⪅\;y^{★}\;⪅\;10$), but also near the centerline. The profiles of $c_{\rho^{\prime }T^{\prime }T^{\prime }}$ are an exception because they appear to be practically ${\bar{M}}_{{\mathrm{CL}}_{x}}$-independent for $y^{★}\;⪆\;100$ (Figure A5i–l). The ${\bar{M}}_{{\mathrm{CL}}_{x}}$ influence in the wake region is important for all of the 3CCs containing $p^{\prime }$, $c_{p^{\prime }\rho^{\prime }T^{\prime }}$ (Figure A5a–d) and $\left\{c_{p^{\prime }\rho^{\prime }\rho^{\prime }},c_{p^{\prime }p^{\prime }\rho^{\prime }},c_{p^{\prime }p^{\prime }T^{\prime }},c_{p^{\prime },T^{\prime }T^{\prime }}\right\}$ (Figure A7), while it is much smaller for $c_{\rho^{\prime }\rho^{\prime }T^{\prime }}$ (Figure A5e–h). At constant ${\bar{M}}_{{\mathrm{CL}}_{x}}$ (Figure A6 and Figure A8), all of the 3CCs appear to progressively form, with increasing $Re_{\tau^{★}}$, an inner/overlap $y^{★}$-law. The data are very convincing regarding $c_{p^{\prime }\rho^{\prime }T^{\prime }}$ (Figure A6b–d) and $c_{p^{\prime }p^{\prime }\rho^{\prime }}$ (Figure A8f–h), for which collapsing of the profiles, for progressively larger $Re_{\tau^{★}}$, that extends to the overlap region is clearly observed. For the other 3CCsm the trend of forming an overlap law is rather obvious (Figure A6 and Figure A8), but the highest $Re_{\tau^{★}}\;≊\;1000$ available in the database [12] is probably not large enough yet to elucidate whether an overlap law will appear, highlighting again the need for even higher $Re_{\tau^{★}}$ computations. Notice that the progressive formation of an overlap law for $c_{\rho^{\prime }\rho^{\prime }T^{\prime }}$ (Figure A6f–h) and $c_{\rho^{\prime }T^{\prime }T^{\prime }}$ (Figure A6j–l) is very similar to that observed regarding the individual skewnesses $S_{\rho^{\prime }}$ (Figure A2f–h) and $S_{T^{\prime }}$ (Figure A2j–l).

The data reported in this Appendix B are specific to the very-cold-wall [11] conditions of canonical TPC flow [5]. The influence of the thermal wall condition on the thermodynamic turbulence structure requires specific study with new DNS data.

## Appendix C. Power Series and Amplitude Effects

Independently of the exact relations between the correlation coefficients (Section 4), we will report here another identity resulting from the instantaneous EoS (1). Dividing (1) by its Reynolds average (6a) yields

(A5) $$\begin{array}{c} \frac{p}{\bar{p}}=\frac{\rho }{\bar{\rho }}\frac{T}{\bar{T}}\frac{1}{1+c_{\rho^{\prime }T^{\prime }}\;{C\;V}_{\;\rho^{\prime }}\;{C\;V}_{\;T^{\prime }}}\Rightarrow \ln\frac{p}{\bar{p}}=\ln\frac{\rho }{\bar{\rho }}+\ln\frac{T}{\bar{T}}-\ln\left(1+c_{\rho^{\prime }T^{\prime }}\;{C\;V}_{\;\rho^{\prime }}\;{C\;V}_{\;T^{\prime }}\right)\Rightarrow \\ \ln\left(\;1\;+\;\frac{p^{\prime }}{\bar{p}}\;\right)=\ln\left(\;1\;+\;\frac{\rho^{\prime }}{\bar{\rho }}\right)\;+\;\ln\left(1+\frac{T^{\prime }}{\bar{T}}\right)\;-\;\ln\left(\;1\;+\;c_{\rho^{\prime }T^{\prime }}\;{C\;V}_{\;\rho^{\prime }}\;{C\;V}_{\;T^{\prime }}\;\right)\;\forall \;\vec{x},t \end{array}$$

which involves the instantaneous relative levels of fluctuation. Provided that

(A6) $$\begin{array}{c} |\frac{p^{\prime }}{\bar{p}}\left|<1\;;\;\right|\frac{\rho^{\prime }}{\bar{\rho }}\left|<1\;;\;\right|\frac{T^{\prime }}{\bar{T}}|<1 \end{array}$$

identity (A5) can be Taylor-expanded, using $-1\;<\;x\;\leq \;1\;\Rightarrow \;\ln\left(1+x\right)\;=\;\sum_{\ell =1}^{\infty }{\left(-1\right)}^{\ell +1}x^{\ell }/\ell$ ([42], p. 140), as

(A7) $$\begin{array}{c} \sum_{n=1}^{\infty }\left(\frac{{\left(-1\right)}^{n+1}}{n}\left[{\left(\frac{p^{\prime }}{\bar{p}}\right)}^{\;\;n}-{\left(\frac{\rho^{\prime }}{\bar{\rho }}\right)}^{\;\;n}-{\left(\frac{T^{\prime }}{\bar{T}}\right)}^{\;\;n}\right]\right)\overset{\left(A5)\mathrm{and}\;(A6\right)}{=}-\ln\left(1+c_{\rho^{\prime }T^{\prime }}\;{C\;V}_{\;\rho^{\prime }}\;{C\;V}_{\;T^{\prime }}\right)\;\forall \;\vec{x},t \end{array}$$

showing that the infinite power series of the instantaneous fluctuations sum up, $\forall \;\vec{x},t$, to the average quantity $-\ln\left(1+c_{\rho^{\prime }T^{\prime }}\;{C\;V}_{\;\rho^{\prime }}\;{C\;V}_{\;T^{\prime }}\right)$. It is noteworthy that (A7) involves power series of instantaneous fluctuations, not just averages. The correlation $c_{\rho^{\prime }T^{\prime }}$ stands out as a very important parameter in the analysis of the thermodynamic turbulence structure, in line with previous work [1,4].

Statistical equations are readily generated from (A7). Multiplying by $p^{\prime }/\bar{p}$ and averaging yields

(A8a) $$\begin{array}{c} \sum_{n=1}^{\infty }\left(\frac{{\left(-1\right)}^{n+1}}{n}\left[\bar{{\left(\frac{p^{\prime }}{\bar{p}}\right)}^{\;\;n+1}}-\bar{\frac{p^{\prime }}{\bar{p}}{\left(\frac{\rho^{\prime }}{\bar{\rho }}\right)}^{\;\;n}}-\bar{\frac{p^{\prime }}{\bar{p}}{\left(\frac{T^{\prime }}{\bar{T}}\right)}^{\;\;n}}\right]\right)=0 \end{array}$$

while averaging the instantaneous relation (A7), the following identity between individual moments is obtained:

(A8b) $$\begin{array}{cc} \;\;\;\underset{=\frac{{s|}_{\left(\bar{p},\bar{T}\right)}{-s|}_{\left(\bar{\rho },\bar{T}\right)}}{R_{g}}}{\underset{︸}{\sum_{n=2}^{\infty }\left(\frac{{\left(-1\right)}^{n+1}}{n}\left[\bar{{\left(\frac{p^{\prime }}{\bar{p}}\right)}^{n}}-\bar{{\left(\frac{\rho^{\prime }}{\bar{\rho }}\right)}^{n}}-\bar{{\left(\frac{T^{\prime }}{\bar{T}}\right)}^{n}}\right]\right)}}= & -\ln\left(1+c_{\rho^{\prime }T^{\prime }}\;{C\;V}_{\;\rho^{\prime }}\;{C\;V}_{\;T^{\prime }}\right) \end{array}$$

Notice that the infinite series on the LHS of (A8b) are identified from ([14], (A10), p. 19) as the nondimensional entropy difference between states $\left(\bar{p},\bar{T}\right)$ and $\left(\bar{\rho },\bar{T}\right)$, which is obviously $O({C\;V}_{\;\rho^{\prime }}^{2},{C\;V}_{\;T^{\prime }}^{2},{C\;V}_{\;p^{\prime }}^{2})$, in line with the differences $\left(s{|}_{\left(\bar{p},\bar{T}\right)}-\bar{s}\right)$ and $\left(s{|}_{\left(\bar{\rho },\bar{T}\right)}-\bar{s}\right)$ ([14], (A6b, A9b), pp. 18–19) or $\left(s{|}_{\left(\bar{p},\bar{\rho }\right)}-\bar{s}\right)$ ([43], p. 224). We obtain in this way a closed-form expression for the entropy difference between states $\left(\bar{p},\bar{T}\right)$ and $\left(\bar{\rho },\bar{T}\right)$:

(A9) $$\begin{array}{c} \frac{{s|}_{\left(\bar{p},\bar{T}\right)}{-s|}_{\left(\bar{\rho },\bar{T}\right)}}{R_{g}}\overset{\left(A8b\right)}{=}-\ln\left(1+c_{\rho^{\prime }T^{\prime }}\;{C\;V}_{\;\rho^{\prime }}\;{C\;V}_{\;T^{\prime }}\right)\overset{\left(4a\right)}{=}-\ln\frac{\tilde{T}}{\bar{T}} \end{array}$$

With hindsight, (A9) can be obtained through the integration of $ds/R_{g}\;=\;c_{v}\left(T\right)dT/\left(R_{g}T\right)\;-\;d\rho /\rho$ [14].

Although at a given location in the flow field the ratios between CVs vary with the type and conditions of the flow ([4], Fig. 3, p. 458), generally, $\left\{{C\;V}_{\;p^{\prime }},{C\;V}_{\;\rho^{\prime }},{C\;V}_{\;T^{\prime }}\right\}$ are in the same order of magnitude ([2], Fig. 5, p. 720) and <1 (smaller than unity), so that (n+1)oTs are expected to be smaller than *n*oTs. This is especially true in weakly compressible turbulence conditions, $\max\left({C\;V}_{\;p^{\prime }},{C\;V}_{\;\rho^{\prime }},{C\;V}_{\;T^{\prime }}\right)\;\ll \;1$.

Equation (A8a) relates a series involving the moments of $p^{\prime }$ with a series of moments of joint $\left(p^{\prime },\rho^{\prime }\right)$-statistics and $\left(p^{\prime },T^{\prime }\right)$-statistics. Using CVs (2a) and CCs (2b), the first few terms of (A8a) read as

(A10a) CVp′−12Sp′3CVp′2+13Fp′4CVp′3+⋯∼(A8a)cp′ρ′CVρ′−12cp′ρ′ρ′CVρ′2+13cp′ρ′ρ′ρ′CVρ′3+⋯+cp′T′CVT′−12cp′T′T′CVT′2+13cp′T′T′T′CVT′3+⋯

where the leading order-1 terms ${C\;V}_{\;p^{\prime }}\;≊\;c_{p^{\prime }\rho^{\prime }}\;{C\;V}_{\;\rho^{\prime }}+c_{p^{\prime }T^{\prime }}\;{C\;V}_{\;T^{\prime }}$ are the well-known linearized relation ([4], (4.1a), p. 460) obtained upon multiplying the fluctuating EoS (7) by $p^{\prime }$, averaging and dropping the HoTs (keeping only 1oTs).

Equation (A8b) is a series involving CVs, skewnesses, flatnesses, superskewnesses, superflatnesses and HoTs. The term $-\ln\left(1+c_{\rho^{\prime }T^{\prime }}\;{C\;V}_{\;\rho^{\prime }}\;{C\;V}_{\;T^{\prime }}\right)$ can also be Taylor-expanded using $\ln\left(1+x\right)\;=\;\sum_{\ell =1}^{\infty }{\left(-1\right)}^{\ell +1}x^{\ell }/\ell$ ([42], p. 140). The first few terms of (A8b) read as

(A10b) −12(CVp′2−CVρ′2−CVT′2)+13(Sp′3CVp′3−Sρ′3CVρ′3−ST′3CVT′3)−14(Fp′4CVp′4−Fρ′4CVρ′4−FT′4CVT′4)+15(Sp′5CVp′5−Sρ′5CVρ′5−ST′5CVT′5)−16(Fp′6CVp′6−Fρ′6CVρ′6−FT′6CVT′6)+⋯∼(A8b)−ln1+cρ′T′CVρ′CVT′∼−cρ′T′CVρ′CVT′+12cρ′T′2CVρ′2CVT′2−13cρ′T′3CVρ′3CVT′3+⋯

Notice that the expansion of $\ln\left(1+c_{\rho^{\prime }T^{\prime }}\;{C\;V}_{\;\rho^{\prime }}\;{C\;V}_{\;T^{\prime }}\right)$ involves only 2noTs, so that successive corrections of the series on the LHS of (A8b) should be considered with a step of 2, i.e.,

(A11) $$\begin{array}{c} \sum_{k=1}^{\infty }\left(\frac{1}{2k\;\;-\;\;1}\;\left[\bar{{\left(\frac{p^{\prime }}{\bar{p}}\right)}^{\;\;2k\;-\;1}}\;-\;\bar{{\left(\frac{\rho^{\prime }}{\bar{\rho }}\right)}^{\;\;2k\;-\;1}}\;-\;\bar{{\left(\frac{T^{\prime }}{\bar{T}}\right)}^{\;\;2k\;-\;1}}\right]-\frac{1}{2k}\;\left[\bar{{\left(\frac{p^{\prime }}{\bar{p}}\right)}^{\;\;2k}}\;-\;\bar{{\left(\frac{\rho^{\prime }}{\bar{\rho }}\right)}^{\;\;2k}}\;-\;\bar{{\left(\frac{T^{\prime }}{\bar{T}}\right)}^{\;\;2k}}\right]+\frac{{\left(-1\right)}^{k+1}}{k}{\left(c_{\rho^{\prime }T^{\prime }}\;{C\;V}_{\;\rho^{\prime }}\;{C\;V}_{\;T^{\prime }}\right)}^{k}\right)\overset{\left(A8b\right)}{=}0 \end{array}$$

where, of course, for k=1 the (2k−1)-order=1-order moment is 0.

To illustrate the increasing, with an increasing flow Mach number, importance of the HoTs in (A10b), we consider successive approximations to ${C\;V}_{\;p^{\prime }}$, obtained by summing n∈{1,2,3} terms in the series (A11) and solving for ${{C\;V}_{\;p^{\prime }}}_{\;(2n-1)}$:

(A12a) $$\begin{array}{cc} \underset{∼{C\;V}_{\;p^{\prime }}\left(1+1\mathrm{oTs}\right)}{\underset{︸}{{{C\;V}_{\;p^{\prime }}}_{\;(1)}}}\;\overset{\left(A11\right)}{:=} & \sqrt{{C\;V}_{\;\rho^{\prime }}^{2}+{C\;V}_{\;T^{\prime }}^{2}+2\;c_{\rho^{\prime }T^{\prime }}\;{C\;V}_{\;\rho^{\prime }}\;{C\;V}_{\;T^{\prime }}}\overset{\left(2\right)}{=}\sqrt{\frac{\bar{\rho^{\prime 2}}}{{\bar{\rho }}^{2}}+\frac{\bar{T^{\prime 2}}}{{\bar{T}}^{2}}+2\;\frac{\bar{\rho^{\prime }T^{\prime }}}{\bar{\rho }\;\bar{T}}} \end{array}$$

(A12b) CVp′(3)︸∼CVp′1+3oTs:=(A11)CVp′(1)+23Sp′3CVp′3−Sρ′3CVρ′3−ST′3CVT′3−24Fp′4CVp′4−Fρ′4CVρ′4−FT′4CVT′4−22cρ′T′2CVρ′2CVT′2=(2)CVp′(1)+23p′3¯p¯3−ρ′3¯ρ¯3−T′3¯T¯3−24p′4¯p¯4−ρ′4¯ρ¯4−T′4¯T¯4−22ρ′T′¯ρ¯T¯2

(A12c) CVp′(5)︸∼CVp′1+5oTs:=(A11)CVp′(3)+25Sp′5CVp′5−Sρ′5CVρ′5−ST′5CVT′5−26Fp′6CVp′6−Fρ′6CVρ′6−FT′6CVT′6+23cρ′T′3CVρ′3CVT′3=(2)CVp′(3)+25p′5¯p¯5−ρ′5¯ρ¯5−T′5¯T¯5−26p′6¯p¯6−ρ′6¯ρ¯6−T′6¯T¯6+23ρ′T′¯ρ¯T¯3

These progressively more accurate expressions (A12) are assessed using DNS data for a compressible turbulent plane channel (TPC) flow at representative Reynolds and Mach numbers (centerline streamwise Mach number ${\bar{M}}_{{\mathrm{CL}}_{x}}$ and HCB friction Reynolds number $Re_{\tau^{★}}$) from the datasets available in [12]. Single-variable moments up to order-6 were calculated in the database from sampled single-variable pdfs, whereas all $\left(p^{\prime },\rho^{\prime },T^{\prime }\right)$-moments up to order-3 were independently sampled through statistical xzt-averaging.

At subsonic ${\bar{M}}_{{\mathrm{CL}}_{x}}\;\in \;\left\{0.32,0.81\right\}$ (Figure A9a,b), the well-known [1,4] leading-order approximation (A12a) which expresses ${C\;V}_{\;p^{\prime }}$ from 2-moments of $\left(\rho^{\prime },T^{\prime }\right)$ is practically indistinguishable from the exact ${C\;V}_{\;p^{\prime }}$ profiles. At a centerline streamwise Mach number ${\bar{M}}_{{\mathrm{CL}}_{x}}\;=\;1.51$ (Figure A9c), the leading-order approximation (A12a) is quite accurate, with a very slight discrepancy in the buffer region ($5\;⪅\;y^{★}\;⪅\;40$) which is corrected when retaining the order-3 terms (A12b). The discrepancy on the leading-order approximation in the buffer zone ($5\;⪅\;y^{★}\;⪅\;40$) is more pronounced at ${\bar{M}}_{{\mathrm{CL}}_{x}}\;≊\;2$ (Figure A9d,e) and is again corrected when retaining the order-3 terms (A12b). At a higher ${\bar{M}}_{{\mathrm{CL}}_{x}}\;=\;2.49$ (Figure A9f), the leading-order approximation (A12a) is unsatisfactory, and although the discrepancy is practically removed by retaining the order-3 terms (A12b), there is still a small improvement by including the order-5 terms (A12c), with ${{C\;V}_{\;p^{\prime }}}_{\;(5)}$ practically indistinguishable from the exact ${C\;V}_{\;p^{\prime }}$ profiles (Figure A9f).
